# Transforming carbon dioxide into jet fuel using an organic combustion-synthesized Fe-Mn-K catalyst

**DOI:** 10.1038/s41467-020-20214-z

**Published:** 2020-12-22

**Authors:** Benzhen Yao, Tiancun Xiao, Ofentse A. Makgae, Xiangyu Jie, Sergio Gonzalez-Cortes, Shaoliang Guan, Angus I. Kirkland, Jonathan R. Dilworth, Hamid A. Al-Megren, Saeed M. Alshihri, Peter J. Dobson, Gari P. Owen, John M. Thomas, Peter P. Edwards

**Affiliations:** 1 KACST-Oxford Centre of Excellence in Petrochemicals, Inorganic Chemistry Laboratory, University of Oxford, South Parks Road, Oxford, OX1 3QR UK; 2 Department of Materials, University of Oxford, Parks Roads, Oxford, OX1 3PH UK; 3 Merton College, University of Oxford, Merton Street, Oxford, OX1 4JD UK; 4 Cardiff Catalysis Institute, School of Chemistry, Cardiff University, Cardiff, UK; 5 Harwell-XPS – The EPSRC National Facility for Photoelectron Spectroscopy, Research Complex at Harwell (RCaH), Didcot, Oxon, OX11 0FA UK; 6 Electron Physical Sciences Imaging Centre, Diamond Lightsource Ltd., Didcot, Oxford, OX11 0DE UK; 7 Materials Division, King Abdulaziz City for Science and Technology, Riyadh, 11442 Kingdom of Saudi Arabia; 8 The Queen’s College, University of Oxford, Oxford, OX1 4AW UK; 9 Annwvyn Solutions, 76 Rochester Avenue, Bromley, Kent BR1 3DW UK; 10 Department of Materials Science and Metallurgy, University of Cambridge, 27 Charles Babbage Road, Cambridge, CB3 0FS UK

**Keywords:** Catalysis, Energy, Green chemistry

## Abstract

With mounting concerns over climate change, the utilisation or conversion of carbon dioxide into sustainable, synthetic hydrocarbons fuels, most notably for transportation purposes, continues to attract worldwide interest. This is particularly true in the search for sustainable or renewable aviation fuels. These offer considerable potential since, instead of consuming fossil crude oil, the fuels are produced from carbon dioxide using sustainable renewable hydrogen and energy. We report here a synthetic protocol to the fixation of carbon dioxide by converting it directly into aviation jet fuel using novel, inexpensive iron-based catalysts. We prepare the Fe-Mn-K catalyst by the so-called Organic Combustion Method, and the catalyst shows a carbon dioxide conversion through hydrogenation to hydrocarbons in the aviation jet fuel range of 38.2%, with a yield of 17.2%, and a selectivity of 47.8%, and with an attendant low carbon monoxide (5.6%) and methane selectivity (10.4%). The conversion reaction also produces light olefins ethylene, propylene, and butenes, totalling a yield of 8.7%, which are important raw materials for the petrochemical industry and are presently also only obtained from fossil crude oil. As this carbon dioxide is extracted from air, and re-emitted from jet fuels when combusted in flight, the overall effect is a carbon-neutral fuel. This contrasts with jet fuels produced from hydrocarbon fossil sources where the combustion process unlocks the fossil carbon and places it into the atmosphere, in longevity, as aerial carbon - carbon dioxide.

## Abstract (web summary)

Design and discovery of catalysts to make clean energy and mitigate the harmful effect of greenhouse gases remains a massive challenge. Here the authors report a combustion-synthesised iron-based catalyst of high activity and selectivity for directly converting CO_2_ to aviation jet fuel.

## Introduction

For more than a century our industrial society and humankind’s prosperity, wealth and well-being, have been based on the combustion of hydrocarbon fossil fuels. However, it is abundantly clear this has disturbed the natural environment by the emission of greenhouse gases, most notably carbon dioxide (CO_2_), nitrous oxide, and methane^1^. Nevertheless, the use of fossil fuels continues to grow with an expected annual increase of 1.3% to 2030^2^, continually exacerbating this problem in the form of climate change Air transport, playing a significant role in the modern world in worldwide social contact, business and marketing, is a recognized source of high CO_2_ emissions^3^.

Jet fuel, the generic name for the aviation fuels used in gas-turbine powered aircraft has as its main components linear and branched alkanes and cycloalkanes with a typical carbon chain-length distribution of C_8_–to- C_18_, and where the ideal carbon chain length is C_8_–C_16_^4^.

Given these recognised environmental concerns, it is now imperative to develop clean, energy-efficient technologies for producing sustainable or renewable aviation fuels^5^.

Converting CO_2_ into fuels and high value-added chemicals has attracted significant worldwide interest in the past few years, as it not only contributes to mitigating greenhouse gas emissions but also produces valuable chemical commodities^6–24^. As such, CO_2_ conversion and utilization should be taken both an integral and important part of greenhouse gas control and sustainable development.

Nevertheless, the activation of CO_2_ is extremely challenging; CO_2_ is a fully oxidized, thermodynamically stable and chemically inert molecule. Furthermore, hydrocarbon synthesis via the hydrogenation of CO_2_ usually favours the formation of short-chain, rather than desirable long-chain, hydrocarbons. Hence most of the research in this area have focused on the selective hydrogenation of CO_2_ to CH_4_, the oxygenates, CH_3_OH, HCOOH, and light olefins (C_2_–C_4_ olefins)^22–41^, There have been limited studies on producing liquid hydrocarbons of molecularity C_5+_^42–44^.

There are two ways to convert CO_2_ to liquid hydrocarbons; an indirect route, which converts CO_2_ to CO or methanol and subsequently into liquid hydrocarbons, or the direct CO_2_ hydrogenation route, which is usually described as a combination of the reduction of CO_2_ to CO via the reverse water gas shift (RWGS) reaction and the subsequent hydrogenation of CO to long-chain hydrocarbons via Fischer-Tropsch synthesis (FTS)^45^. Jet fuel can then be obtained from the products after industrially recognized treatments such as distillation or hydro-isomerization. The second, direct route is generally recognized as being more economical and environmentally acceptable as it involves fewer chemical process steps, and the overall energy consumption for the entire process is lower^46^.

The relevant chemical reactions for hydrocarbon fuel production are:

Hydrogenation of CO_2_:

$${\mathrm{CO}}_2 + 3{\mathrm{H}}_2 \rightleftarrows - \left( {{\mathrm{CH}}_2} \right) - + 2{\mathrm{H}}_2{\mathrm{O}}\left( {{\Delta} H_{298}^0 = - 125\;{\mathrm{kJ}}\;{\mathrm{mol}}^{ - 1}} \right);$$

The RWGS reaction:

$${\mathrm{CO}}_2 + {\mathrm{H}}_2 \rightleftarrows {\mathrm{CO}} + {\mathrm{H}}_2{\mathrm{O}}\left( {{\Delta} H_{298}^0 = + 41\;{\mathrm{kJ}}\;{\mathrm{mol}}^{ - 1}} \right),\,{\mathrm{and}}$$

The FTS reaction:

$${\mathrm{CO}} + 2{\mathrm{H}}_2 \rightleftarrows - ({\mathrm{CH}}_2) - + {\mathrm{H}}_2{\mathrm{O}}\left( {{\Delta} H_{298}^0 = - 166\;{\mathrm{kJ}}\;{\mathrm{mol}}^{ - 1}} \right)$$

The direct conversion of CO_2_ into fuels through these various reactions has attracted great attention in recent years, and a compilation of some of these investigations is highlighted in Table 1. However, there are few reports of the direct catalytic conversion of CO_2_ to jet fuel range hydrocarbons^20,47^. The key to advancing this process is to search for a highly efficient inexpensive catalyst, that can preferentially synthesise the target hydrocarbon range of interest^48^. Iron-based catalysts, widely used in both the RWGS and FTS reactions, are typically prepared by chemical co-precipitation routes, which unfortunately consumes significant amounts of water^49–52^.

**Table 1:** Some typical catalysts performance for the direct conversion of CO_2_ into hydrocarbon fuels; a brief literature overview.

Catalyst	Preparation method	GHSV^a^	H_2_: CO_2_	P/MPa	T/°C	CO_2_ Conv. /%	CO Select./%	Distribution of hydrocarbons /C-mol %			C_5+_yield%	Ref.
								C_1_	C_2_-C_4_	C_5+_		
CuFeO_2_	Hydrothermal	1800	3	1	300	17.3	31.7	2.7	31.0	66.3	7.8	^16^
Fe-Co/K/Al_2_O_3_	Pore-filling incipient wetness impregnation	3600	3	1.1	300	31.0	18	13	69 ^b^	-	20	
In_2_O_3_/HZSM-5	Granule stacking	9000	3	3	340	13.1	44.8	1	20.4	78.6	5.7	^37^
Na–Fe_3_O_4_/HZSM-5	Granule mixing	4000	3	3	320	33.6	14.2	7.9	18.4	73.7	21.2	^39^
Na–Fe_3_O_4_/HZSM-5	Granule mixing	4000	1	3	320	22.0	20.1	4.0	16.6	79.4	14.0	^39^
FeNa	One-pot	2 000	3	3	320	40.5	13.5	15.8	54.1	30.1	10.5	^85^
Na-Fe_3_O_4_/HMCM-22	Granule mixing	4000	2	3	320	25.9	17.1	9	10^c^	82^d^	17.6^e^	^86^
Fe_2_O_3__CT600	Template-assisted	1140	3	1.5	350	40	15	14.1	43.5	42.4	14.4	^87^
Co_6_/MnO_*x*_	Coprecipitation	–	1	8	200	15.3	0.4	46.6^f^	53.4	8.1		^88^
Zn-Cr(3:1)[C4]/HY	Physically mixing	3000	3	4.9	400	31.7	85.8	6.8	68.2	25.0	1.1	^89^
Fe–Zn–Zr@HZSM-5–Hbeta	Cladding	3000	3	5	340	14.9	38.6	1.5	71.7	26.8	2.5	^90^
F–Mn–K	Organic combustion	2400	3	1	300	38.2	5.6	10.4	27.7	61.9	22.3	This work

^a^Unit in ml g_cat_^−1^ h ^−1^.

^b^Selectivity for C_2+_, main carbon number range of liquid product was approximately corresponding to jet fuel, but no data of selectivity/yield available.

^c^Selectivity for C_2_–C_3_.

^d^Selectivity for C_4+_.

^e^Yield for C_4+_.

^f^Selectivity for C_1_–C_4_.

In this investigation, we report the preparation of iron-based catalysts using the Organic Combustion Method (OCM) and determined their catalytic performance for the direct and efficient conversion of CO_2_ to jet fuel range hydrocarbons. In brief, the Fe–Mn–K catalyst shows a CO_2_ conversion of 38.2% and selectivity to C_8_–C_16_ hydrocarbons of 47.8% with a correspondingly low selectivity for CH_4_ and CO. In addition, the process also shows a high molar production ratio of olefin-to-paraffin for C_2_–C_4_ hydrocarbons.

## Results

The rising concerns over climate change and the stringent environmental regulations to deplete the utilization of fossil-derived fuels have generated great opportunities—and major scientific challenges—on the transformation of CO_2_ into sustainable, synthetic hydrocarbons fuels, particularly in the synthesis of renewable aviation fuels. At the heart of any progress in this area, the all-important conversion process is closely related to the development of advanced catalysts of high performance for the CO_2_ hydrogenation reaction. Therefore, the utilization of novel methods of catalyst preparation represents an important strategy to produce advanced catalytic formulations having high-performance levels. Among the catalyst synthesis methods, the so-called OCM is recognized as an energy-efficient and economically viable approach for the one-pot synthesis of a variety of nanostructured solid catalysts. In this method, the utilization of an organic fuel, having also a cation-complexation character, to yield a homogenous redox solution of the different metal precursors is highly advantageous. In addition, a relatively moderate self-sustaining exothermic reaction of the redox gel may be beneficial to produce the necessary nanostructured catalysts with an efficient promoter effect due to the well-controlled aqueous chemistry of the preparation route and ensuing combustion conditions.

### The performance of Fe–Mn–K catalysts for the hydrogenation of CO_2_

**Fig. 1:**
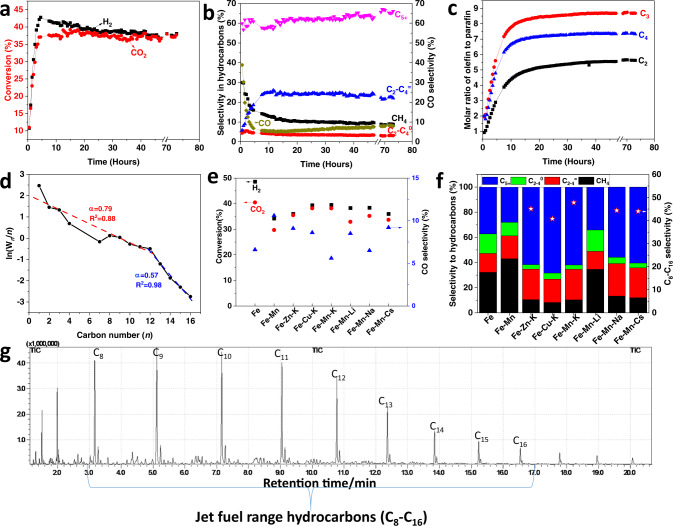
Catalyst performance for the hydrogenation of CO_2_ using a Fe–Mn–K catalyst. **a** % conversion of CO_2_ and H_2_ as a function of reaction time for the hydrogenation of CO_2._ **b** Selectivity of various hydrocarbon products with reaction time for the hydrogenation of CO_2._ **c** Molar ratio of olefin-to-paraffin for the C_2_–C_4_ range with reaction time for the hydrogenation of CO_2._ **d** ASF plot and *α* values at reaction time of 20 h. **e** Conversion and CO selectivity of CO_2_ hydrogenation for a reaction time of 20 h over different catalysts. **f** Products selectivities of CO_2_ hydrogenation for a reaction time of 20 h over different catalysts. **g** GC-MS total ion chromatogram (TIC) of the hydrocarbon fuel from the hydrogenation of CO_2_ on a Fe–Mn–K catalyst. The jet fuel range hydrocarbons (C_8_ to C_16_) are shown.

In terms of the conversion of CO_2_ and H_2_ to hydrocarbons and CO, the product selectivities, the Anderson–Schulz–Flory (ASF) product distribution, together with the molar ratio of olefin-to-paraffin ratio for C_2_–C_4_ from the hydrogenation of CO_2_ using a Fe–Mn–K catalysts, is shown in Fig. 1; specifically, both the conversions and selectivities for CO_2_ hydrogenation are shown for a reaction time of 20 h over a variety of Fe-based catalysts. The GC-MS spectrum of the collected liquid products from the hydrogenation of CO_2_ on the catalyst Fe–Mn–K is also shown in Fig. 1g. The GC-FID chromatogram of the gaseous hydrocarbon products from CO_2_ hydrogenation for a reaction time of 20 h, using an example Fe–Mn–K catalyst, is presented in Supplementary Fig. 1.

The data in Fig. 1 illustrate that the Fe–Mn–K catalyst exhibits high activity for the CO_2_ hydrogenation. The CO_2_ and H_2_ conversion increased rapidly with onset of reaction time in the first 5 h, reaching a stable value of around 40%. The methane selectivity decreased from 30 to 10% from the beginning of the reaction until 20 h, and decreased by a small amount after a further 20 h. In contrast, the light olefin selectivity (C_2_–C_4_ olefins) increased to an apparent limiting value of 25% at a reaction time of 10 h and above. The liquid product (C_5_^+^) selectivity was stable at around 60% and showed a small increase with reaction time. Similarly with FTS, the hydrocarbon products from CO_2_ hydrogenation on Fe–Mn–K generally follow the ASF distribution. Figure 1d shows a double ASF product distribution^53^, whose chain growth probabilities (*α*_*i*_) is 0.79 for *α*_1_ within the C_1_–C_12_ carbon range and *α*_2_ is 0.57 for C_12+_(i.e., heavy hydrocarbons). A high chain growth probability (*α*_1_) means a low methane selectivity whilst the chain growth decreases when the carbon number is above 12, indicating lower selectivity for higher (heavier) hydrocarbons.

Compared with the literature results in Table 1, the prepared Fe–Mn–K catalyst showed higher liquid products (C_5+_) yield, with the catalyst presenting both high CO_2_ conversion and high C_5+_ selectivity.

Interestingly, the methane selectivity decreased dramatically at the beginning of the reaction due to the main reaction being CO_2_ methanation over the catalyst active sites (χ-Fe_5_C_2_). They produced a high pressure of water and unconverted CO_2_ which, importantly, can then oxidize χ-Fe_5_C_2_ to Fe_3_O_4_. The CO produced via the RWGS reaction on Fe_3_O_4_ active site reacts with H_2_ (Fisher-Tropsch synthesis (FTS)), and the CO_2_ conversion increased rapidly (Fig. 1a). The product selectivity was then stable after a reaction time of 10 h.

The catalyst also showed a high selectivity for the production of light olefins versus alkanes, with molar ratios of ethylene -to -ethane, propylene-to-propane, and butane-to-butane of 5, 8.5, and 7, respectively (Fig. 1c). The GC-FID chromatograms (Supplementary Fig. 1) also show that olefins were the dominant products in the C_2_–C_4_ hydrocarbon fractions. Overall, the Fe–Mn–K catalyst showed high activity for CO_2_ hydrogenation reactions and high liquid hydrocarbon, and light olefin product selectivity.

The GC-MS spectrum of the collected liquid products clearly demonstrates that the Fe–Mn–K catalyst has high selectivity for jet fuel range hydrocarbons as liquid products; the total jet fuel range hydrocarbon selectivity is up to 47.8% among all hydrocarbons. The corresponding yield of jet fuel range hydrocarbons was 17.2% with a CO_2_ conversion of 38.2%.

### Catalyst characterisation

**Fig. 2:**
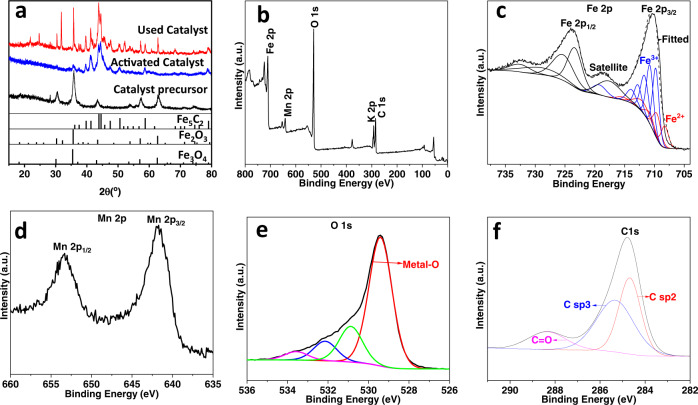
XRD and XPS spectra of the Fe–Mn–K catalyst. **a** Powder XRD spectra of the catalyst precursor and both the activated and the used catalyst. The corresponding JCPDS numbers are, for Fe_2_O_3_: 00-020-0508; χ-Fe_5_C_2_: 00-024-0081; Fe_3_O_4_: 03-065-3107; **b** XPS survey spectrum of the Fe–Mn–K catalyst; **c** High-resolution XPS spectra in the region of the Fe 2p peak on the Fe–Mn–K catalyst; **d** XPS spectra of the Mn 2p on the Fe–Mn–K catalyst; **e** XPS spectra of the O 1s on the Fe–Mn-K catalyst; **f** XPS spectra of the C 1s on the Fe–Mn–K catalyst.

The catalyst precursor was firstly activated in situ with syngas (H_2_:CO = 2:1) prior to catalytic performance evaluation, with a GHSV (gas hourly space velocity) conditions of 1000 mL g^−1^ h^−1^ at atmospheric pressure, a temperature of 320 °C and for 24 h duration. The powder X-ray diffraction (XRD) spectra of the catalyst precursor, together with the activated and used Fe–Mn–K catalysts are shown in Fig. 2 (a).

It can be seen from Fig. 2a, that all the reflections from the catalyst precursor can be assigned to Fe_3_O_4_ but in contrast, and importantly, all reflections from the activated catalyst can be assigned to χ-Fe_5_C_2_, which indicated that the Fe_3_O_4_ is fully carburized to χ-Fe_5_C_2_ under the treatment with syngas (CO/H_2_ ratio of 1:2). The reflections in the powder diffractogram from the used catalyst phase were considerably more complex, consisting of mixtures of Fe_3_O_4_, Fe_2_O_3_, and χ-Fe_5_C_2_. Importantly, χ-Fe_5_C_2_ is widely acknowledged to be the active catalytic species in the in situ hydrogenation of CO and/or CO_2_ and this iron carbide phase plays a crucial role in the subsequent C–C chain growth reactions^54–57^.

We have observed that the catalyst precursor is almost fully converted to χ-Fe_5_C_2_ during the in situ activation process, whilst Fe_3_O_4_ is partially regenerated during the first hours of the catalytic reaction, hence explaining the increases in H_2_ and CO_2_ conversion during this period. This finding is perfectly consistent with the recognized “Tandem mechanism” in which these two catalytically active phases (χ-Fe_5_C_2_ and Fe_3_O_4_) are responsible for the conversion of CO_2_ and H_2_ to syngas and for the subsequent C–C chain growth step to produce jet fuel^44,58–60^.

The formation of Fe_2_O_3_ in the used catalyst probably arises from the oxidation of Fe_3_O_4_ by CO_2_ and/or water during the reaction, while the Fe_2_O_3_ was reduced to Fe_3_O_4_ in the presence of H_2_ (showed in Supplementary Fig. 24).

Crystallite sizes were calculated using the Scherrer equation for the Fe-based catalysts precursor and these are listed in Table 2. The crystallite size for the Fe–Mn–K catalyst is typically around 14 nm, which is reflected in the observed broad reflections in the XRD spectrum of the catalyst precursor (as shown in Fig. 2).

**Table 2:** Crystallite sizes of prepared catalysts (with different transition metal promoters) with the citric acid method.

Catalyst	2θ	FWHM	d-spacing (nm)	Crystallite size (nm)
Fe–Zn–K	35.73	0.13	0.25	64
Fe–Cu–K	35.91	0.11	0.25	74
Fe–Mn–K	35.75	0.60	0.25	14

Surface elemental compositions and the oxidation states of the metals were analyzed using XPS in the region 0–800 eV. The survey spectrum (Fig. 2b) clearly indicates that the sample contains Fe, Mn, K, and O. Figure 2c shows the XPS spectrum of the Fe 2p region, which can be fitted with two spin-orbit doublets corresponding to the Fe 2p_3/2_ and Fe 2p_1/2_ peaks with a binding energy gap of 13.7 eV and a shakeup satellite which is assigned to Fe^3+^, consistent with those for Fe_3_O_4_^61^. The measured molar ratio of Fe^2+^ : Fe^3+^ is 1:2.38, which approximates to the stoichiometry of Fe_3_O_4_ (the ratio of Fe^2+^:Fe^3+^ for Fe_3_O_4_ is 1:2). In Fig. 2d we show the Mn 2p XPS spectra, which displayed a spin-orbit doublet of Mn 2p3/2 and Mn 2p1/2 peaks with a binding energy gap of 11.6 eV can be assigned to Mn_2_O_3_. In addition, in Fig. 2e we show the O 1s, XPS spectra with a main peak at 529.4 eV, clearly originating from the presence of metal-O bonds.

The XPS spectra of the C 1s present (Fig. 2f) showed that around 40% of C sp2 at a characteristic binding energy peak of 284.7 eV; some 15% C=O at a binding energy peak of 288.4 eV; and finally, 45% C sp3 at binding energy peak of 285.3 eV. The C sp2 is due to the carbon residue due to the calcination of citric acid, and the peak of C=O and C sp3 can be attributed to the citric acid residues which have not fully decomposed.

Temperature-programmed oxidation (TPO) results of Fe–Mn–K catalyst precursor prepared with citric acid combustion method shown in Supplementary Fig. 24 revealed a small amount (about 3.5 wt%) of carbon residue in the after calcination at 350 °C. The presence of this small amount of carbon in the catalyst is reported to be beneficial for a higher olefin product selectivity. Thus, previous work^62,63^ reported that the surrounding carbonaceous matter could indeed facilitate the formation of iron carbides during activation, hence improving the higher liquid products selectivity.

**Fig. 3:**
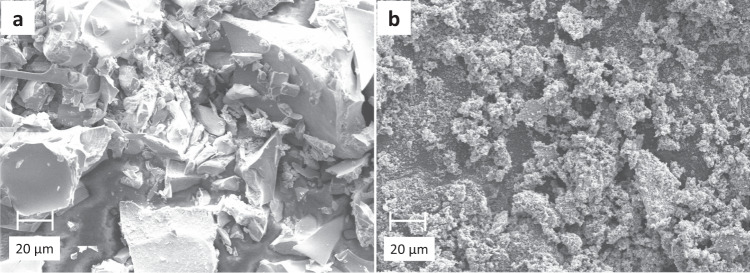
SEM images of Fe–Mn–K catalyst. **a** The Fe–Mn–K catalyst precursor; **b** the used Fe–Mn–K catalyst.

**Fig. 4:**
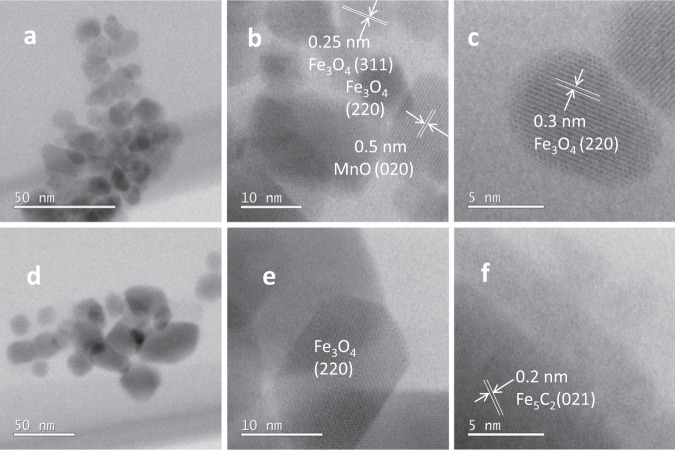
STEM-BF images of the Fe–Mn–K at different nanoscales. **a**–**c** The Fe–Mn–K catalyst precursor; **d**–**f** the used Fe–Mn–K catalyst.

Scanning electron microscopy (SEM) images of both the catalyst precursor and the used catalysts are shown in Fig. 3. The precursor consists of closely packed, regular particles (Fig. 3a). Obvious changes take place in the morphology of the catalyst after reaction (Fig. 3b). STEM-BF images of the catalyst precursor and used catalyst were also recorded as shown in Fig. 4.

From Fig. 4a–c it is evident that the catalyst precursor consists of nanoparticles with sizes of ~15 nm. Interestingly, there was no obvious change in catalyst particle size following the hydrogenation reaction (Fig. 4d). In the catalyst precursor (Fig. 4b, c), the measured lattice spacings of 0.25 and 0.3 nm correspond to the (311) and (220) planes of Fe_3_O_4_, respectively. In addition to the Fe_3_O_4_ phase (Fig. 4e), a χ-Fe_5_C_2_ phase was also observed in the used catalysts (Fig. 4f). This has been proposed as the source of the catalytically active sites for CO and/or CO_2_ hydrogenation to hydrocarbons, as previously reported^54–57^.

### The effects of transition metal promoters

Manganese compounds are widely utilised as promoters in iron-based catalysts for FTS where the addition of Mn typically improves activity, increases the surface basicity and enhances the carburization of the catalyst^64,65^. In addition to Mn, Zn^66,67^ and Cu^43,68^ have also been used as promoters for Fe-based catalysts for FTS. Thus, Fe–Zn–K and Fe–Cu–K catalysts were also prepared by the same method as the Fe–Mn–K catalyst. The catalytic performance for CO_2_ hydrogenation using these different catalysts are shown in Fig. 1e, f.

The data in Fig. 1e show that even the iron-catalyst without promoter showed high activity for CO_2_ hydrogenation, as reflected in the high conversion values. However, from Fig. 1f we can see the methane selectivity was very high, and reached 32.2% while the liquid product selectivity was very low.

In contrast, the promoter-added catalysts, Fe–Zn–K, Fe–Cu–K, and Fe–Mn–K showed high CO_2_ conversion and high jet fuel range hydrocarbon selectivity. There was no significant difference between the performances of these three catalysts, but the Fe–Mn–K catalyst showed slightly better selectivity for jet fuels synthesis (47.8%) than catalysts of Fe–Cu–K (40.8%) and Fe–Zn–K (45.1%).

### Effects of base-metal promoters

The impact of potassium (K) compounds on the performance of Fe-based catalysts for FTS has been studied extensively^69,70^. K is known to promote the formation of longer-chain hydrocarbons, the carburization of surface Fe, and the suppression of CH_4_ formation, all of which are advantageous for liquid hydrocarbon synthesis. In addition to K, various base metal compounds of Na have also been used as promoters for FTS catalysts^71,72^. Therefore, these base metals have also been tested as promoters for the catalytic hydrogenation of CO_2_ and their various catalytic performance are summarised in Fig. 1e, f.

It is clear from the data in Fig. 1e, that Na, K, and Cs-based promoters all show both high activity for CO_2_ hydrogenation and high selectivity for hydrocarbons in the jet fuel range. However, the Li promoted catalyst showed high selectivity for methane formation but not for long-chain hydrocarbons (Fig. 1f). There are no large differences in the catalytic performance between the catalysts Fe–Mn–K, Fe–Mn–Na, and Fe–Mn–Cs. However, the Fe–Mn–K catalyst showed higher C_8_–C_16_ selectivity (47.8%) than Fe–Mn–Na (44.4%) or Fe–Mn–Cs (44.0%).

Crystallite sizes calculated using the Scherrer equation for the different base metal promoted catalysts precursor are listed in Table 3. The catalysts have different crystallite sizes ranging from 19 to 32 nm.

**Table 3:** Crystallite size of the various prepared catalysts (different base metal) with citric acid method.

Catalyst	2θ	FWHM	d-spacing (nm)	Crystallite size (nm)
Fe–Mn–Li	36.07	0.45	0.25	19
Fe–Mn–Na	35.98	0.26	0.25	32
Fe–Mn–Cs	36.03	0.30	0.25	28

### Effects of organic fuel compounds

Recently, the Organic-Combustion Method (OCM), also known as the Solution Combustion Method, has been developed to prepare highly active metal catalysts for a variety of processes^73^. In order to investigate the fundamental, underlying efforts of organic fuel compounds, the catalyst prepared without fuel also been synthesised. It is clear that the catalyst prepared without fuel showed lower catalytic activity (CO_2_ conversion of 28.6%) compared with the catalyst prepared with citric acid (CO_2_ conversion of 38.2%). In addition to using citric acid as a fuel in the OCM catalyst preparation, we have also investigated other organic chemicals as potential fuel sources. A series of catalysts of the Fe–Mn–K type were therefore prepared with different organic compounds in the catalyst preparation by the combustion route and their catalytic performances for the hydrogenation of CO_2_ are listed in Table 4.

**Table 4:** Effect of organic (fuel) compounds on CO_2_ hydrogenation catalyst performance. All reactions were conducted at 1 MPa, 300 °C, GHSV 2400 ml g^−1^ h^−1^ and H_2_:CO_2_ (3:1) on Fe–Mn–K catalyst.

Organic compound applied in catalysts preparation	Conversion (%)		CO selectivity (%)	Selectivity to hydrocarbons (%)				
	H_2_	CO_2_		CH_4_	C_2–4_=	C_2–4_0	C_5+_	C_8–16_
No fuel	27.0	28.6	6.5	14.1	26.0	5.2	54.7	–
Urea	34.4	35.0	5.8	14.3	27.6	4.6	53.5	38.3
Tannic acid	39.2	38.8	5.0	16.2	26.4	4.8	52.6	34.5
EDTA	39.6	40.6	7.0	13.5	21.6	4.4	60.5	51.0
Citric acid	39.5	38.2	5.6	10.4	24.2	3.5	61.9	47.8
Oxalic acid	36.7	37.0	7.4	9.8	25.4	3.5	61.3	47.9
NTA	39.5	42.5	5.1	7.2	18.3	2.6	71.9	49.0
DTPA	42.0	44.0	6.2	9.6	19.0	3.4	68.0	53.3
Tartaric acid	40.1	41.8	4.6	7.9	18.9	2.7	70.4	50.2
HEDTA	42.3	41.5	4.9	9.6	20.2	3.1	67.0	47.0
Salicylic acid	37.8	37.3	7.2	12.6	22.0	3.8	61.5	49.5
Sugar	36.5	37.4	8.8	10.3	21.5	3.9	64.3	45.8
Flour powder	36.5	35.6	7.2	12.1	25.9	4.0	58.0	39.8

The molar ratio of K and Mn to Fe was 1:10. Data were obtained at a reaction time of 20 h. C_2–4_=: C2–C4 olefin, C_2–4_0: C2–C4 paraffin; C_5+_: liquid products; C_8–16_: Jet fuel range hydrocarbons.

It is clear, therefore, that compared to the catalyst prepared without an organic fuel, all the Fe–Mn-K catalysts prepared with organic compounds generally showed both higher CO_2_ conversion and higher jet fuel range hydrocarbon selectivity. The catalysts prepared with EDTA, citric acid, oxalic acid, NTA, DTPA, Tartaric acid, HEDTA, and salicylic acid also exhibited good catalytic performance for both CO_2_ conversion and jet fuel selectivity. In general, all these organic fuels could also act as chelating agents and hence facilitating the formation of nanostructured catalysts.

The XRD spectra of various catalysts are shown in Supplementary Fig. 4. The catalyst prepared without fuel showed characteristic reflections assigned to Fe_2_O_3_. However, most of the catalysts prepared with organic fuel compounds showed Fe_3_O_4_ as the dominant crystalline phase which clearly indicates that part of Fe^3+^ present in Fe_2_O_3_ was partially reduced to Fe^2+^ in Fe_3_O_4_ during the catalyst preparation stage. The catalyst prepared with oxalic acid showed XRD reflections corresponding to Fe_2_O_3_ instead of Fe_3_O_4_. This implies that under the conditions applied in this investigation, oxalic acid did not reduce the Fe_2_O_3_ to Fe_3_O_4_ consistent with its low reducing power compared to the other organic fuels.

The crystallite sizes of catalysts calculated from the Scherrer equation are listed in Table 5. Importantly, catalysts prepared with a range of different organic compounds showed smaller crystallite sizes than the catalyst prepared without fuel. We attribute these differences in crystallite sizes as the possible origins of the higher activity of catalysts prepared with organic fuels.

**Table 5:** Crystallite size of catalysts prepared with different organic compounds.

Organic compound applied in catalysts preparation	2θ	FWHM	d-spacing (nm)	Crystallite size (nm)
–	33.42	0.13	0.27	63
Urea	35.83	0.15	0.25	56
Tannic acid	35.85	0.60	0.25	14
EDTA	35.74	0.67	0.25	12
Glycine	35.46	0.30	0.25	28
Citric acid	35.75	0.60	0.25	14
Oxalic acid	35.87	0.45	0.25	19
NTA	35.94	0.34	0.25	25
DTPA	35.96	0.67	0.25	12
Tartaric acid	35.78	0.30	0.25	28
HEDTA	35.95	0.37	0.25	22
Salicylic acid	35.69	1.20	0.25	7
Sugar	36.04	0.11	0.25	74
Flour powder	35.92	0.67	0.25	12

Compared to the co-precipitation method, widely applied in the preparation of Fe-based catalysts^49–52^, we show that the OCM is a particularly facile production process where, in addition to high yields and selectivity for jet fuels, additional advantages are savings in both energy and time^74^.

An optimal organic compound in our catalyst preparation should act both as a reducing agent and should react with nitrates non-violently, produce nontoxic gases and also act as an effective chelating agent for metal cations.

The catalysts prepared using organic fuels showed high activity as stable organic chelate compounds formed with metal cations are particularly suited to the formation of uniform, highly dispersed metal oxide catalysts via the combustion method.

The gaseous products from the organic compound and nitrate combustion reactions are N_2_, CO_2,_ and H_2_O. Using citric acid as an example, the stoichiometric reactions can be described as follows, according to the principle of propellant chemistry:

$$54\;{\mathrm{Fe}}\left( {{\mathrm{NO}}_3} \right)_3\,9{\mathrm{H}}_2{\mathrm{O}} + 46\;{\mathrm{C}}_6{\mathrm{H}}_8{\mathrm{O}}_7\,{\mathrm{H}}_2{\mathrm{O}} \\ \quad\to 18\;{\mathrm{Fe}}_3{\mathrm{O}}_4 + 81\;{\mathrm{N}}_2 + 276\;{\mathrm{CO}}_2 + 716\;{\mathrm{H}}_2{\mathrm{O}}$$

$$9\;{\mathrm{Mn}}\left( {{\mathrm{NO}}_3} \right)_2\,4{\mathrm{H}}_2{\mathrm{O}} + 5\;{\mathrm{C}}_6{\mathrm{H}}_8{\mathrm{O}}_7{\mathrm{H}}_2{\mathrm{O}} \\ \to 9\;{\mathrm{MnO}} + 9\;{\mathrm{N}}_2 + 30\;{\mathrm{CO}}_2 + 61\;{\mathrm{H}}_2{\mathrm{O}}$$

These combustion reactions are highly exothermic and lead to a rapid evolution of a large volume of gaseous products during the catalysts preparation process. This release of gas depletes the fuel combustion heat and hence limits the rapid temperature rise, thereby advantageously reducing any premature local partial sintering of the primary metals oxides particles. The gas evolution also results in limiting any extended crystal growth or inter particle contact, thereby contributing to smaller particle size catalysts^75^.

Although combustion was nominally carried out at 350 °C, the in situ flame temperature during combustion can be very high due to the combustion of gases produced during the decomposition of metal nitrates and the organic compounds. This high-temperature persists for a few minutes and disappears, producing a rapid quenching effect^76^. which is known to enhance the interaction between Fe_3_O_4_ and the promoter, further improving catalytic performance. It is interesting that most of redox gel when combusted at a calcining temperature of 350 °C, produces Fe_3_O_4_ without any apparent O_2_ participation from the atmosphere However, the main products are Fe_2_O_3_ when the calcination temperature increased to 500 °C, one presumes clearly as a consequence of the participation of atmospheric oxygen and/or the associated oxygens from the complexing ligands.

In general, the Fe–Mn-K catalysts synthesised with carboxylic acids and polycarboxylic acids as fuels showed superior catalytic performances than those prepared using urea and sugar (glucose) and the catalyst prepared without fuel. Our assertion is that this trend probably derives from two crucial roles (i.e., both a chelating agent and fuel) that these organic molecules play in the organic-combustion approach. The first role can enhance the homogeneity of the solution through the intimacy between the constituent metal (Fe, Mn, K) precursors, hence hindering their precipitation or aggregation during the gel formation, whilst the second (fuel) function can closely control the severity of the combustion reaction and hence the aggregation of the nanostructured catalysts. Obviously, this leads to changes in the crystallite sizes that show the Fe–Mn–K catalysts with particle sizes between 7 and 28 nm and usually prepared by carboxylic acid-type fuels are significantly more active and selective than the catalysts with larger crystallite sizes (i.e., 56–74 nm).

Finally, we have also examined commercial sugar and flour powders as possible fuels in the catalyst preparation process. Catalysts prepared with these fuels also showed high CO_2_ hydrogenation activity and jet fuel range hydrocarbon selectivity. The catalytic performance for CO_2_ hydrogenation of catalysts prepared with different fuels are shown in Supplementary Figs. 10–22.

### The reaction scheme

**Fig. 5:**
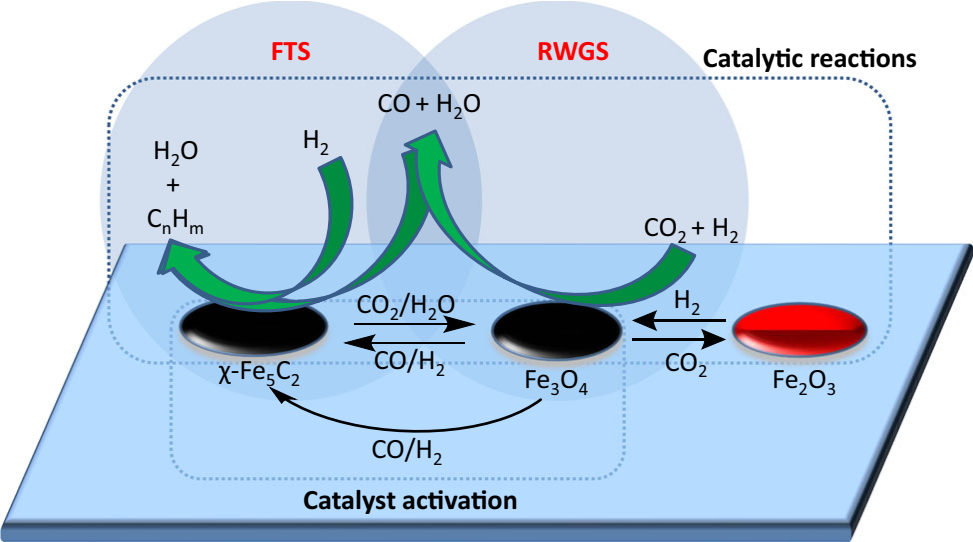
Reaction scheme for CO_2_ hydrogenation to jet fuel range hydrocarbons. The CO_2_ hydrogenation to jet fuel range hydrocarbons process through a Tandem Mechanism in which the Reverse-Water Gas Shift reaction (RWGS) and Fischer-Tropsch synthesis (FTS) reaction are catalysed by Fe_3_O_4_ and χ-Fe_5_C_2_ respectively.

Hägg carbide (χ-Fe_5_C_2_) is widely accepted to be the active catalytic species in the FTS and this iron carbide phase plays a crucial role in the C–C chain growth reactions^54–57^. The χ-Fe_5_C_2_ usually prepared by activation of hematite under syngas atmosphere at temperature of 200–450 °C, the whole activation process contains a three-step reduction process of Fe_2_O_3_ to iron (Fe_2_O_3_ → Fe_3_O_4_ → FeO → Fe) and then followed by carburisation of Fe to χ-Fe_5_C_2_^77^. In our experiments, the χ-Fe_5_C_2_ was reduced and carburized from Fe_3_O_4_, in the process of magnetite (Fe_3_O_4_) → wüstite (FeO) → iron metal (Fe) → Hägg carbide (χ-Fe_5_C_2_). Wei et al.^44^ have proposed a comprehensive reaction scheme where χFe_5_C_2_ is involved in the hydrogenation of CO_2_ to gasoline fuel range hydrocarbons, using a Na-Fe_3_O_4_/Zeolite multifunctional catalyst. We believe that a related, but slightly different, reaction scheme is operating here for the hydrogenation of CO_2_ to aviation jet fuel and this is illustrated schematically in Fig. 5.

In contrast to the report by Wei et al.^44^ who described catalysts prepared by a one-pot synthesised Na-Fe_3_O_4_ catalyst with zeolite, we have prepared catalysts using the direct OCM route which showed higher liquid products yield on CO_2_ hydrogenation. Thus the Fe_3_O_4_ catalyst precursor is fully carburized to χ-Fe_5_C_2_ during our catalyst activation (reduction) process, whilst Fe_3_O_4_ is partially regenerated from the oxidation of χ-Fe_5_C_2_ by CO_2_ /water in the first hours of the catalytic reaction. Jet fuel synthesis via CO_2_ hydrogenation initially takes place by the RWGS reaction (CO_2_ + H_2_ → CO + H_2_) on the catalytically active sites of Fe_3_O_4_, and subsequently by the FTS reactions (CO + H_2_ → C_n_H_m_ + H_2_O) on catalytically active sites on χ-Fe_5_C_2_^54–57^.

Using iron-based catalysts for FT synthesis a fast and reversible exchange of Fe_3_O_4_ to Fe_*x*_C_*y*_ carbides and vice versa can occur under appropriate reaction conditions. This relatively facile and reversible phase transformation makes possible the incorporation of carbon atoms from the carbide surface into the reaction products via Mars-van Krevelen mechanism as was determined by Gracia et al.^78^ through a computational study of the CO hydrogenation on an iron carbide surface. Remarkably, this Mars-van Krevelen-like mechanism on supported Fe catalysts rationalised the enhanced reactivity of highly dispersed iron carbide particles in the initiation of chain growth in F-T synthesis^79^.

As far as we know, there is not a single report in the scientific literature of the Mars-van Krevelen mechanism operating in the CO_2_ hydrogenation reaction on Fe catalysts. Obviously, this reaction is more challenging than conventional FT synthesis since the catalyst must have an excellent balance of active sites (phases) to catalyse—in tandem mode—the reverse-water gas shift reaction (or CO_2_ partial hydrogenation) and also the CO hydrogenation via the FT reaction to produce Jet Fuel. Our tandem mechanism through the participation of Fe_3_O_4_ and χ-Fe_5_C_2_ can easily rationalise the jet fuel formation and give a wider picture of the evolution of the gas, liquid and solid phases during the catalytic reaction. Further work is needed to gain further insight into the possible occurrence of Mars-van Krevelen-like mechanism in the FT stage through carbon isotopic labelling studies. In a flowing gas system these will clearly be experimentally—and financially (!)—challenging.

The carbide phase detected by powder-XRD diffraction was χ-Fe_5_C_2_ which plays a principal role in the formation of hydrocarbons via FT reaction^54–57^. According to the literature and our own results, the carburization process of Fe nanoparticles during the catalytic reaction forms the Fe carbide phase, which through a FT pathway favours the C–C condensation reactions to produce large hydrocarbons within the range of aviation fuel. In our experiments, the χ-Fe_5_C_2_ was formed during the catalyst activation/reduction process, in the beginning of the reaction what it is happening is mainly CO_2_ methanation reaction on χ-Fe_5_C_2_, the relatively high pressure of water can then oxidize on χ-Fe_5_C_2_ to Fe_3_O_4_, and the Fe_3_O_4_ was simultaneously carburized by CO^80^. In model experiments, Fe_2_O_3_ was produced from the oxidation of Fe_3_O_4_ by CO_2_/H_2_O, and Fe_2_O_3_ was steadily reduced to Fe_3_O_4_ by H_2_ in the reaction system (Supplementary Fig. 23). Thus Fe_3_O_4_, χ-Fe_5_C_2,_ and Fe_2_O_3_ co-exist during the reaction. Using Mn compounds as a promoter noticeably improved the catalyst FTS activity, increased the catalyst surface basicity and enhanced the carburization of the catalyst^64,65^. The addition of K compounds promoted the formation of longer-chain hydrocarbon molecules, the carburization of surface Fe, and the suppression of CH_4_ formation, which strongly favours liquid hydrocarbon synthesis^69,70^. We also find that the addition of both Mn and K as promoters improved the Fe-catalyst performance, directly converting CO_2_ into jet fuel range hydrocarbons with high efficiency.

### Renewable jet fuels and the circular economy

The Circular Economy (CE) is an attractive, holistic concept gradually and steadily positioning itself as an alternative and reliable alternative to the present, “Business-as-Usual”, unsustainable Linear Economy (LE) based on the “Take, make and dispose” paradigm^71^. Nowadays, researchers have risen to the challenge of climate change and advanced the concept of the so-called “CO_2_ Circular Economy”, which directly integrates CO_2_ capture from the air (Direct Air Capture, DAC) and converts CO_2_ into value-added products^81–84^. This CO_2_ Circular Economy is a valid and highly powerful alternative route to simply burying huge volumes of captured CO_2_ underground and one in which future generations will surely expect us to have formed a major aspect of sustainable CO_2_ management.

Renewable jet fuels offer considerable potential in the worldwide drive for a future Sustainable Circular Economy Future for the aviation industry. The vision centres on CO_2_ conversion as an integral part of carbon recycling. The advances reported here offer a route out of the current, worldwide LE for jet fuels, based on the (present) Production-Consumption- Disposal/Emission structure, where the valuable natural resource, crude oil, is extracted, shipped across oceans, transformed into jet fuel and then combusted, with the combustion product either emitted into the atmosphere, or trapped and buried underground (through Carbon Capture and Storage). On the other hand, the CE approach is based on fundamentally—different Production—Consumption—Recycling/Recovery structure or Carbon Capture and Utilization, where, in this case, CO_2_ is indeed recognized as a powerful “Resource” to be recirculated using renewable energy to yield carbon-neutral jet aviation fuel.

Obviously, our advance can contribute significantly to more sustainable fuel production process if we input renewable energy into the chain for transforming CO_2_ into aviation jet fuel as an additional driving force for the inevitable and urgently required transition toward a circular fuel economy centred on renewable CO_2_ utilization.

Within a Jet Fuel CO_2_ Circular Economy, the “Goods” (here the Jet Fuel) are continually reprocessed in a closed environment, which saves the natural fossil resources and preserves the environment, whilst also, of course, creating significant numbers of new jobs, new economies and new markets.

**Fig. 6:**
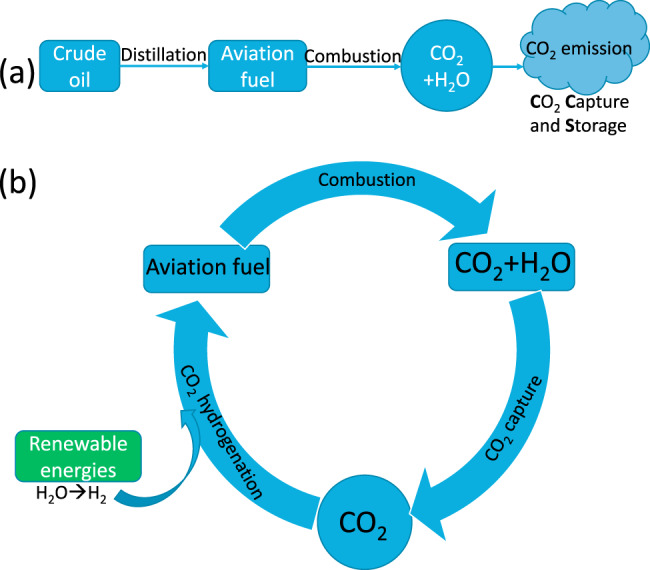
llustrating the differences between (**a**) an Aviation Jet Fuel Linear Economy and (**b**) an Aviation Jet Fuel Circular Economy.

In Fig. 6, we attempt to show a comparison of the Aviation Jet Fuel Linear Economy and the Aviation Jet Fuel Circular Economy. For the latter, Green H_2_ is derived from renewable energy, and CO_2_ is directly converted to Aviation Jet Fuel using our novel catalysts with CO_2_ captured from the atmosphere (“Air Capture”). Note the fundamental difference between the Jet Fuel Linear Economy, as compared to the CO_2_ to Jet Fuel Circular Economy, where the entire latter process is a closed loop and hence a CO_2_ neutral process. This CO_2_ Circular Economy for aviation can surely empower worldwide momentum toward not only major economic development for countries but also achieving the UN’s sustainable development goals.

## Discussion

A series of Fe-based catalysts were prepared by the OCM for the conversion of carbon dioxide into jet fuel range hydrocarbons. This synthetic process can be used to produce homogeneous, ultrafine and high-purity crystalline metal oxide powder catalysts. The as-prepared catalysts, following activation, showed high carbon dioxide hydrogenation activity and high jet fuel range selectivity as a consequence of the small (ca. 15 nm) nanoparticle size and the presence of two catalytically active Fe phases that operate in tandem. The first phase corresponds to Fe_3_O_4_ which catalyses the conversion of carbon dioxide to CO via the RWGS reaction whilst the second active Fe phase (χ-Fe_5_C_2_) catalyses the hydrogenation of CO through the Fischer-Tropsch process.

This catalytic process provides an attractive route not only to mitigate carbon dioxide emissions but also to produce renewable and sustainable jet fuel. The recycling of carbon dioxide as a carbon source for both fuels and high-value chemicals offers considerable potential for both the aviation and petrochemical industries. It also represents a significant social advance; thus, instead of consuming fossil crude oil, jet aviation fuels and petrochemical starting compounds are produced from a valuable and renewable raw material, namely, carbon dioxide. These advances highlight carbon dioxide recycling and resource conservation as an important, pivotal aspect of greenhouse gas management and sustainable development. This, then, is the vision for the route to achieving net-zero carbon emissions from aviation; a fulcrum of a future global zero-carbon aviation sector.

## Methods

### Catalyst preparation

Catalysts were prepared by the OCM method; citric acid was used as the organic compound. Typically, a Fe–Mn–K catalyst was prepared from citric acid monohydrate (99%, Sigma-Aldrich), Iron (III) Nitrate nonahydrate (98%, Sigma-Aldrich), Manganese(II) nitrate tetrahydrate (97%, Sigma-Aldrich) and potassium nitrate (99%, Sigma-Aldrich), in a molar ratio of citric acid: (Fe(or Co) + Mn + K) = 2, and a weight ratio of (Fe- and Mn- and K-precursors + citric acid)/water = 2:1. This initial mixture was stirred to form a homogeneous aqueous solution and heated at 50 °C for 1–2 h to obtain a citric acid-based slurry. This paste was then ignited at 350 °C in the air for 4 h to produce a carbon-free powder.

Catalyst samples with different first-row transition metal (Mn, Cu, Zn) promoters were also prepared using the same method; Fe–Cu–K and Fe–Zn–K catalysts were prepared using copper (II) nitrate trihydrate (99–104%, Sigma-Aldrich), and zinc nitrate hexahydrate (98%, Sigma-Aldrich) as transition metal precursors respectively. Similarly, catalysts with different Fe–Mn–Li, Fe–Mn–Na, and Fe–Mn–Cs base metal promoters were prepared using lithium carbonate (99%, Sigma-Aldrich), sodium carbonate (99.6%, Acros Organics) and cesium carbonate (99%, Sigma-Aldrich), respectively. Typically, the molar ratio of Fe: transit metal: base metal used was 10:1:1.

Fe–Mn-K catalysts were also prepared using other organic compounds other than citric acid, specifically; urea (Bio-Reagent, Sigma-Aldrich), tannic acid (ACS reagent, Sigma-Aldrich), Ethylenediamine Tetraacetic Acid (EDTA, 99.5%, Fisher Scientific), oxalic acid (99.0%, Sigma-Aldrich), Nitrilotriacetic acid (NTA, 99%, Sigma-Aldrich), Diethylenetriaminepentaacetic acid (DTPA, 98%, Sigma-Aldrich), tartaric acid (99.5%, Sigma-Aldrich), N-(2-Hydroxyethyl) ethylenediamine-N,N’,N’-triacetic acid (HEDTA,98%, Sigma-Aldrich), salicylic acid (99.0%, Sigma-Aldrich). In all discussions catalysts were prepared with citric acid as the organic compound unless otherwise stated.

### Catalysts performance evaluation

CO_2_ hydrogenation experiments were carried out in a stainless steel fixed bed reactor with an inner diameter of 1.0 cm (Zhixiang Blue Evaluation Equipment Technology) with a typical 2.0 g catalyst load. Prior to the reaction, the catalyst was in situ reduced with syngas (H_2_:CO = 2:1) at atmospheric pressure, with a GHSV (gas hourly space velocity) of 1000 mL g^−1^ h^−1^, at 320 °C for 24 h. Following reactor cooling to below 50 °C, a mixture of gas with an H_2_/CO_2_ ratio of 3 and N_2_ (as an internal standard gas) was introduced at a gas flow of 40 mL min^−1^ (GSVH = 2400 mL g^−1^ h^−1^). The reactor was then heated at a rate of 2 °C/min to 300 °C. The reaction pressure was fixed at 10 bar (1 MPa) using a back pressure regulator. The effluent gaseous products were analysed using an online Gas Chromatograph (Perkin Elmer Clarus 580 GC) with flame ionization (FID) and thermal conductivity detectors (TCD), and the collected liquid products were analysed by Gas Chromatograph Mass Spectrometry (SHIMADZU GCMS-QP2010 SE).

The CO_2_ and H_2_ conversion and product selectivity’s were calculated from the following relationships:

$${\mathrm{CO}}_2\;{\mathrm{conversion}} = \frac{{{\mathrm{CO}}_{2,\;{\mathrm{inlet}}} - \frac{{{\mathrm{N}}_{2\;inlet}}}{{{\mathrm{N}}_{2\;outlet}}} \times {\mathrm{CO}}_{2,\;{\mathrm{outlet}}}}}{{{\mathrm{CO}}_{2,\;{\mathrm{inlet}}}}} \times 100\%$$

$${\mathrm{H}}_2\;{\mathrm{conversion}} = \frac{{{\mathrm{H}}_{2\;inlet} - \frac{{{\mathrm{N}}_{2\;inlet}}}{{{\mathrm{N}}_{2\;outlet}}} \times {\mathrm{H}}_{2\;outlet}}}{{{\mathrm{H}}_{2\;inlet}}} \times 100\%$$

$${\mathrm{CO}}\;{\mathrm{yield}} = \frac{{\frac{{{\mathrm{N}}_{2\;inlet}}}{{{\mathrm{N}}_{2\;outlet}}} \times {\mathrm{CO}}_{{\mathrm{outlet}}}}}{{{\mathrm{CO}}_{2,\;{\mathrm{inlet}}}}} \times 100\%$$

$${\mathrm{CO}}\;{\mathrm{selectivity}} = \frac{{{\mathrm{CO}}\;{\mathrm{yield}}}}{{{\mathrm{CO}}_2\;{\mathrm{conversion}}}} \times 100\%$$

$${\mathrm{C}}_{\mathrm{n}}{\mathrm{H}}_{\mathrm{m}}\;{\mathrm{yield}} = \frac{{{\mathrm{n}} \times \frac{{{\mathrm{N}}_{2\;inlet}}}{{{\mathrm{N}}_{2\;outlet}}} \times {\mathrm{C}}_{\mathrm{n}}{\mathrm{H}}_{\mathrm{m}}{\mathrm{outlet}}}}{{{\mathrm{CO}}_{2,\;{\mathrm{inlet}}}}} \times 100\% \;\left( {{\mathrm{n}} = 1,2,3,4} \right)$$

$${\mathrm{C}}_{5 + }\;{\mathrm{yield}} = \left({\mathrm{CO}}_2\;{\mathrm{conversion}} - {\mathrm{CO}}\;{\mathrm{yield}} - \mathop {\sum}\limits_{n = 1}^4 {{\mathrm{C}}_{\mathrm{n}}{\mathrm{H}}_{\mathrm{m}}\;{\mathrm{yield}}} \right) \times 100\%$$

$${\mathrm{selectivity}}\;{\mathrm{in}}\;{\mathrm{hydrocarbons}} = \frac{{{\mathrm{C}}_{\mathrm{n}}{\mathrm{H}}_{\mathrm{m}}\;{\mathrm{yield}}}}{{{\mathrm{CO}}_2\;{\mathrm{conversion}} \times \left( {1 - {\mathrm{CO}}\;{\mathrm{selectivity}}} \right)}} \times 100\%$$

The selectivity of oxygenates (mainly alcohols) was not further considered in this study as it was below 1.0%.

### Catalyst characterization

Powder XRD analyses of catalysts used a Cu Kα (0.15418 nm) X-ray source (25 kV, 40 mA) in a Bruker D8 Advance diffractometer. Diffraction patterns were recorded over a 10–80° 2θ angular range using a step size of 0.02°.

X-ray photoelectron spectroscopy (XPS) of samples was performed using a Thermo Fisher Scientific Nexsa spectrometer. Samples were analysed using a micro-focused monochromatic Al X-ray source (72 W) over an area of ~400 × 200 µm^2^. Data were recorded at pass energies of 150 eV for survey scans and 40 eV for high-resolution scans with 1 and 0.1 eV step sizes respectively. Charge neutralisation was achieved using a combination of low energy electrons and argon ions. The resulting spectra were analyzed using Casa XPS peak fitting software and sample charging corrected using the C 1s signal at 284.8 eV as a reference.

Thermogravimetric analysis (TGA) was used to characterise the resulting carbon depositions in our catalyst samples. A TPO was carried out to determine the thermal stability of the produced carbons. The sample was heated from room temperature to 1000 °C at a heating rate of 10 °C/min under an air atmosphere with a flow rate of 100 ml/min.

The catalyst morphology was characterised using scanning electron microscopy (SEM JEOL 840F) at an accelerating voltage of 6.0 kV.

High-resolution scanning transmission electron microscopy (STEM) annular dark field (ADF) and bright field (BF) images were obtained using a probe corrected JEOL ARM200F at the David Cockayne Centre for Electron Microscopy operated at 200 kV with ADF inner and outer detector angles of 28–104.36 mrad, respectively, a 13.20 mrad BF outer angle, and a 14 mrad convergence semi-angle.

### Reporting summary

Further information on research design is available in the Nature Research Reporting Summary linked to this article.

## Supplementary information

Supplementary Information

Peer Review File

Reporting summary

## Data Availability

The authors declare that the main data supporting the findings of this study are contained within the paper and its associated Supplementary Information. All other relevant data are available from the corresponding author upon reasonable request.
