# Supplementary material for: Transforming carbon dioxide into jet fuel using an organic combustion-synthesized Fe-Mn-K catalyst
Source: Nat Commun. 2020 Dec 22;11:6395. doi: 10.1038/s41467-020-20214-z (PMC7755904; doi:10.1038/s41467-020-20214-z)
Supplement: Supplementary file 1 — Supplementary Information [file 41467_2020_20214_MOESM1_ESM.pdf]

Supplementary Information

Transforming Carbon Dioxide into Jet Fuel using an Organic Combustion-Synthesized Fe-Mn-K Catalyst

Benzhen Yao [1], Tiancun Xiao [1 *], Ofentse A. Makgae [2], Xiangyu Jie [1,3], Sergio Gonzalez-Cortes [1], Shaoliang Guan [4,5], Angus I. Kirkland [2, 6], Jonathan R. Dilworth [1], Hamid A. Al-Megren [7], Saeed M. Alshihri [7], Peter Dobson [8], Gari P. Owen [9], John M. Thomas [10], Peter P. Edwards [1**]

[1] KACST-Oxford Centre of Excellence in Petrochemicals, Inorganic Chemistry Laboratory, University of Oxford, South Parks Road, Oxford, OX1 3QR, UK.

[2] Department of Materials, University of Oxford, Parks Roads, Oxford OX1 3PH, UK.

[3] Merton College, University of Oxford, Merton Street, Oxford, OX1 4JD

[4] Cardiff Catalysis Institute, School of Chemistry, Cardiff University, Cardiff, UK.

[5] Harwell-XPS – The EPSRC National Facility for Photoelectron Spectroscopy, Research Complex at Harwell (RCaH), Didcot, Oxon, OX11 0FA, UK.

[6] Electron Physical Sciences Imaging Centre, Diamond Lightsource Ltd., OX11 0DE, U.K.

[7] Materials Division, King Abdulaziz City for Science and Technology, Riyadh 11442, Kingdom of Saudi Arabia

[8] The Queen's College, University of Oxford, Oxford OX1 4AW, UK

[9] Annwvyn Solutions, 76 Rochester Avenue, Bromley, Kent BR1 3DW, UK.

[10] Department of Materials Science and Metallurgy, University of Cambridge, 27 Charles Babbage Road, Cambridge, CB3 0FS, UK

Corresponding author:

Dr. Tiancun Xiao, E-mail: xiao.tiancun@chem.ox.ac.uk; ORCID: 0000-0002-2278-3363

Professor Peter P. Edwards, E-mail: peter.edwards@chem.ox.ac.uk; ORCID: 0000-0002-1379-9400

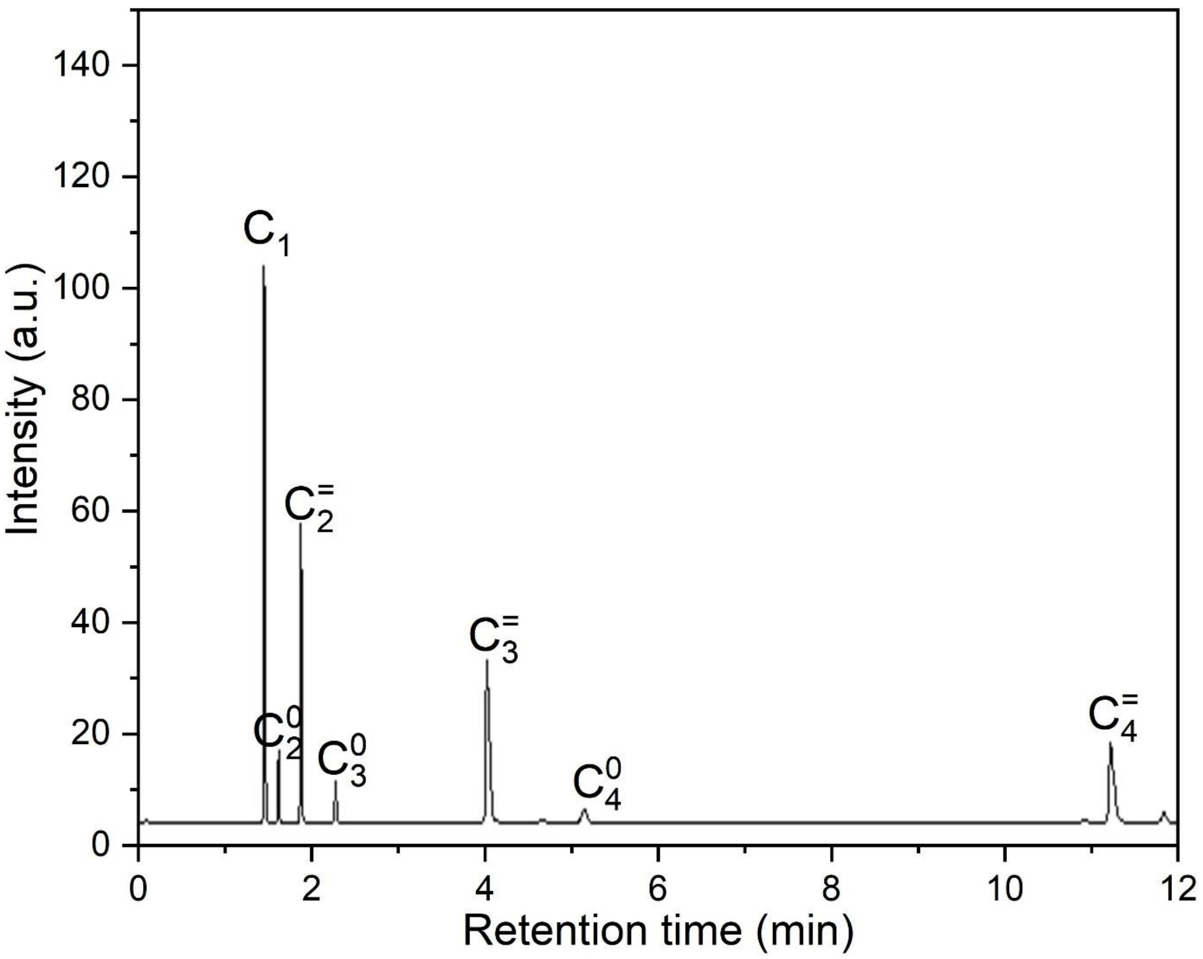

Supplementary Figure 1 GC-FID chromatograms of the gaseous hydrocarbon products from the hydrogenation of CO_2, taken at a reaction time of 20 hours.

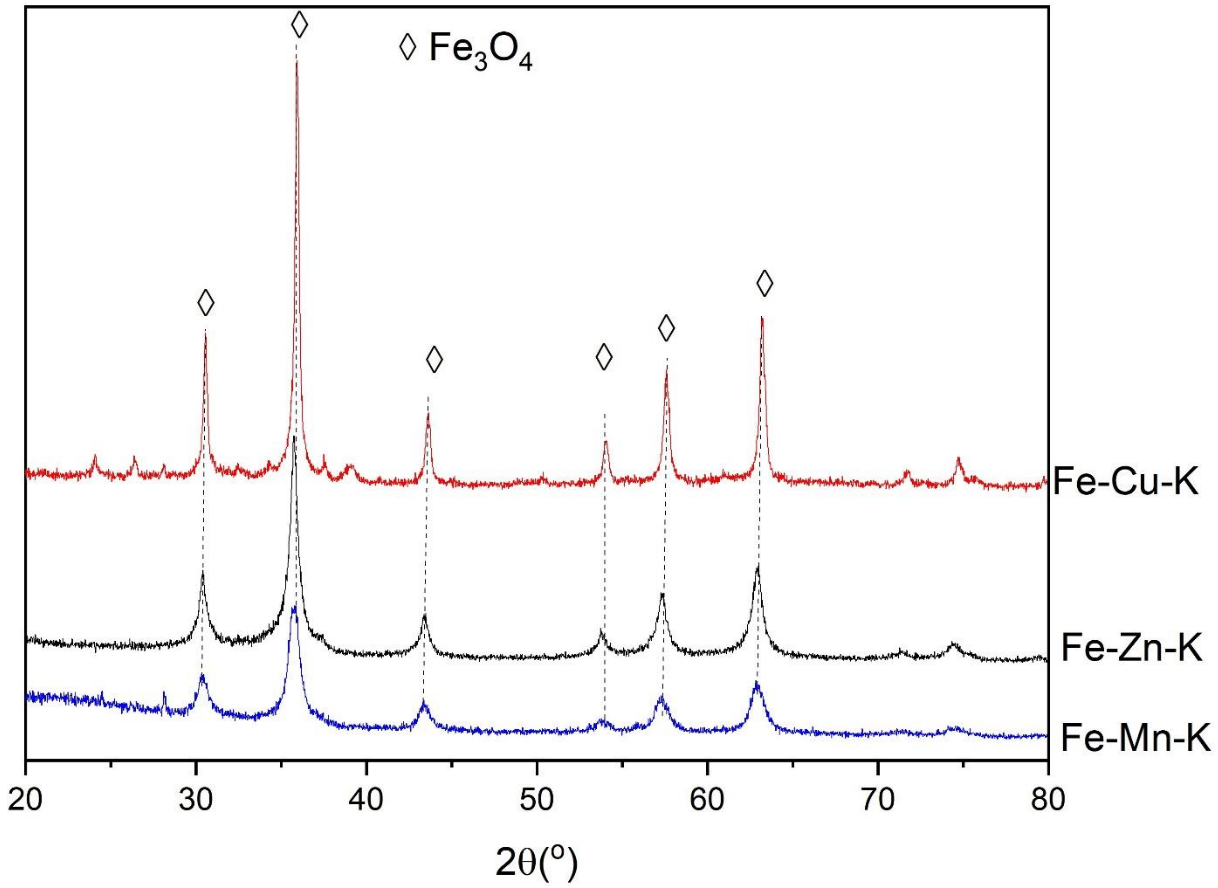

Supplementary Figure 2 XRD spectrum of catalysts (different transit metal) prepared with citric acid method

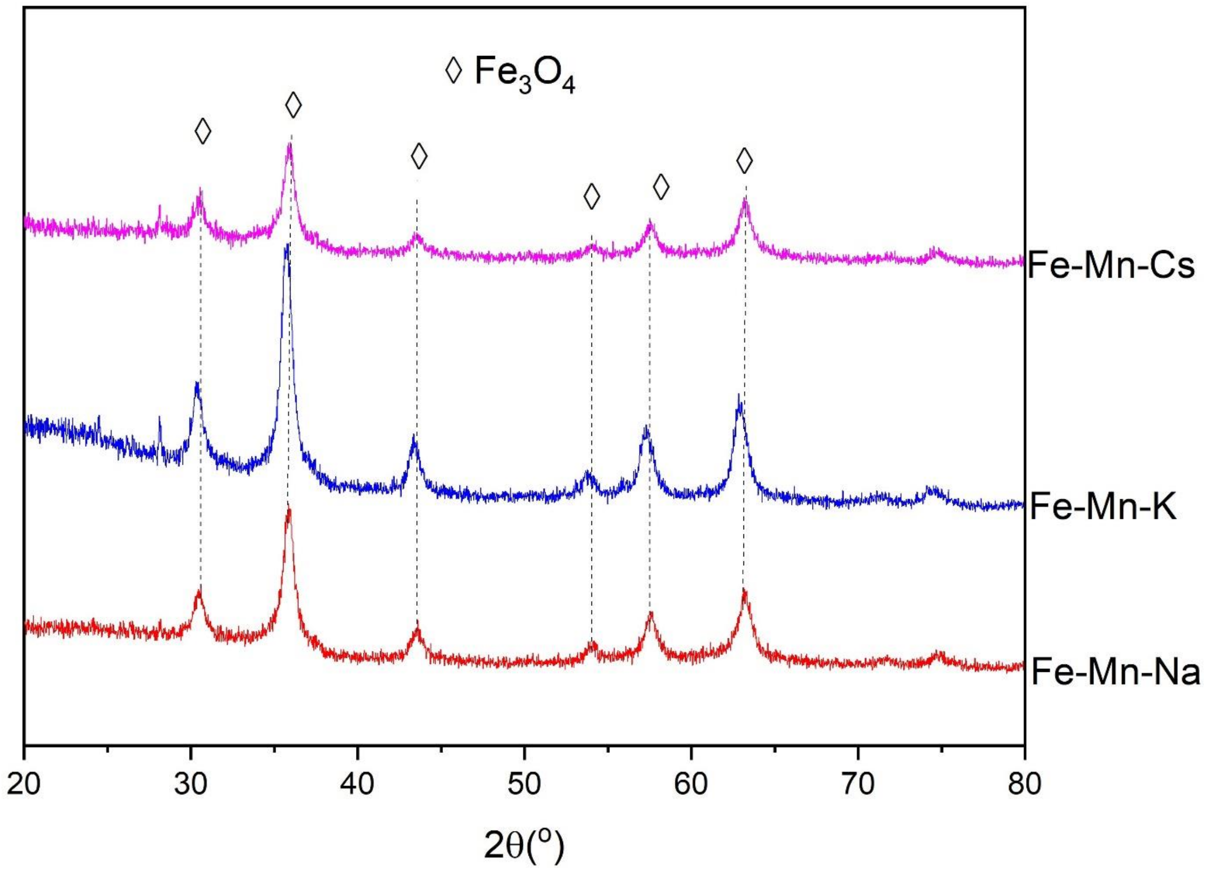

Supplementary Figure 3 XRD spectrum of the catalysts (different base metal) prepared with citric acid method

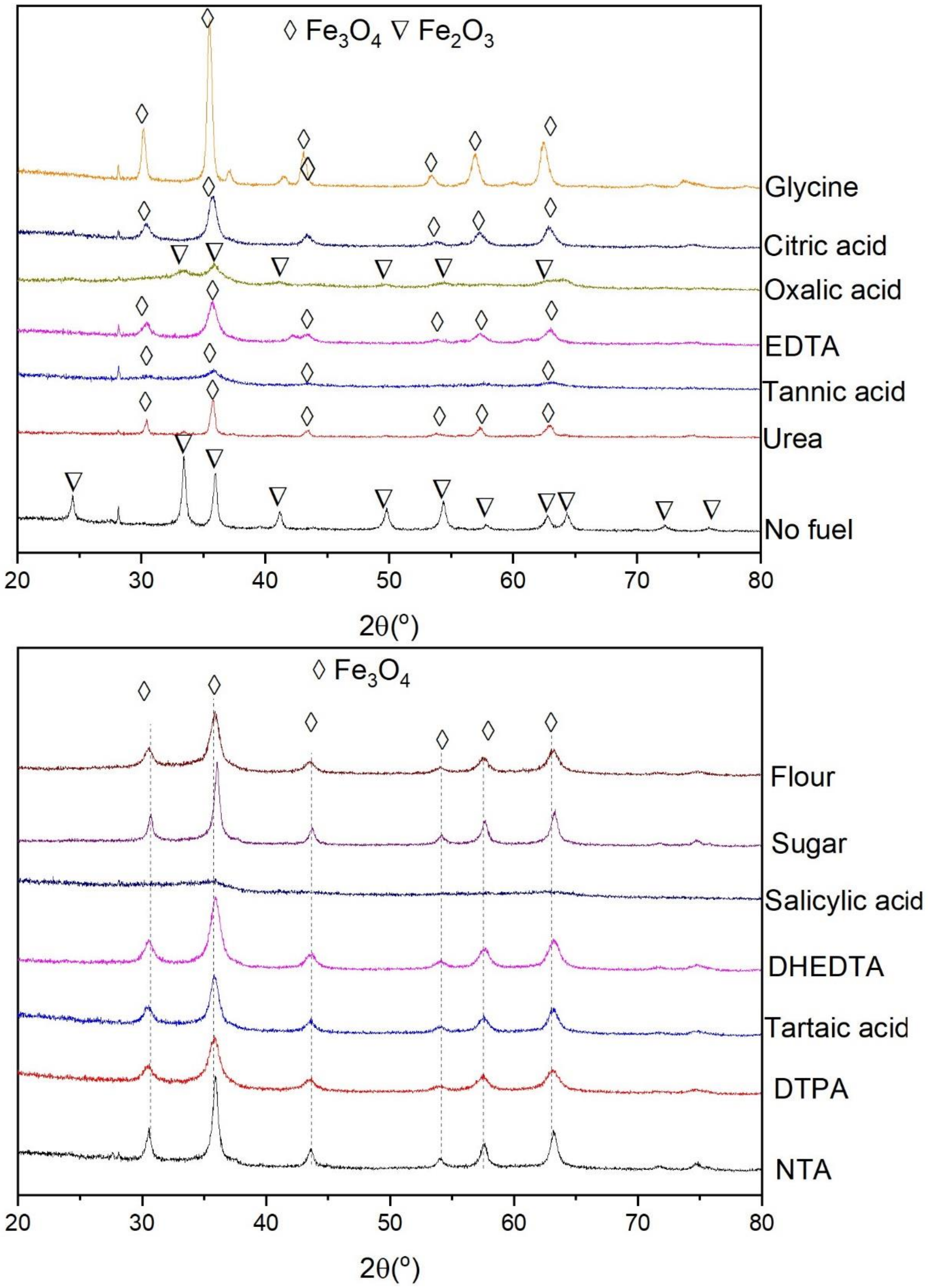

Supplementary Figure 4 XRD spectrum of the catalysts prepared with different organic compounds

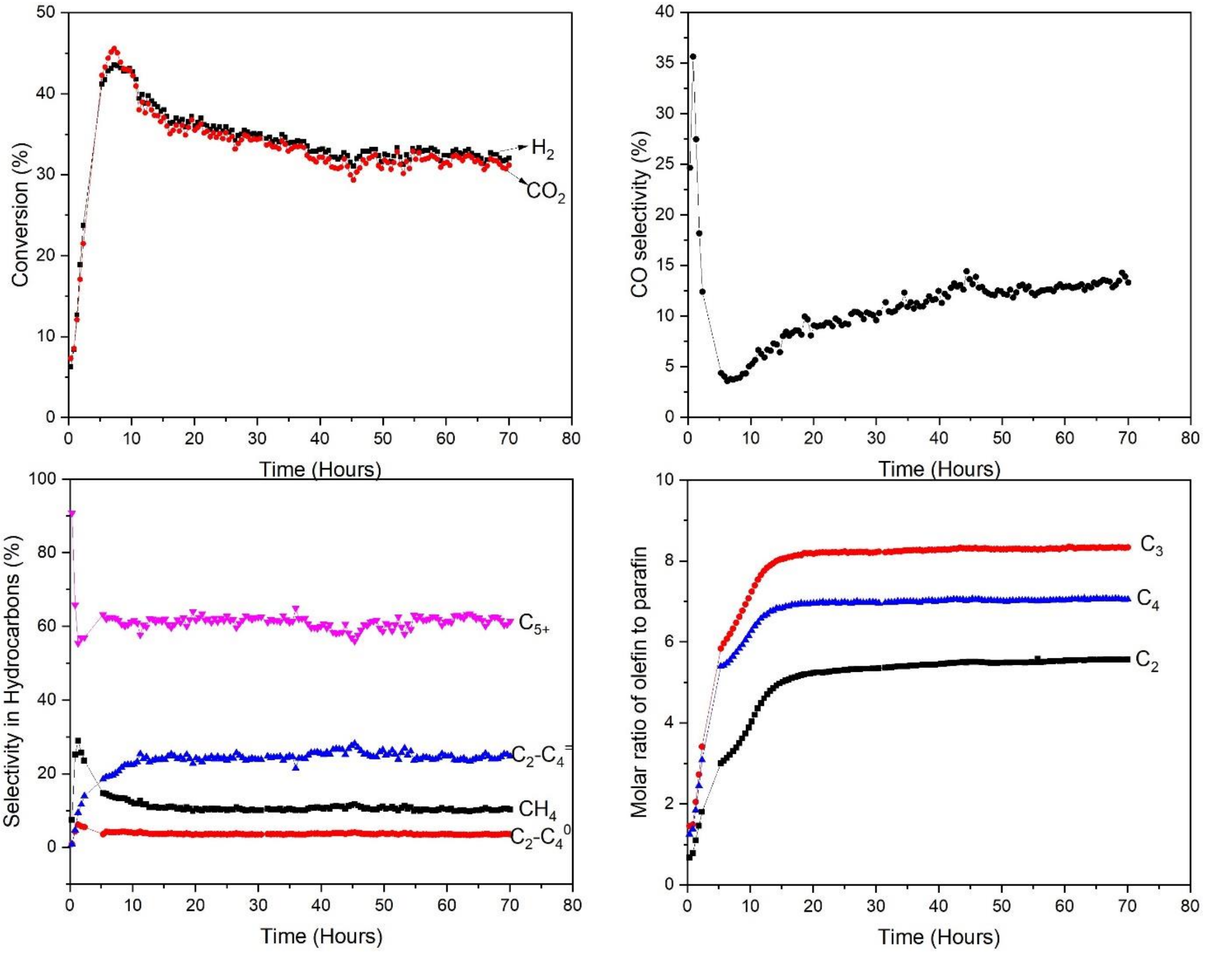

Supplementary Figure 5 Catalytic performance of CO_2 hydrogenation on catalyst Fe-Zn- K

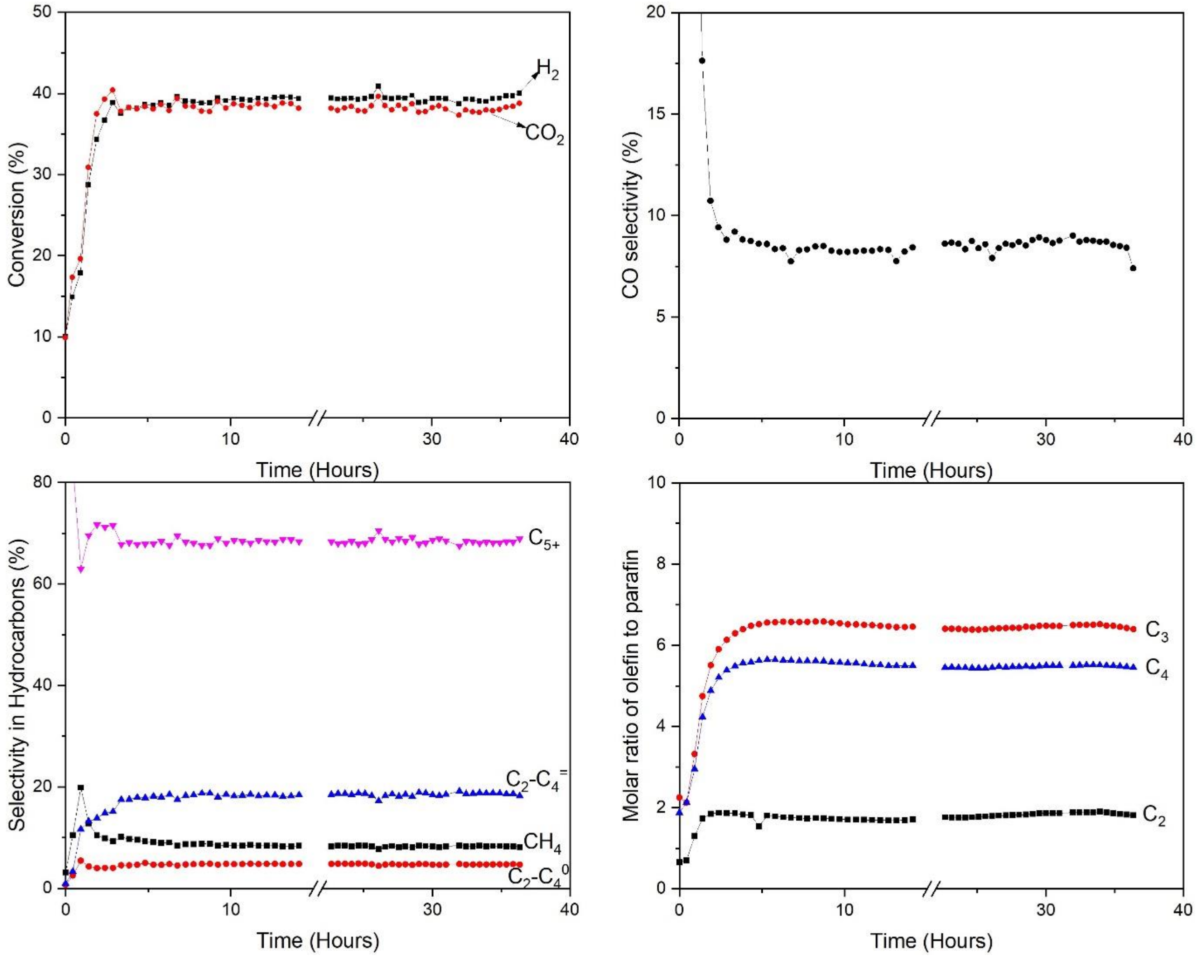

Supplementary Figure 6 Catalytic performance of CO_2 hydrogenation on catalyst Fe-Cu- K

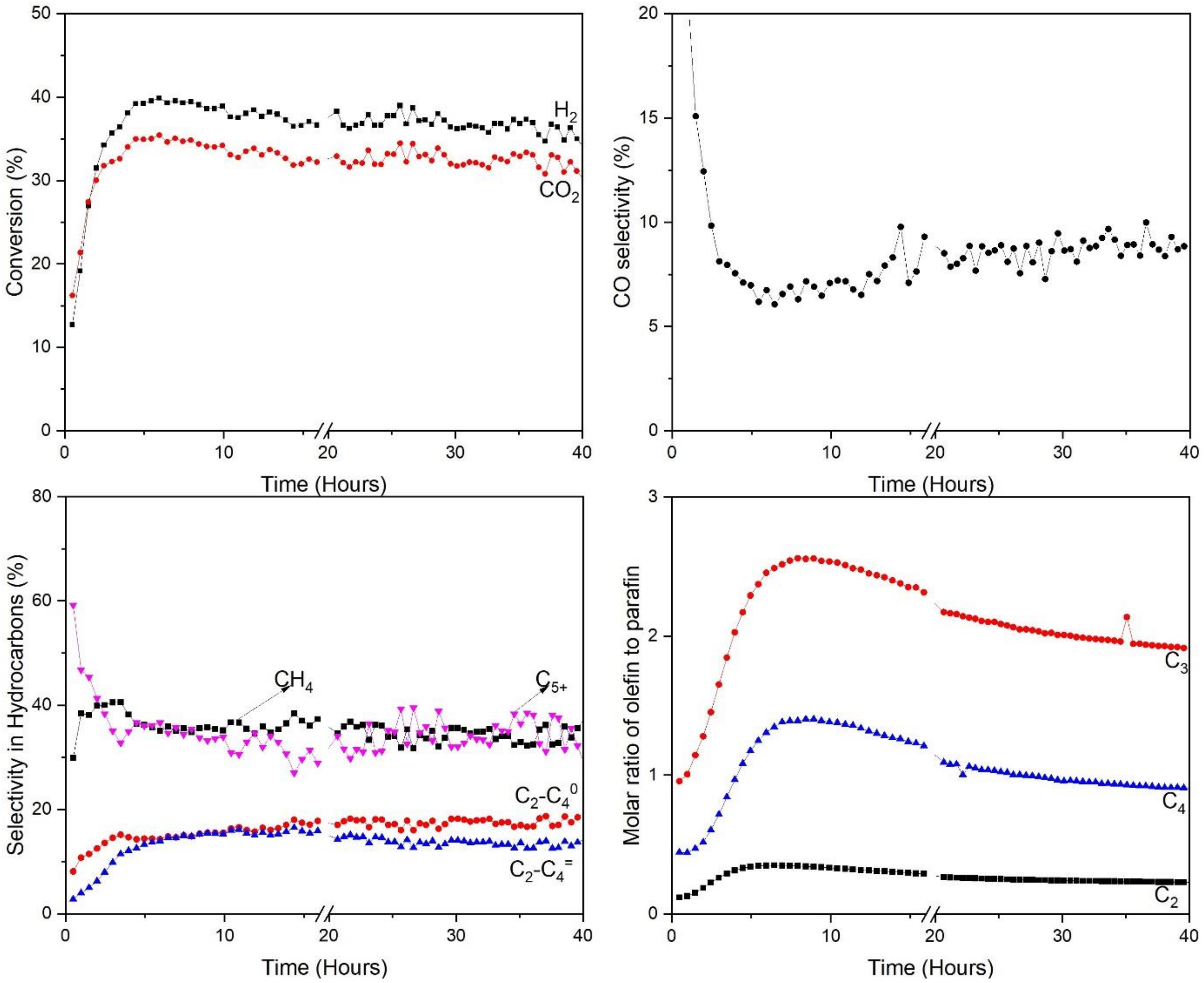

Supplementary Figure 7 Catalytic performance of CO_2 hydrogenation on catalyst Fe-Mn-Li

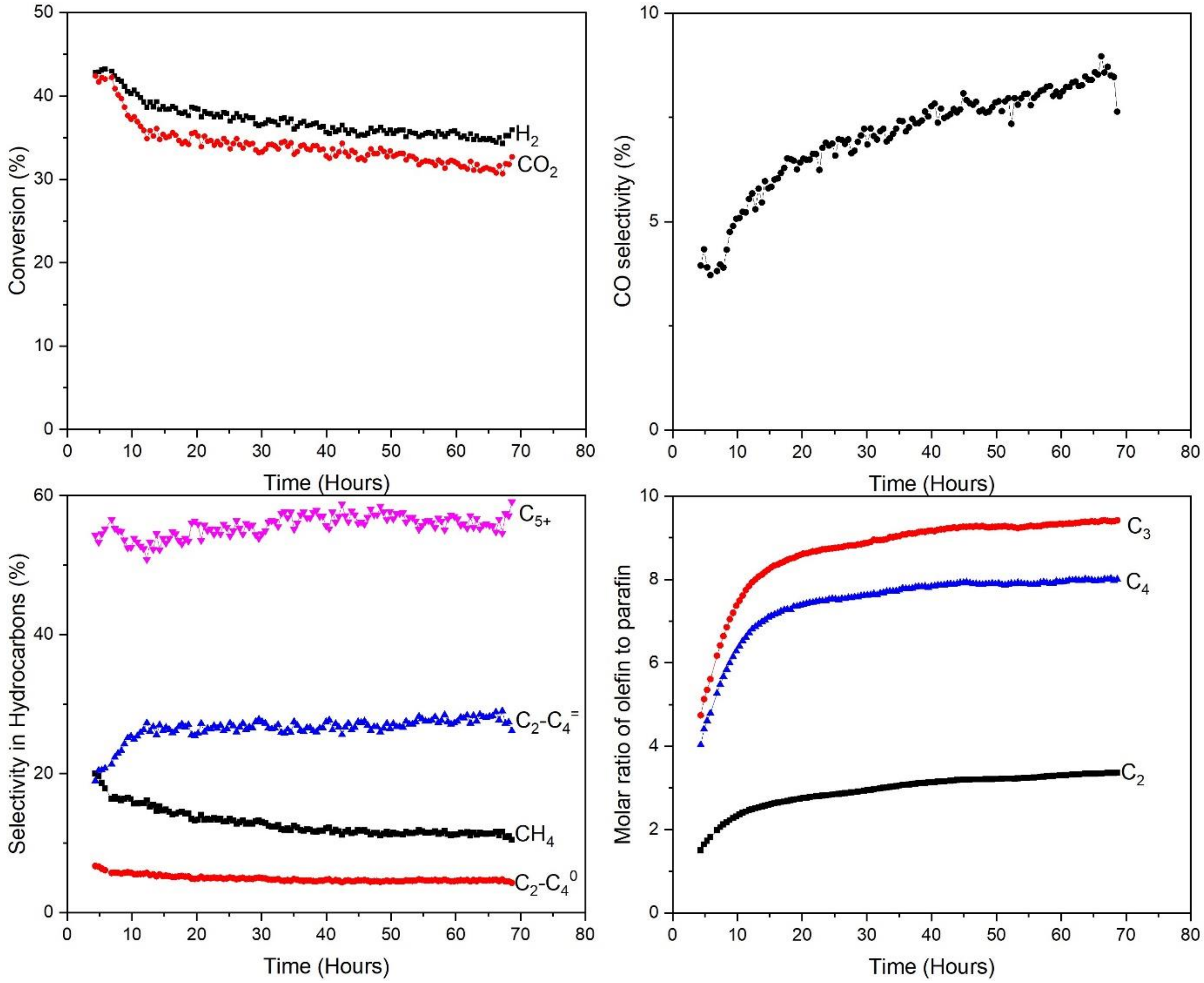

Supplementary Figure 8 Catalytic performance of CO_2 hydrogenation on catalyst Fe-Mn-Na

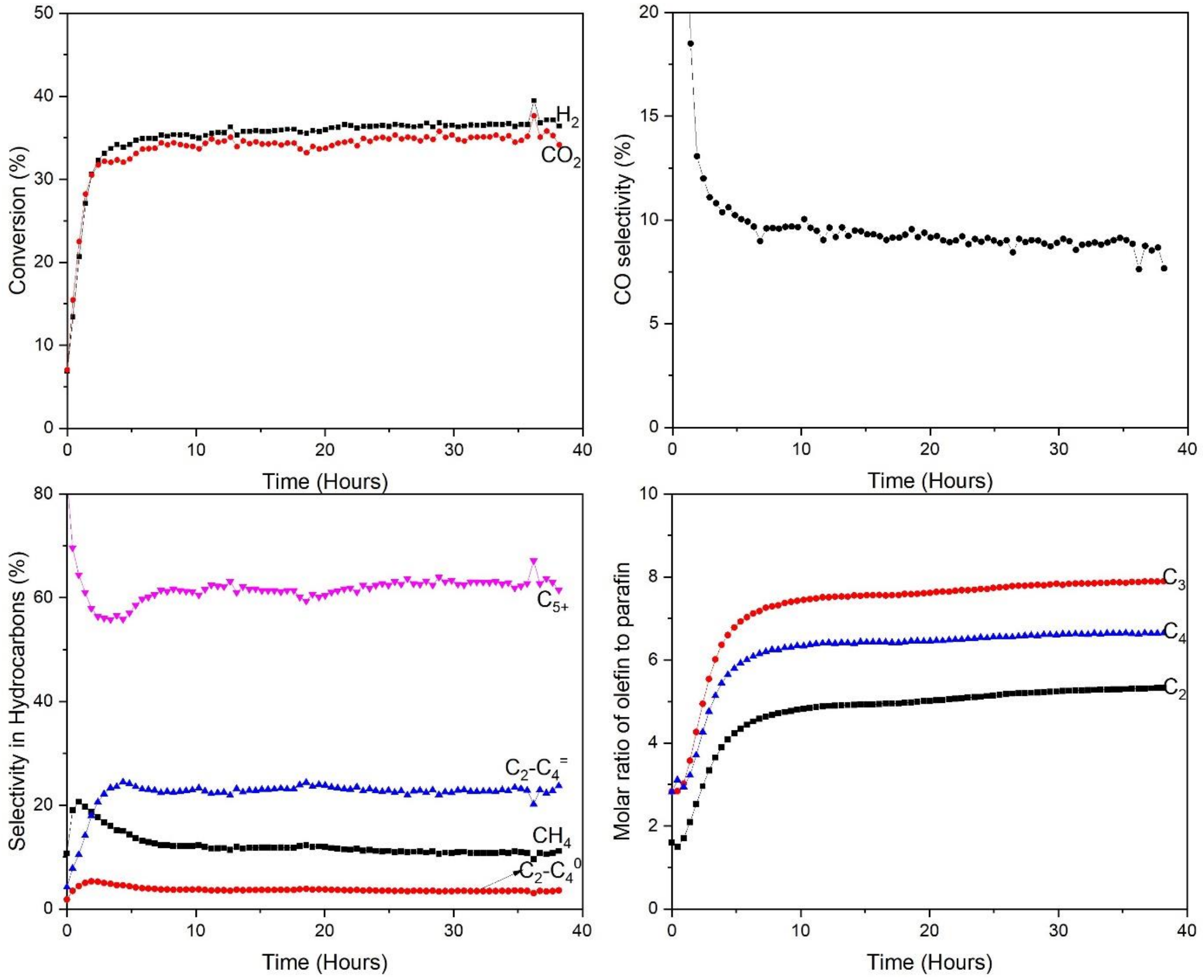

Supplementary Figure 9 Catalytic performance of CO_2 hydrogenation on catalyst Fe-Mn-Cs

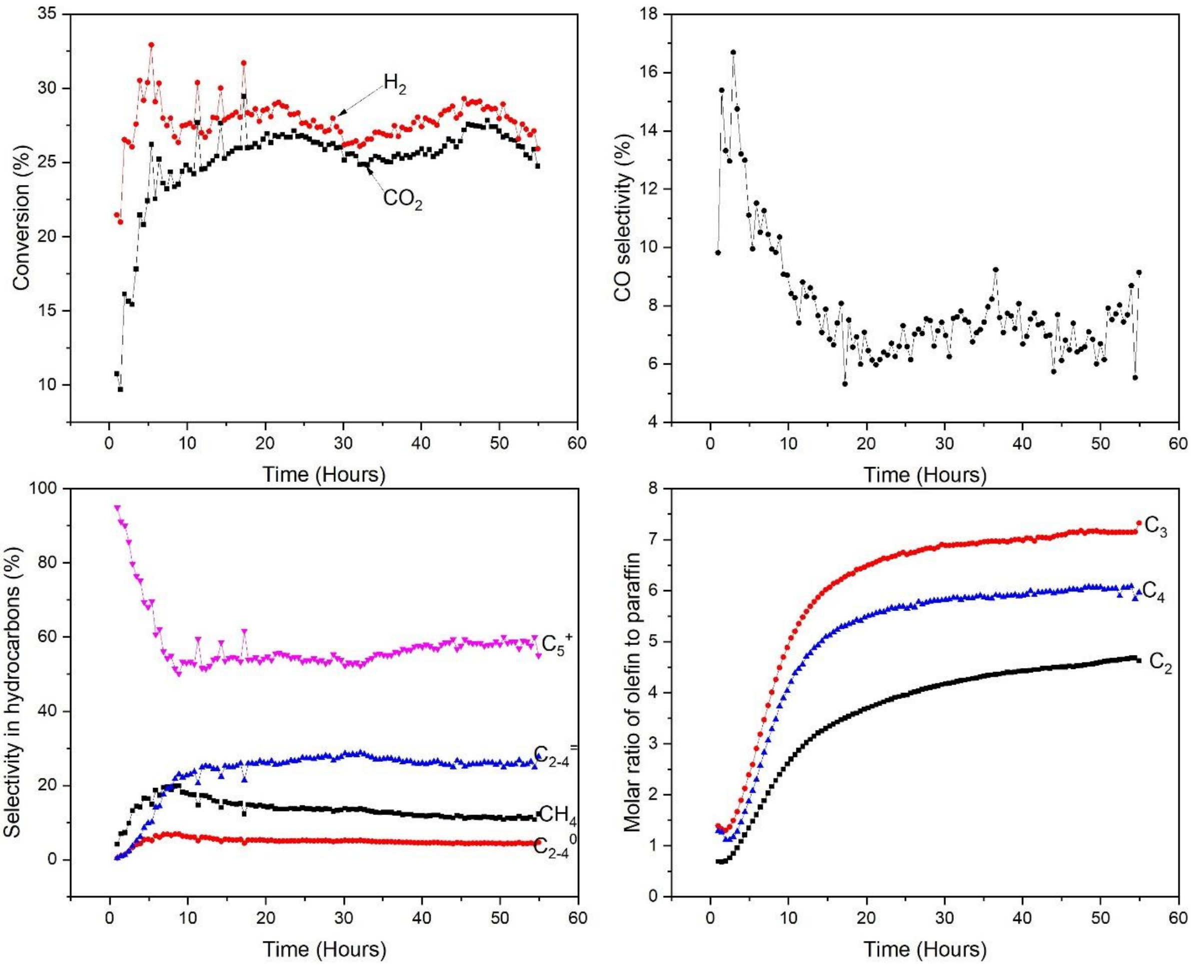

Supplementary Figure 10 Catalytic performance of CO_2 hydrogenation on catalyst Fe-Mn- K without fuel method

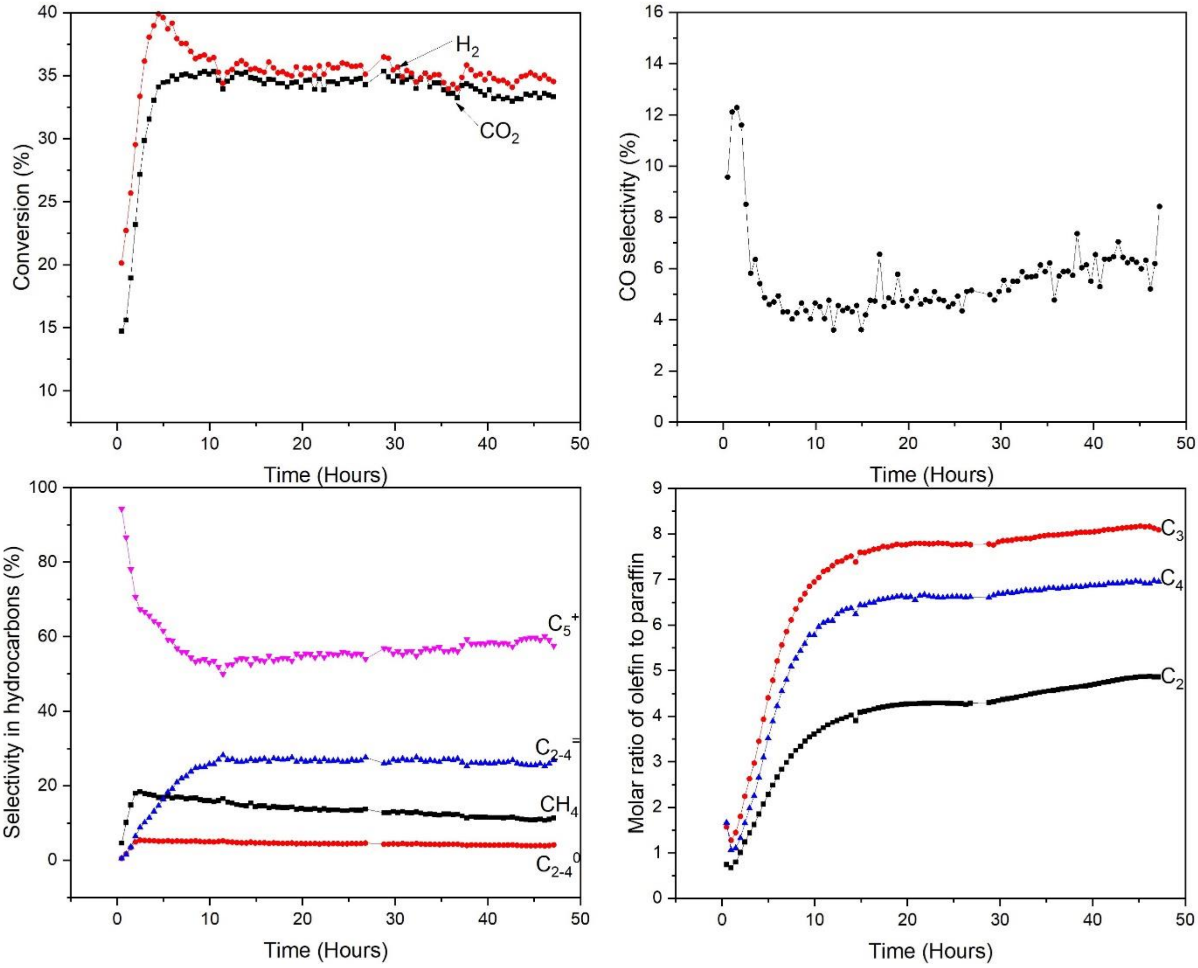

Supplementary Figure 11 Catalytic performance of CO_2 hydrogenation on catalyst Fe-Mn- K with urea method

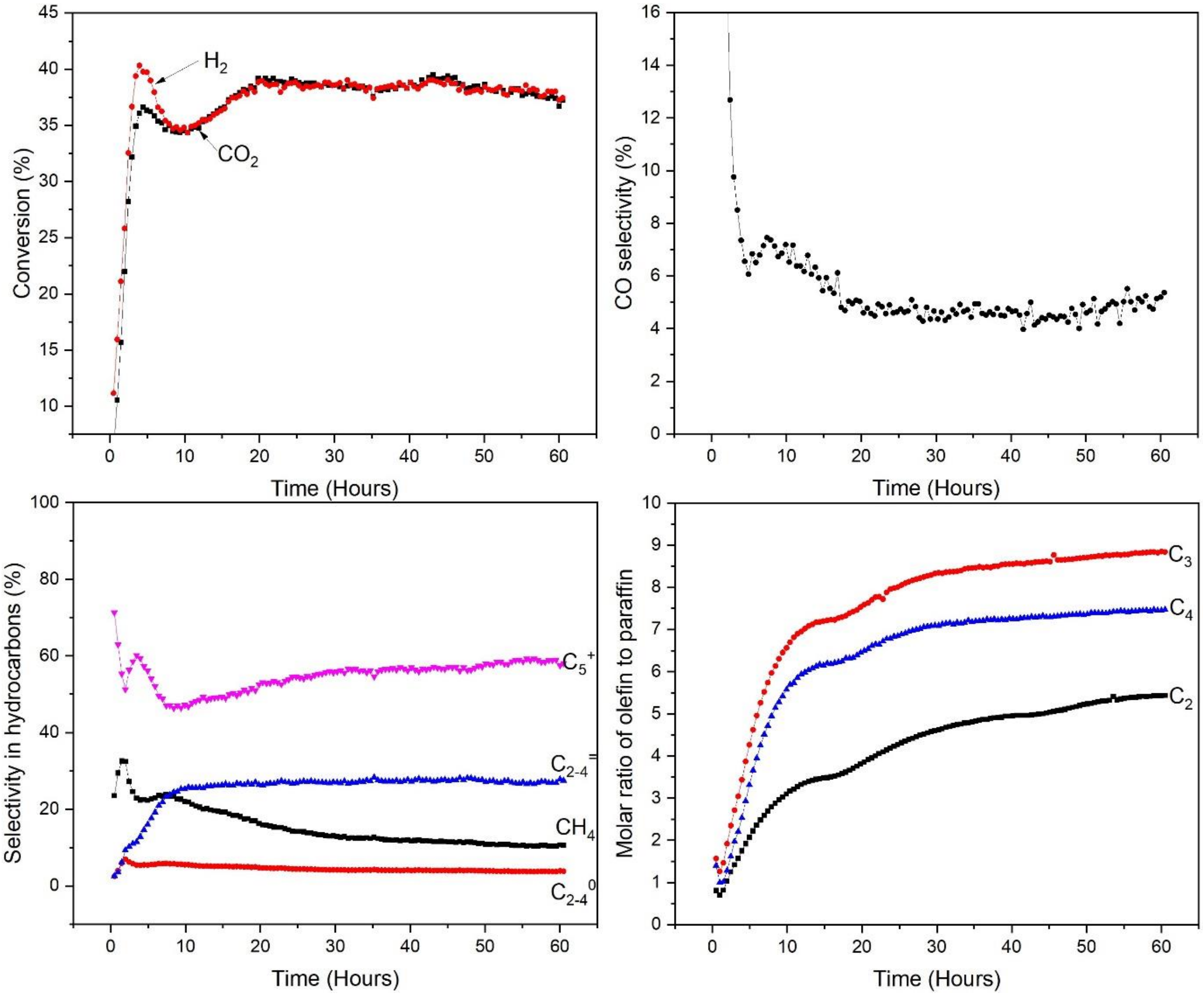

Supplementary Figure 12 Catalytic performance of CO_2 hydrogenation on catalyst Fe-Mn- K with tannic acid method

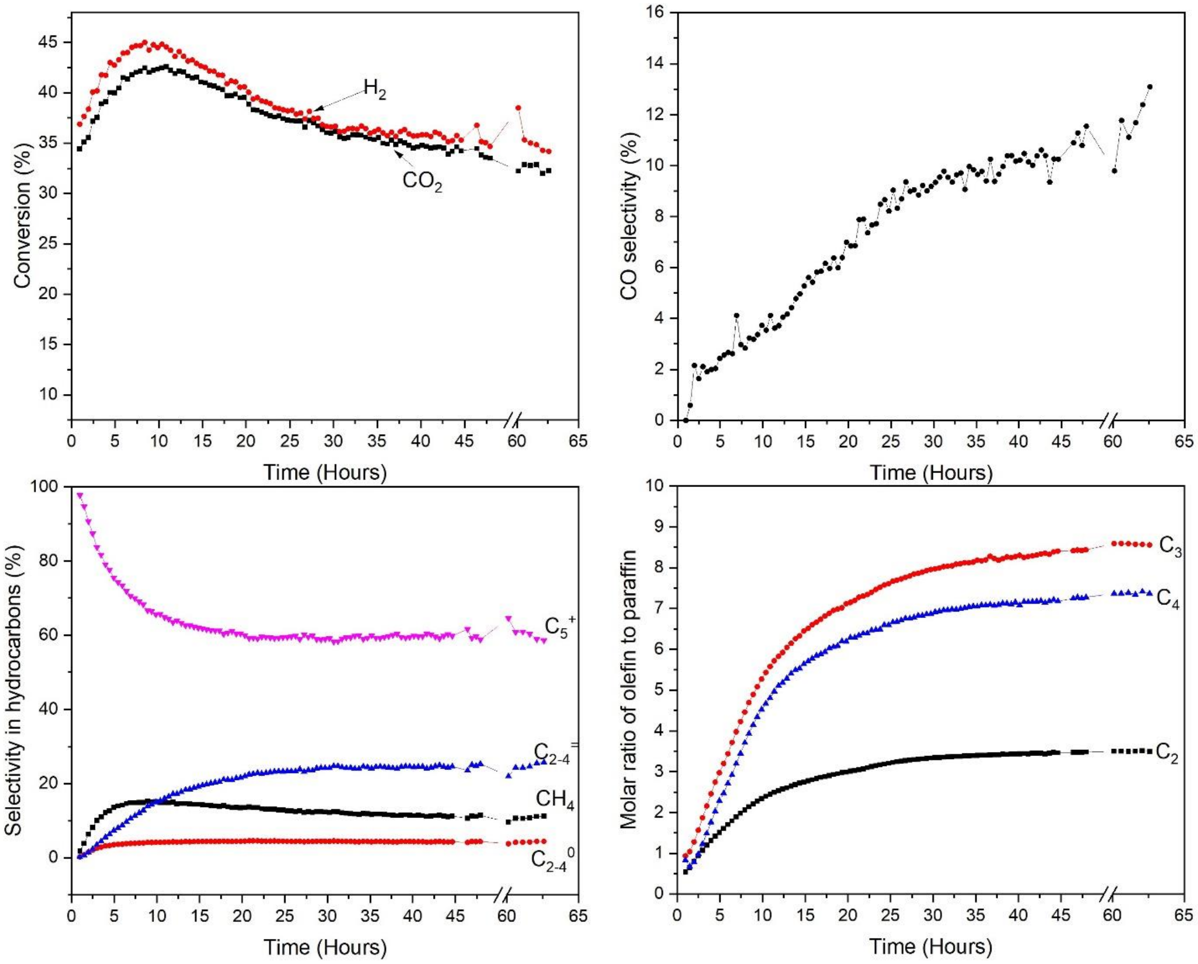

Supplementary Figure 13 Catalytic performance of CO_2 hydrogenation on catalyst Fe-Mn- K with EDTA method

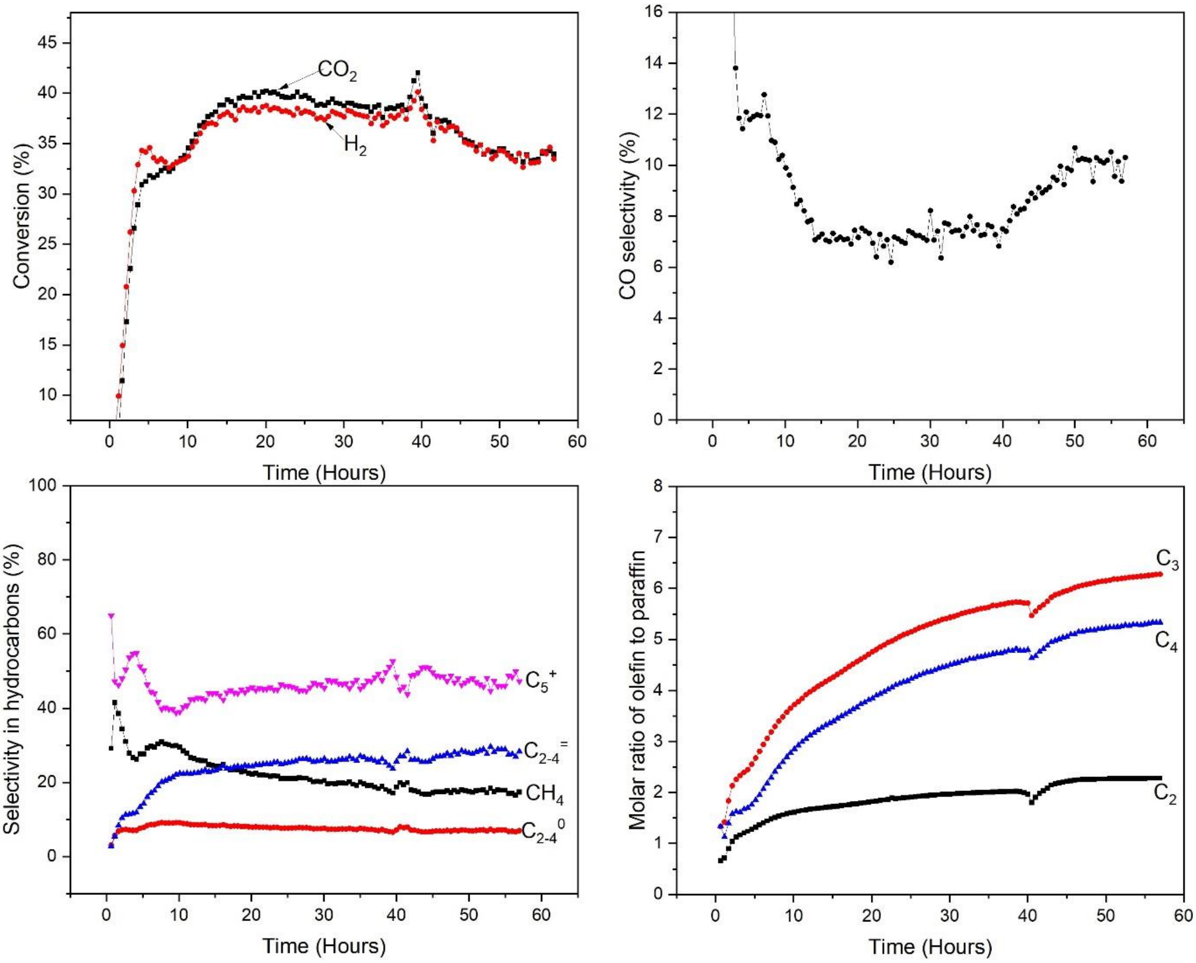

Supplementary Figure 14 Catalytic performance of CO_2 hydrogenation on catalyst Fe-Mn- K with glycine method

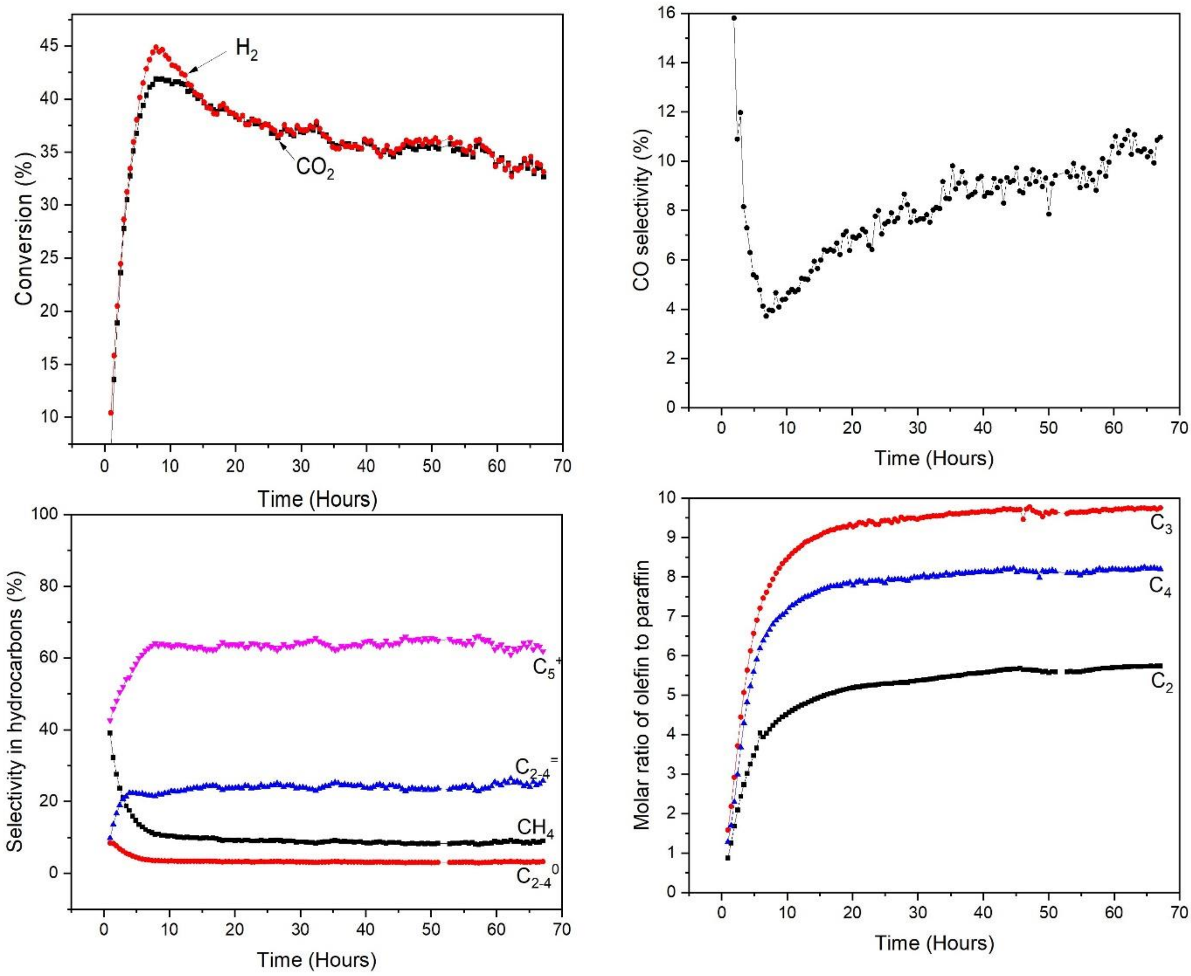

Supplementary Figure 15 Catalytic performance of CO_2 hydrogenation on catalyst Fe-Mn- K with oxalic acid method

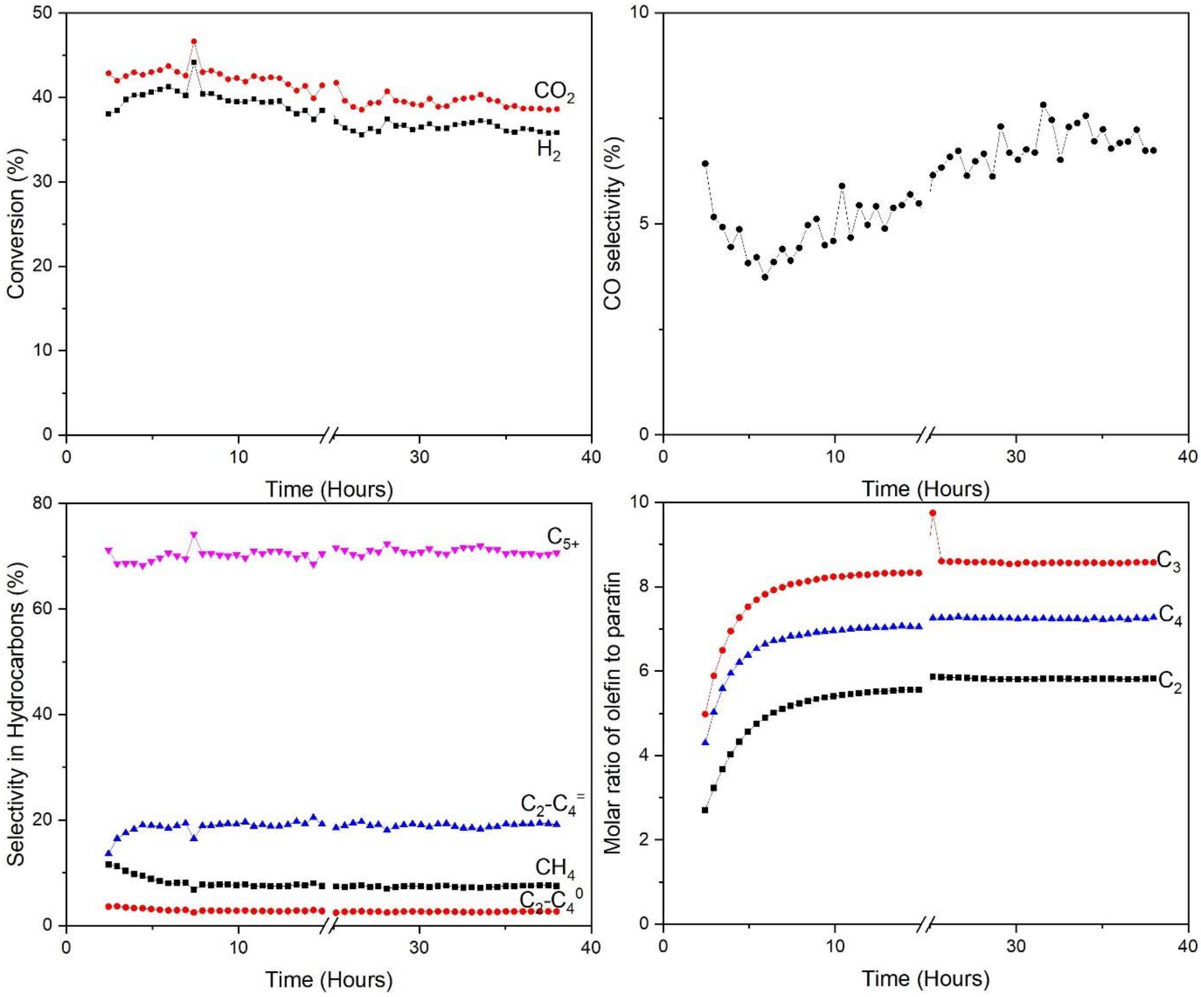

Supplementary Figure 16 Catalytic performance of CO_2 hydrogenation on catalyst Fe-Mn-K with NTA method

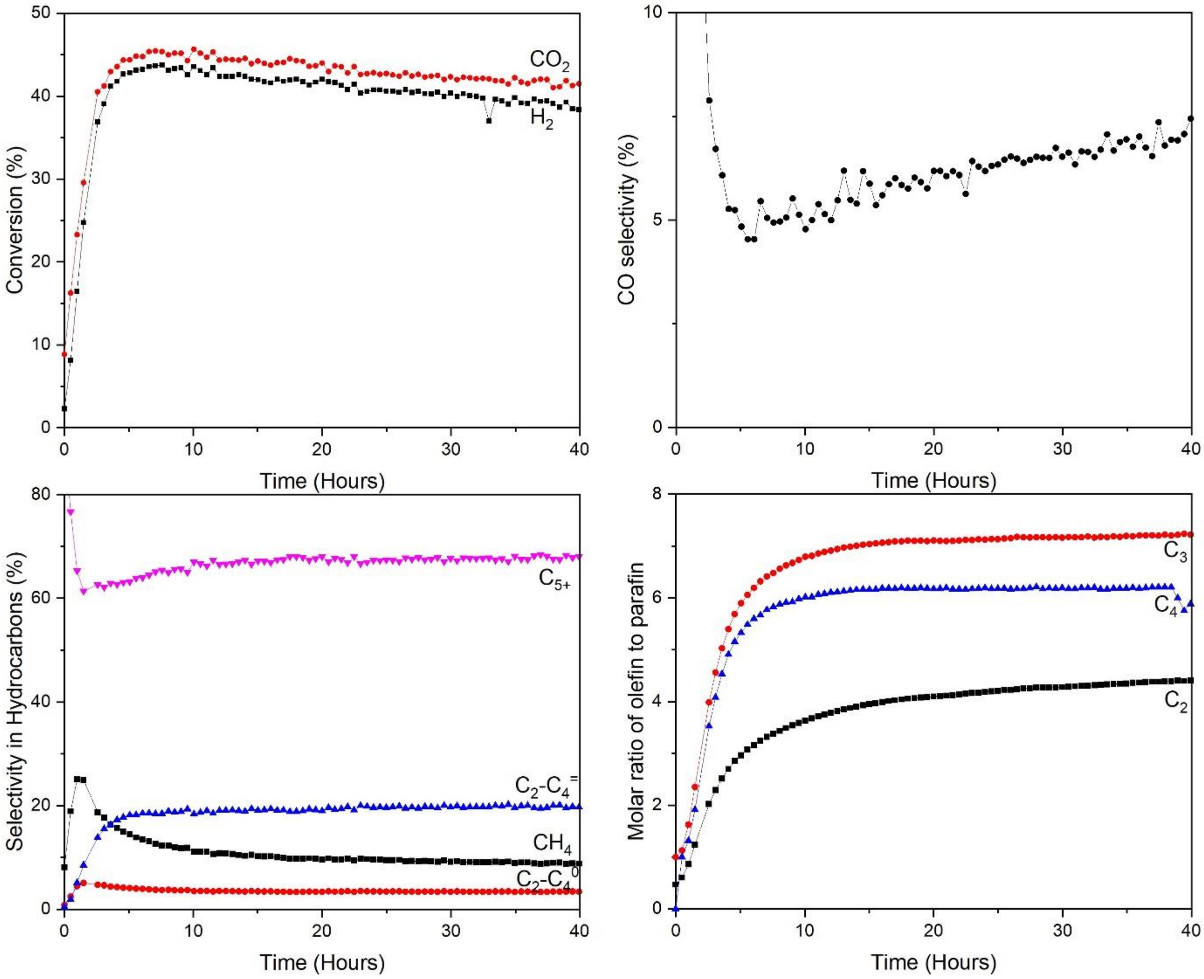

Supplementary Figure 17 Catalytic performance of CO_2 hydrogenation on catalyst Fe-Mn-K with DTPA method

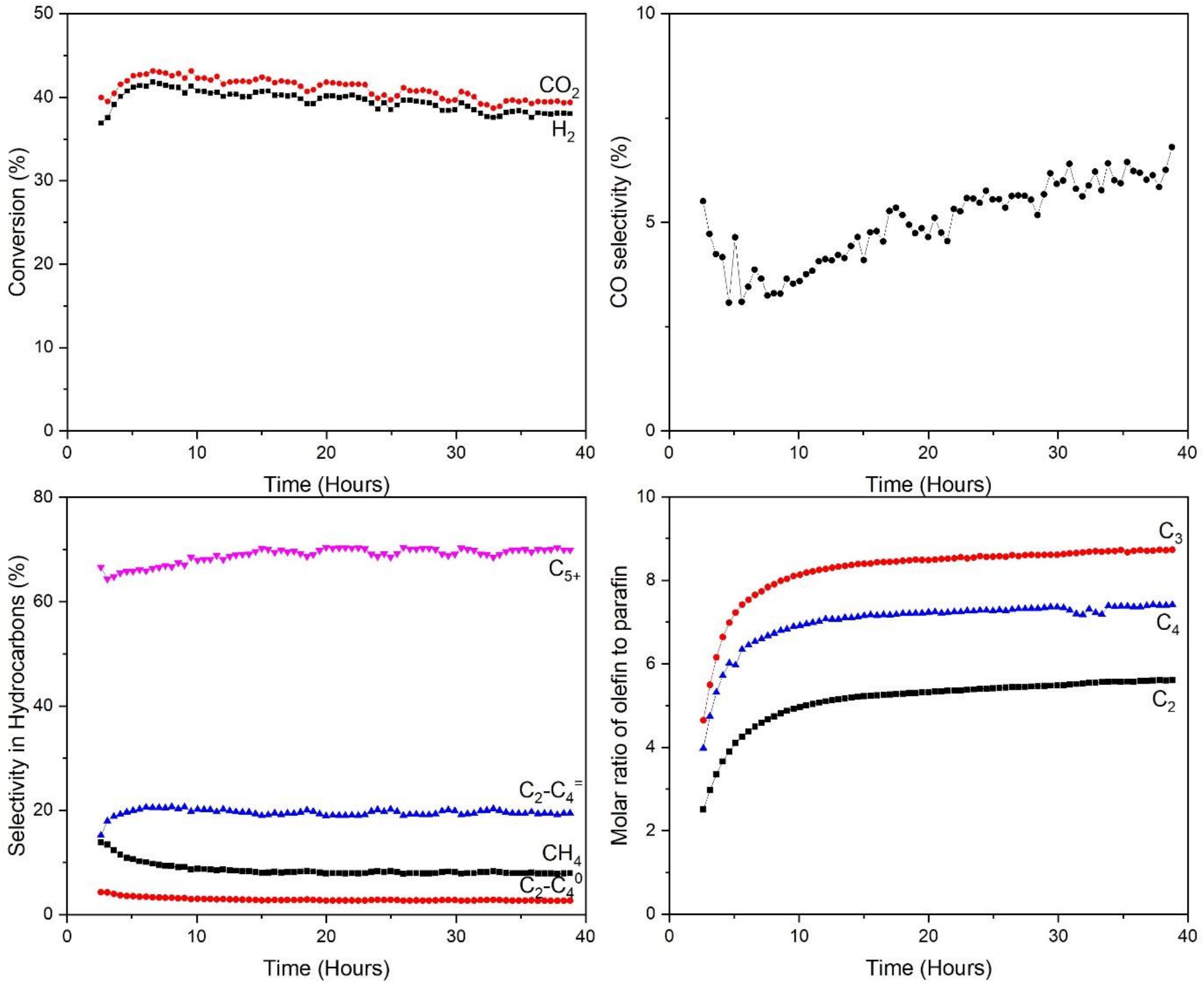

Supplementary Figure 18 Catalytic performance of CO_2 hydrogenation on catalyst Fe-Mn-K with tartaric acid method

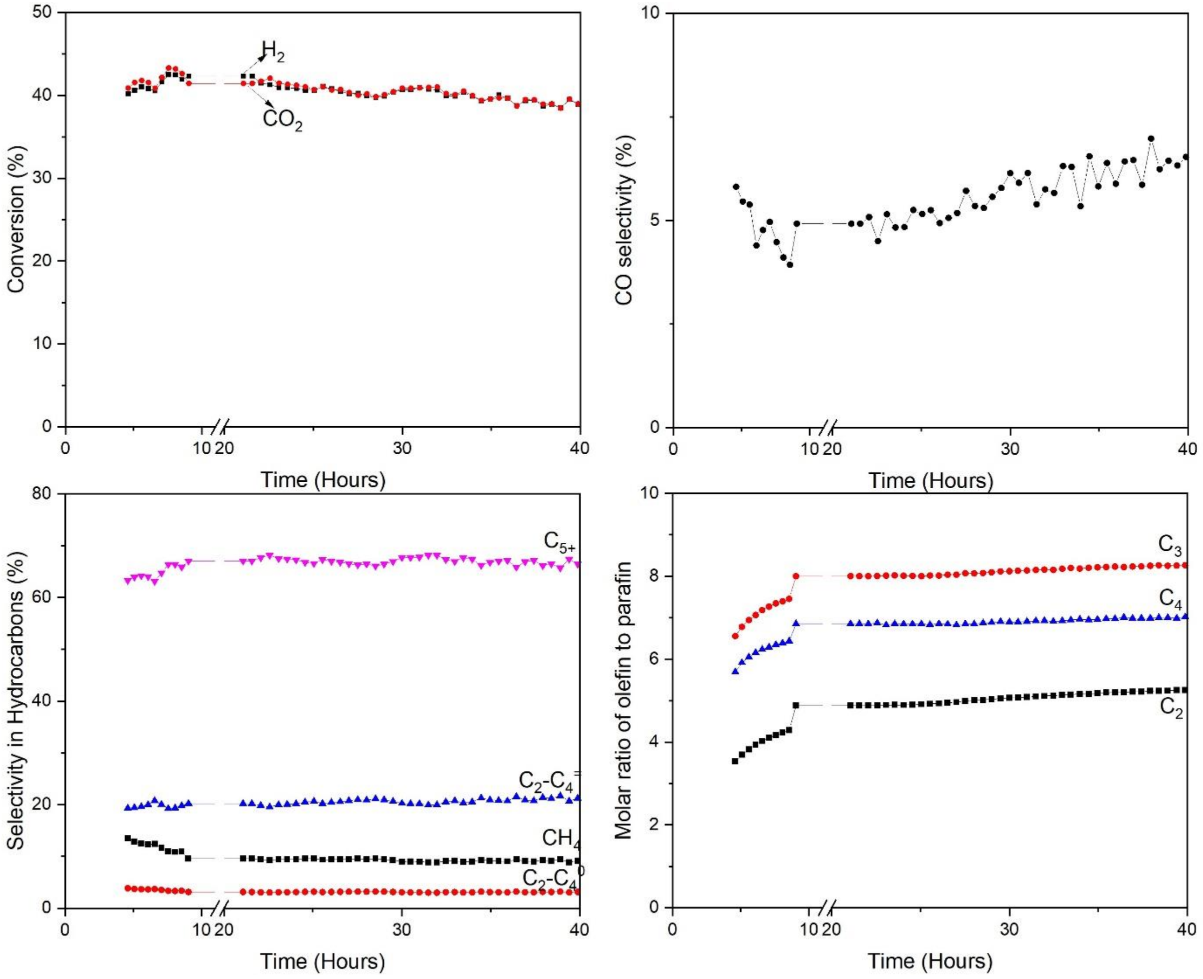

Supplementary Figure 19 Catalytic performance of CO_2 hydrogenation on catalyst Fe-Mn-K with HEDTA method

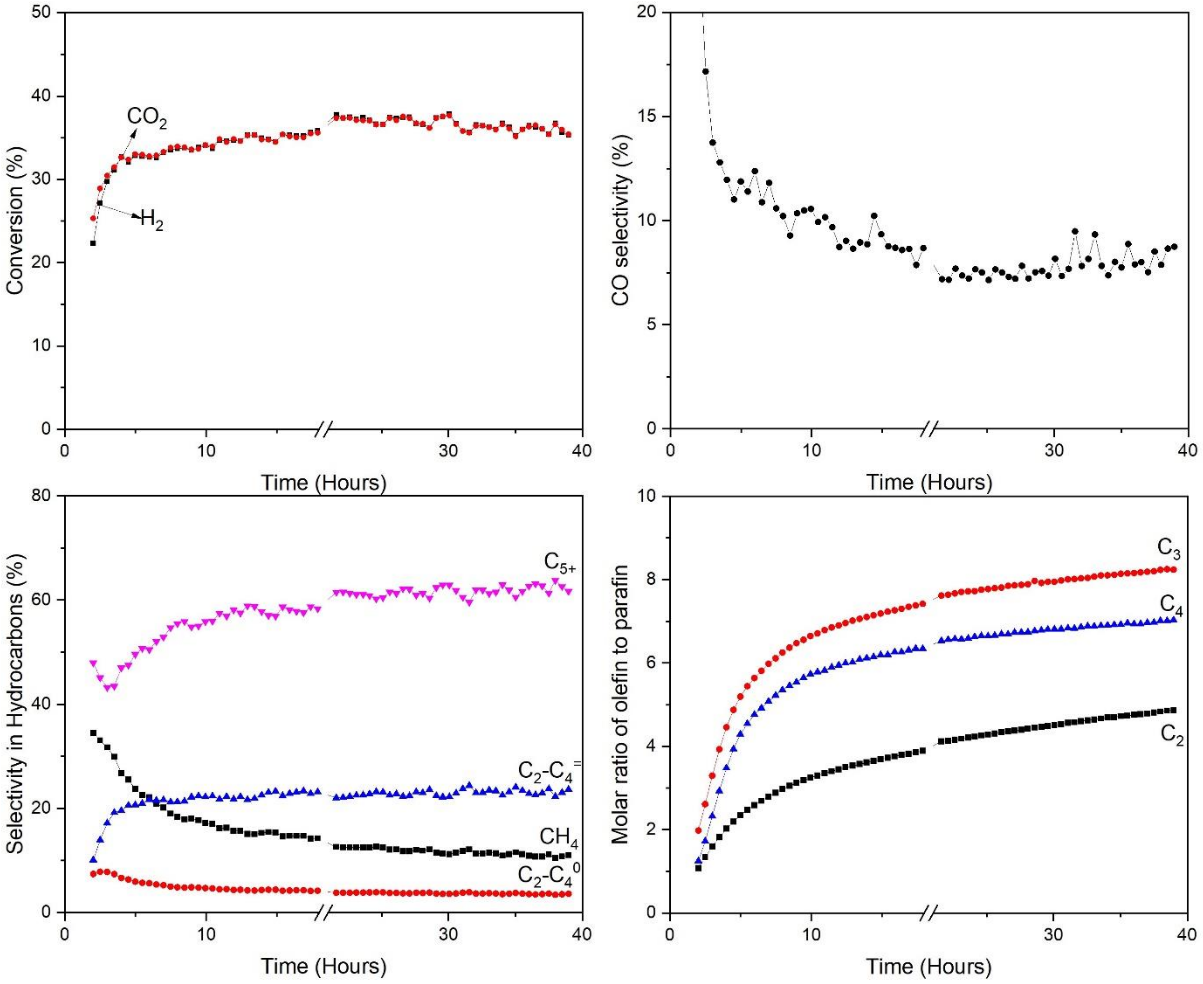

Supplementary Figure 20 Catalytic performance of CO_2 hydrogenation on catalyst Fe-Mn-K with Salicylic acid method

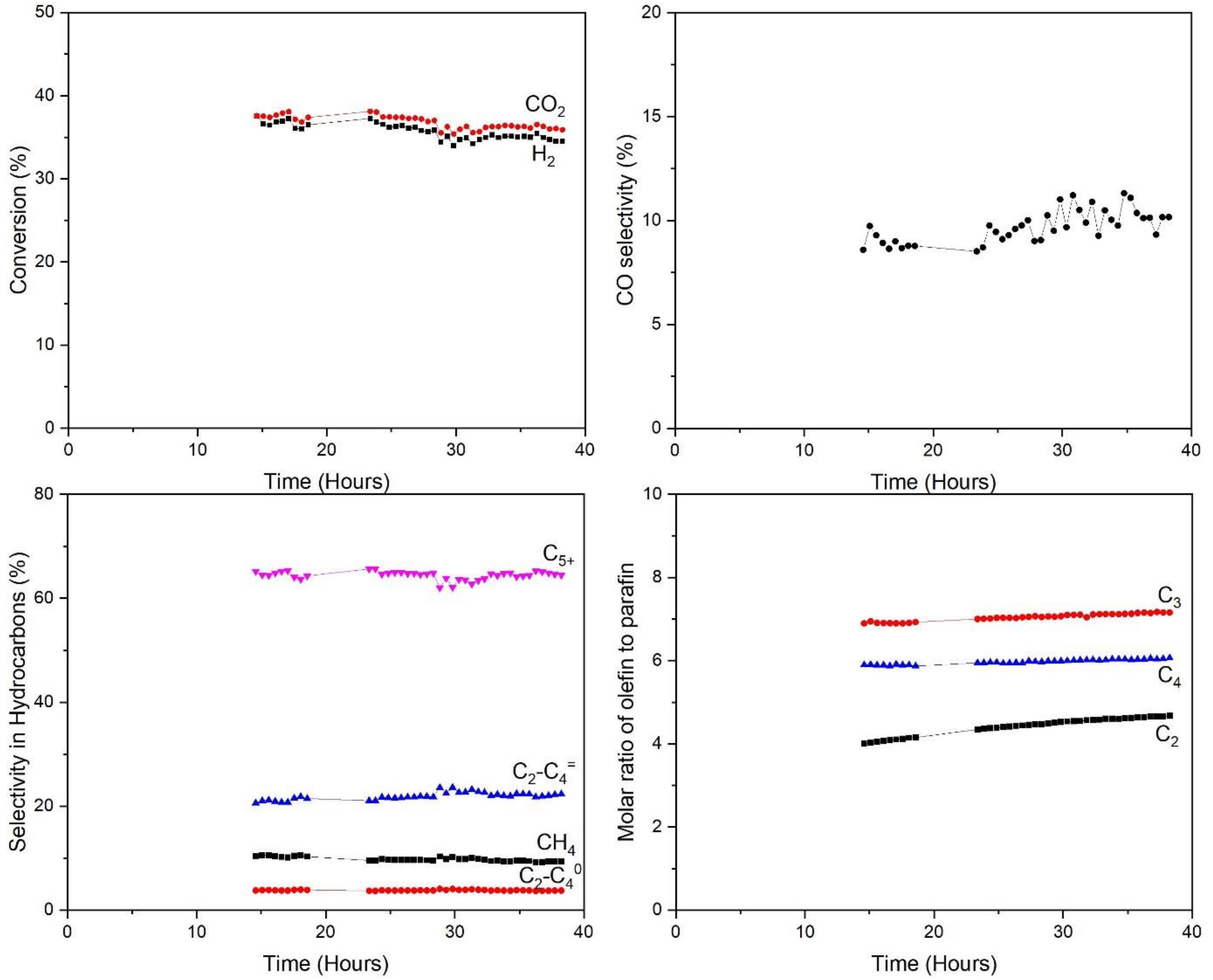

Supplementary Figure 21 Catalytic performance of CO_2 hydrogenation on catalyst Fe-Mn-K with sugar method

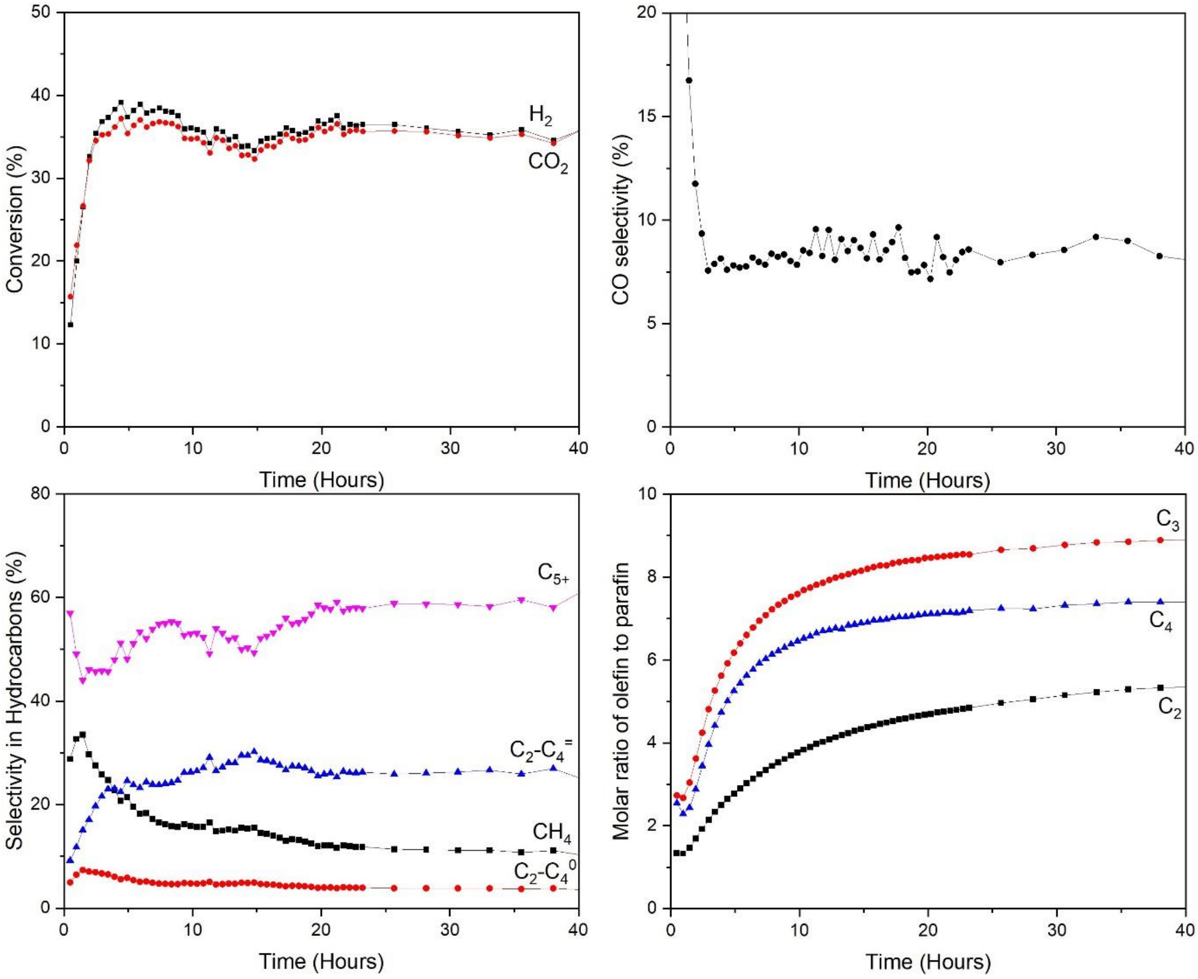

Supplementary Figure 22 Catalytic performance of CO_2 hydrogenation on catalyst Fe-Mn-K with flour powder method

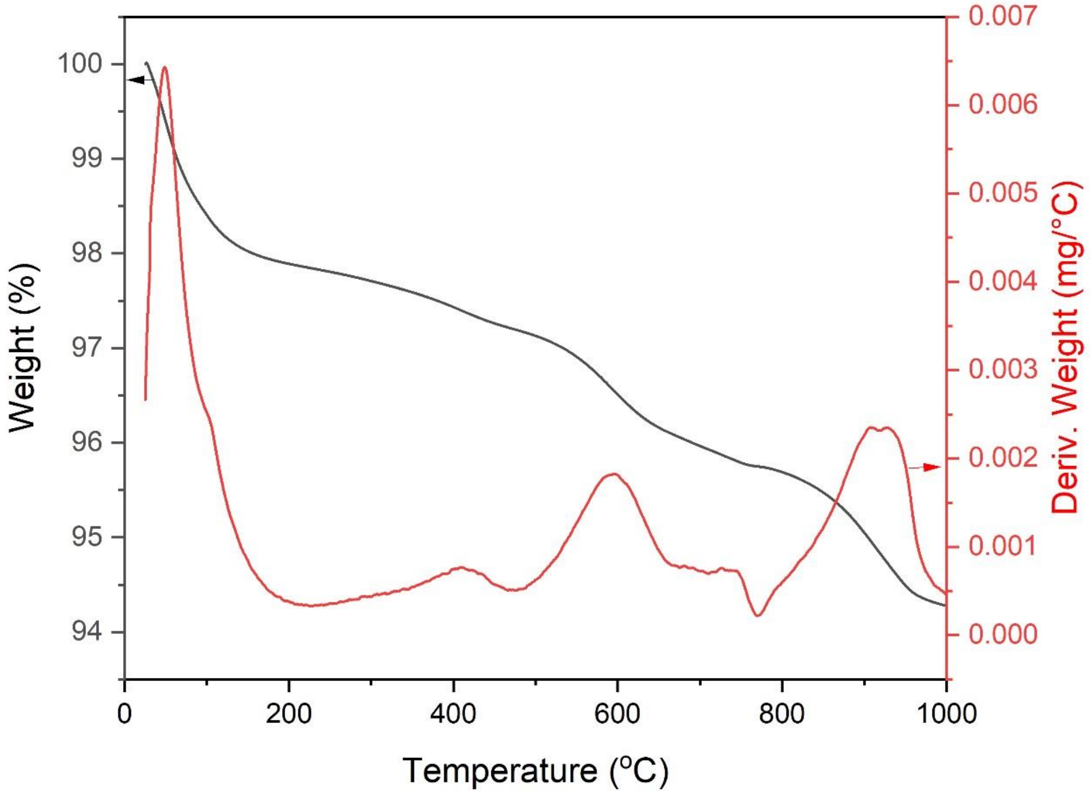

Supplementary Figure 23 TGA results of catalyst precursor of Fe-Mn-K (citric acid method)

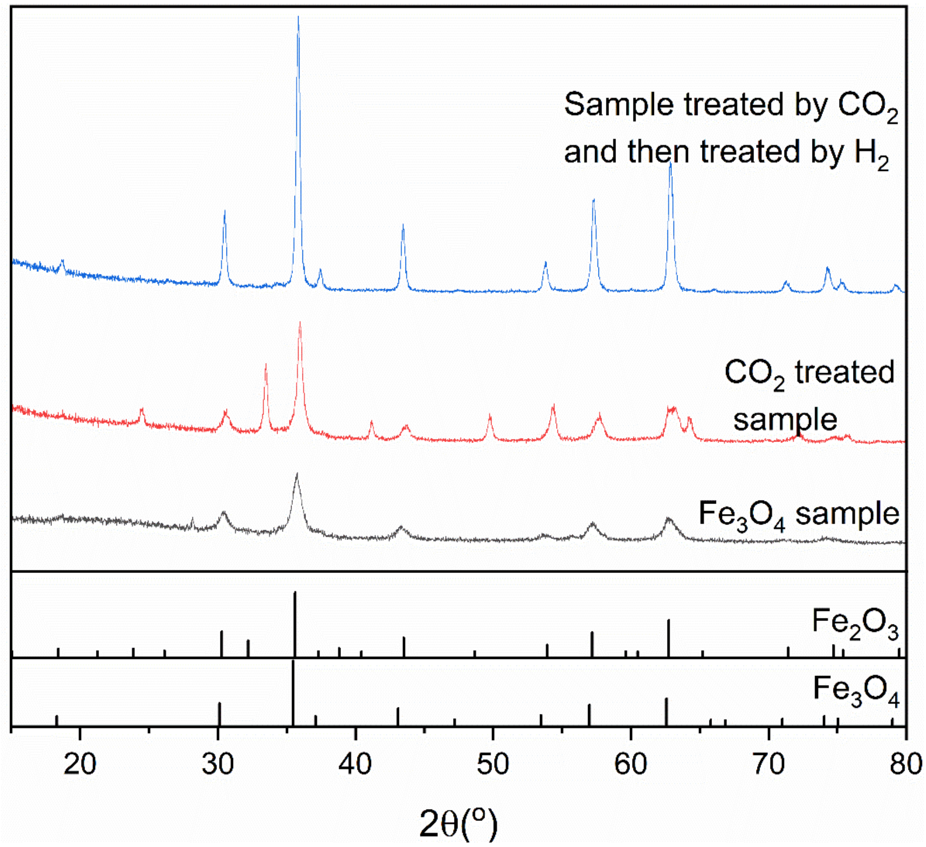

Supplementary Figure 24 XRD spectrum of Fe_3O_4 sample (black), sample treated at CO_2 atmosphere 350 °C for 16 hours(red) and sample treated at CO_2 atmosphere and then treated at 5%H_2/N_2 atmosphere at 350 °C for 16 hours respectively (blue)
