# Supplementary material for: Transforming carbon dioxide into jet fuel using an organic combustion-synthesized Fe-Mn-K catalyst
Source: Nat Commun. 2020 Dec 22;11:6395. doi: 10.1038/s41467-020-20214-z (PMC7755904; doi:10.1038/s41467-020-20214-z)
Supplement: Supplementary file 2 — Peer Review File [file 41467_2020_20214_MOESM2_ESM.pdf]

Reviewer #1 (Remarks to the Author):

The manuscript deals with the application of the method of organic combustion for the synthesis of an efficient catalysts for CO2 hydrogenation directly to Jet fuel. The catalysts prepared by this way demonstrate high activity at high selectivity to C5+ hydrocarbons and low selectivity to methane and CO which is usually for these types of materials. This is an interesting study and I would recommend this manuscript for publication. However, there are several important points which have to be clarified:

1. It would be important to provide ASF distribution of the formed products to compare with existing catalysts. It seems that chain growth probability is relatively high which makes it similar to low temperature FT synthesis but with a high contribution of olefins.
2. The catalyst Fe-Mn-K has been prepared by calcination at 350C. Probably there is still a small amount of carbon from the organic compounds which would explain higher selectivity to C5+ products. I would suggest perform TG analysis to check if the catalyst is carbon-free after this treatment.
3. It is not clear what is the role of access to O2 during catalyst preparation and how authors controlled the temperature. There is no O2 in the proposed equation. In other case oxidation of Fe salt should proceed till Fe2O3. It would be important to give more details and comments on on this procedure.
4. There is no clear correlation between the type of fuel, crystalline sizes and catalytic performance, however, small polyacids (tartaric acid, DTPA…) seem to have better activity and selectivity. It would be important to provide some vision about the efficiency and the role of fuels in this procedure. The possible explanation could be in higher intimacy between Fe, Mn and K in the catalysts prepared by OCM. Additional characterization to clarify the interaction between components would be highly desirable here.
5. The authors provide the mechanism of transformation Fe3O4, carbide and Fe2O3 to each other, however, there is no clear evidence in manuscript about it. I would recommend to perform model reactions to confirm this mechanism. For example, by treatment of the catalyst by CO2 demonstrate formation of Fe2O3 from Fe3O4 and reduction of Fe2O3 by H2 to show formation of Fe3O4.

Reviewer #2 (Remarks to the Author):

The authors present a preparation procedure for catalysts with relatively high yield to higher hydrocarbons (jet fuel) from CO2. In my opinion this is solid and interesting work. However, I am not sure that this work provides truly novel insights, especially as the characterization/explanation for the observed trends is meager.

Furthermore, I miss a rationale in the flow of the story for why this catalyst would work after the introduction (which is an excellent introduction) that most catalysts don't work to this extent to convert CO2 to jet fuels, before the current results. Even though the catalyst preparation procedure is explained in the methods an materials, a quick introduction and rationale should be included before the results are delved into.

I am also wondering about the carbide phase formed and its contribution to the products. I would like to see isotopic labelling and an understanding of the contribution of Mars van Krevelen type mechanisms.

XRD - Claim that Mn and K are not observed and are thus highly disperse simply does not hold true. There can be clusters of up to 2 nm of pure material and still be no reflections due to the periodicity needed in XRD. It is best to verify such claims with methods that are more sensitive, for example simple XAS measurements which might be relatively easy to obtain given the affiliations.

Generally speaking, some attention should be given to the figures which could be more attractive/self explanatory (not a scientific comment per se but it may help the authors to better structure the story as well).

Blank experiments with a typical catalyst preparation procedure should also be included to compare the used setup/materials to the available literature.

The proposed reaction scheme is not based on much, something like in-situ spectroscopy is missing, or at the very least the proposed isotopic labelling experiments.

I realize the experiments I suggest may require some significant effort, and also realize that the work can be seen as currently complete. Thus it is, in my opinion, up to the authors to decide to publish the work as-is in a well-regarded but more tutorial journal such as ACS Catalysis, or to include these significant new efforts and resubmit to NatComm.

Reviewer #3 (Remarks to the Author):

In the present manuscript, authors describe an improved heterogeneous catalyst system for CO_2 hydrogenation to a series of hydrocarbons with high selectivity towards the jet fuel range. The low CO and CH_4 selectivity make this system promising. The article has been well edited and justifies publication after minor revisions:

The reviewer has the following comments/suggestions for minor revisions:

1. Authors should discuss more clearly the innovations, benefits as well as limitations of the novel catalyst system in comparison to the existing systems as summarized in Table 1.

2. Figure 1b should also have a plot for CO selectivity with time.

3. Figure 1d has a lot of data, hence should be represented more clearly or split into 2 plots.

3. It is interesting that the CH_4 selectivity follows a contrasting trend to that of light olefins with time. Authors should add some more insights into this observation.

4. Figure 1d shows that Fe-Cu-K shows higher selectivity for C5+ products than Fe-Mn-K, with similar CO_2 conversion. This means Fe-Cu-K is superior to Fe-Mn-K, contrary to authors' claim in Page 13 and throughout the text stating Fe-Mn-K as the most efficient among the screened catalysts. Authors should clarify how Fe-Mn-K is better than others in the text.

5. Authors have included a comprehensive section on the circular economy, as an outlook. However, various examples of circular economy, where CO_2 from air is directly captured and converted into value-added products have been published in the last few years, especially by Olah, Prakash and co-workers in producing methanol through a similar process as mentioned in this section. Hence, authors should mention these examples and add to the list of references for further reading.

Point-by-point response to the reviewers' comments

Black colour: the comments from reviewers;

Red colour: response to the comments from reviewers;

Purple colour: revised and new sentences in the revised manuscript.

Reviewer #1 (Remarks to the Author):

The manuscript deals with the application of the method of organic combustion for the synthesis of an efficient catalysts for CO2 hydrogenation directly to Jet fuel. The catalysts prepared by this way demonstrate high activity at high selectivity to C5+ hydrocarbons and low selectivity to methane and CO which is usually for these types of materials. This is an interesting study and I would recommend this manuscript for publication. However, there are several important points which have to be clarified:

We thank the reviewer for his/her comments that this is an interesting study and is recommended for publication but request that we attend to several important points. We also note the constructive comments suggested for such revisions and have carefully revised the manuscript as the reviewer has proposed.

1. It would be important to provide ASF distribution of the formed products to compare with existing catalysts. It seems that chain growth probability is relatively high which makes it similar to low temperature FT synthesis but with a high contribution of olefins.

We have now plotted the ASF distribution for CO_2 hydrogenation on a Fe-Mn-K catalyst. The chain growth is indeed high, with α_1 = 0.79 for the range from C_1-C_{12}, and α_2 = 0.57 for C_{12+}. This is therefore a double ASF product distribution. The following sentences were added to the main text.

"Similarly with FTS, the hydrocarbon products from CO_2 hydrogenation on Fe-Mn-K generally follow the ASF distribution. Figure 1d shows a double ASF product distribution [1], whose chain growth probabilities (α_i) is 0.79 for α_1 within the C_1-C_{12} carbon range and α_2 is 0.57 for C_{12+}(i.e., heavy hydrocarbons). A high chain growth probability (α_1) means a low methane selectivity whilst the chain growth decreases when the carbon number is above 12, indicating lower selectivity for higher (heavier) hydrocarbons."

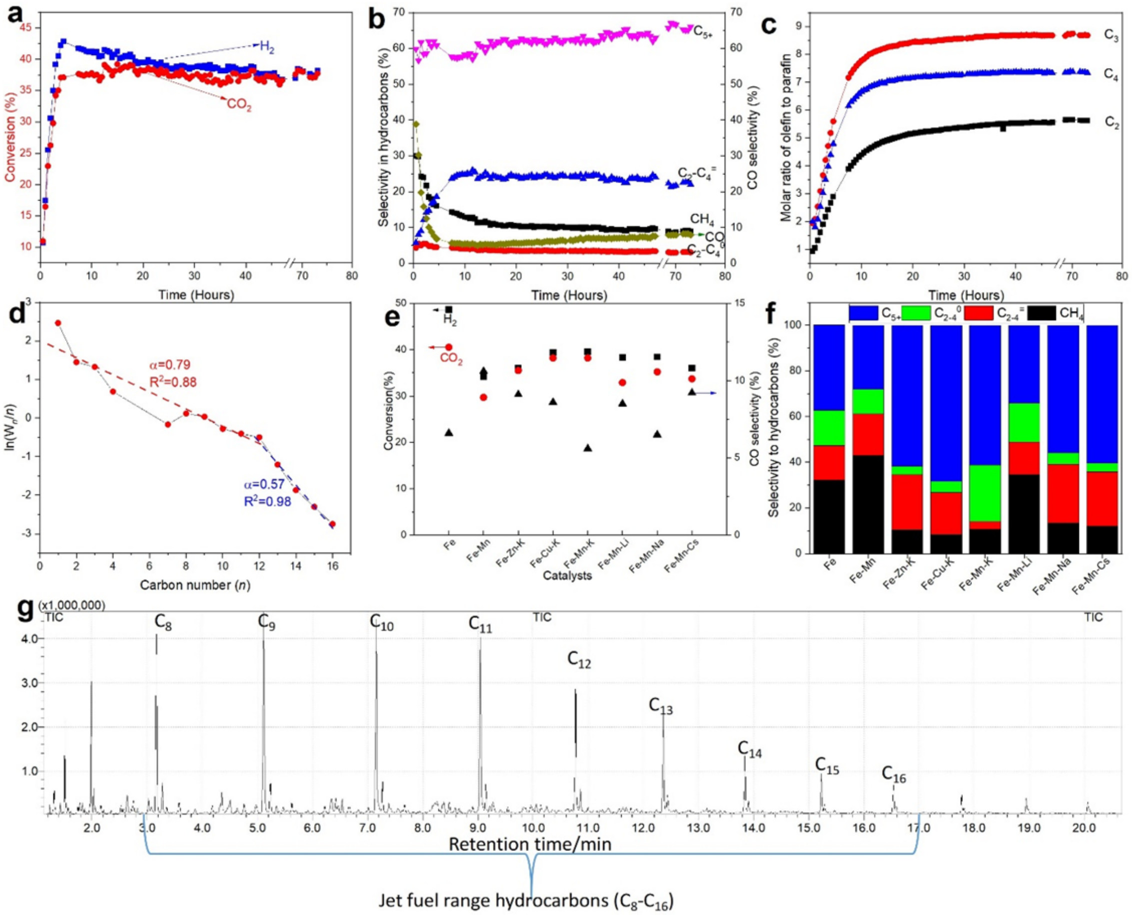

Fig. 1 Catalyst performance for the hydrogenation of carbon dioxide using a Fe-Mn-K catalyst. (**a**): % conversion of CO_2 and H_2 as a function of reaction time for the hydrogenation of CO_2. (**b**): Selectivity of various hydrocarbon products with reaction time for the hydrogenation of CO_2. (**c**): Molar ratio of olefin-to-paraffin for the C_2-C_4 range with reaction time for the hydrogenation of CO_2 (**d**): ASF plot and α values at reaction time of 20 hours. (**e**): Conversion and CO selectivity of CO_2 hydrogenation for a reaction time of 20 hours over different catalysts. (**f**): Products selectivities of CO_2 hydrogenation for a reaction time of 20 hours over different catalysts. (**g**): GC-MS spectrum of the hydrocarbon fuel from the hydrogenation of carbon dioxide on a Fe-Mn-K catalyst. The jet fuel range hydrocarbons (C_8 to C_{16}) are shown.

2. The catalyst Fe-Mn-K has been prepared by calcination at 350C. Probably there is still a small amount of carbon from the organic compounds which would explain higher selectivity to C5+ products. I would suggest perform TG analysis to check if the catalyst is carbon-free after this treatment.

Yes, an important point! We have therefore checked the Fe-Mn-K catalyst precursor (prepared with citric acid combustion method) using TGA analysis. The result revealed that, there is small amount of carbon residue (ca. 3.5 *wt* %) remained in the catalyst after calcination at 350 ^{o}C.

Previous work[2,3] has reported that the carbon materials can improve the olefin selectivity, and can also facilitate the formation of iron carbides during the activation, which is beneficial for the higher liquid products selectivity.

What's more, the C observed on the catalyst from the XPS spectra of the C 1s (Figure 2 f) can be divided 3 types; those around 40% C sp2 at binding energy peak of 284.7 eV, 15% C=O at binding energy peak of 288.4 eV, and 45% C sp3 at binding energy peak of 285.3 eV. The C sp2 is due to the carbon residue due to the calcination of citric acid, and peak of C=O and C sp3 can be attributed to the citric acid residue which has not fully decomposed.

Thus, we have amended our text and the new analysis is added accordingly.

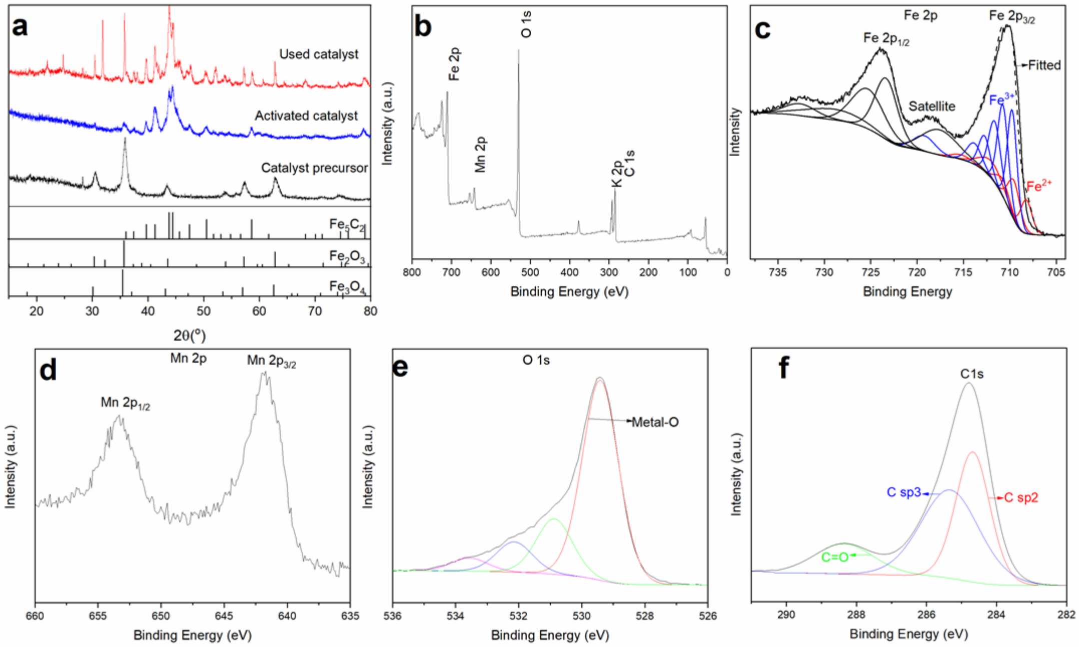

Fig. 2 XRD and XPS spectra of the Fe-Mn-K catalyst:

(**a**): Powder XRD spectra of the catalyst precursor and both the activated and the used catalyst. The corresponding JCPDS numbers are, for Fe_2O_3: 00-020-0508; χ-Fe_5C_2: 00-024-0081; Fe_3O_4: 03-065-3107; (**b**): XPS survey spectrum of the Fe-Mn-K catalyst; (**c**): High resolution XPS spectra in the region of the Fe 2p peak on the Fe-Mn-K catalyst; (**d**): XPS spectra of the Mn 2p on the Fe-Mn-K catalyst; (**e**): XPS spectra of the O 1s on the Fe-Mn-K catalyst; (**f**): XPS spectra of the C 1s on the Fe-Mn-K catalyst.

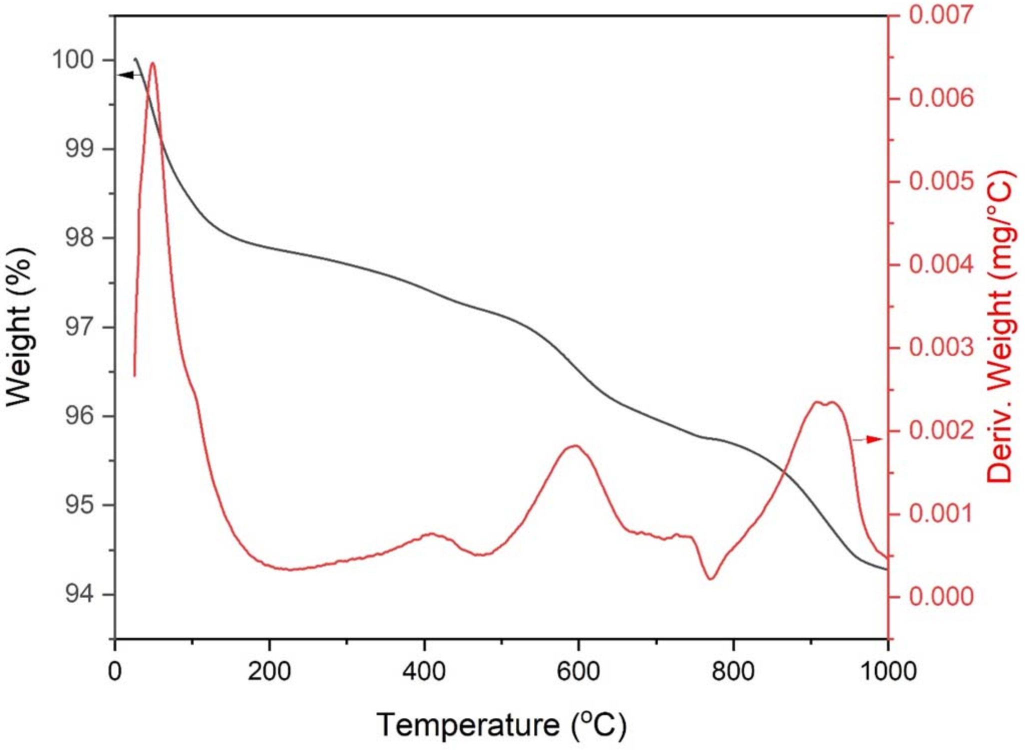

Figure S23 TGA results of catalyst precursor of Fe-Mn-K (citric acid method)

"The XPS spectra of the C 1s present (Figure 2 f) showed that around 40% of C sp2 at a characteristic binding energy peak of 284.7 eV; some 15% C=O at binding energy peak of 288.4 eV; and finally , 45% C sp3 at binding energy peak of 285.3 eV. The C sp2 is due to the carbon residue due to the calcination of citric acid, and the peak of C=O and C sp3 can be attributed to the citric acid residue which have not fully decomposed."

"Temperature-programmed oxidation (TPO) results of Fe-Mn-K catalyst precursor prepared with citric acid combustion method shown in Figure S24 revealed a small amount (about 3.5 *wt*%**)** of carbon residue in the after calcination at 350 ^{o}C. The presence of this small amount of carbon in the catalyst is reported to be beneficial for a higher olefin product selectivity**.** Thus, previous work[2,3] reported that the surrounding carbonaceous matter could facilitate the formation of iron carbides during activation, hence improving the higher liquid products selectivity."

The following sentences were also added in the Catalyst characterization section.

"Thermogravimetric analysis (TGA) was used to characterise the resulting carbon depositions in our catalyst samples. A temperature-programmed oxidation (TPO) was carried out to determine the thermal stability of the produced carbons. The sample was heated from room temperature to 1000 °C at a heating rate of 10 °C/min under an air atmosphere with a flow rate of 100 ml/min."

3. It is not clear what is the role of access to O2 during catalyst preparation and how authors controlled the temperature. There is no O2 in the proposed equation. In other case oxidation of Fe salt should proceed till Fe2O3. It would be important to give more details and comments on on this procedure.

Another important point: Thank You. At 350 ^{o}C the redox gel is combusted to produce Fe_3O_4 without any apparent O_2 participation from the atmosphere. However, we have noted that when the redox gel is combusted at 500 ^{o}C the main product is Fe_2O_3. The source of the oxygen on Fe_3O_4 could be the oxygens from the ligands, at this higher temperature the complexing ligands may break down further to give more O atoms.

The following sentences were therefore added in the main text for clarification.

"It is interesting that most of redox gel when combusted at a calcining temperature of 350 ^{o}C. produces Fe_3O_4 without any apparent O_2 participation from the atmosphere However, the main products are Fe_2O_3 when the calcination temperature increased to 500 ^{o}C, clearly as a consequence of the participation of atmospheric oxygen and / or the associated oxygens from the complexing ligands."

4. There is no clear correlation between the type of fuel, crystalline sizes and catalytic performance, however, small polyacids (tartaric acid, DTPA…) seem to have better activity and selectivity. It would be important to provide some vision about the efficiency and the role of fuels in this procedure. The possible explanation could be in higher intimacy between Fe, Mn and K in the catalysts prepared by OCM. Additional characterization to clarify the interaction between components would be highly desirable here.

We thank the reviewer for these important observations on the submitted text and various suggestions to improve the manuscript . In response, the following sentences were added to the main text.

"In general, the Fe-Mn-K catalysts synthesised with carboxylic acids and polycarboxylic acids as fuels showed superior catalytic performances than those prepared using urea and sugar (glucose) and the catalyst prepared without fuel. Our assertion is that this trend probably derives from two crucial roles (i.e., both a chelating agent and fuel) that these organic molecules play in the organic combustion approach. The first role can enhance the homogeneity of the solution through the intimacy between the constituent metal (Fe, Mn, K) precursors, hence hindering their precipitation or aggregation during the gel formation, whilst the second (fuel) function can closely control the severity of the combustion reaction and hence the aggregation of the nanostructured catalysts. Obviously, this leads to changes in the crystallite sizes that show the Fe-Mn-K catalysts with particle sizes between 7 and 28 nm and usualy prepared by carboxylic acid-type fuels are significantly more active and selective than the catalysts with larger crystallite sizes (i.e., 56-74 nm)."

5. The authors provide the mechanism of transformation Fe3O4, carbide and Fe2O3 to each other, however, there is no clear evidence in manuscript about it. I would recommend to perform model reactions to confirm this mechanism. For example, by treatment of the catalyst by CO2 demonstrate formation of Fe2O3 from Fe3O4 and reduction of Fe2O3 by H2 to show formation of Fe3O4.

Many thanks! The model experiments relating to CO_2 oxidation of Fe_3O_4 to Fe_2O_3 and H_2 reduction of Fe_2O_3 to Fe_3O_4 have therefore been carried out at a temperature of 350 ^oC for 16 hours. The experimental results confirmed that the Fe_3O_4 can be converted to Fe_2O_3 under a CO_2 atmosphere and Fe_2O_3 was reduced as Fe_3O_4 under a H_2 atmosphere. The XRD spectrum is shown in Figure S24 and the following sentence is revised in the main text:

"In model experiments, Fe_2O_3 was produced from the oxidation of Fe_3O_4 by CO_2/H_2O, and Fe_2O_3 was steadily reduced to Fe_3O_4 by H_2 in the reaction system (Figure S23)."

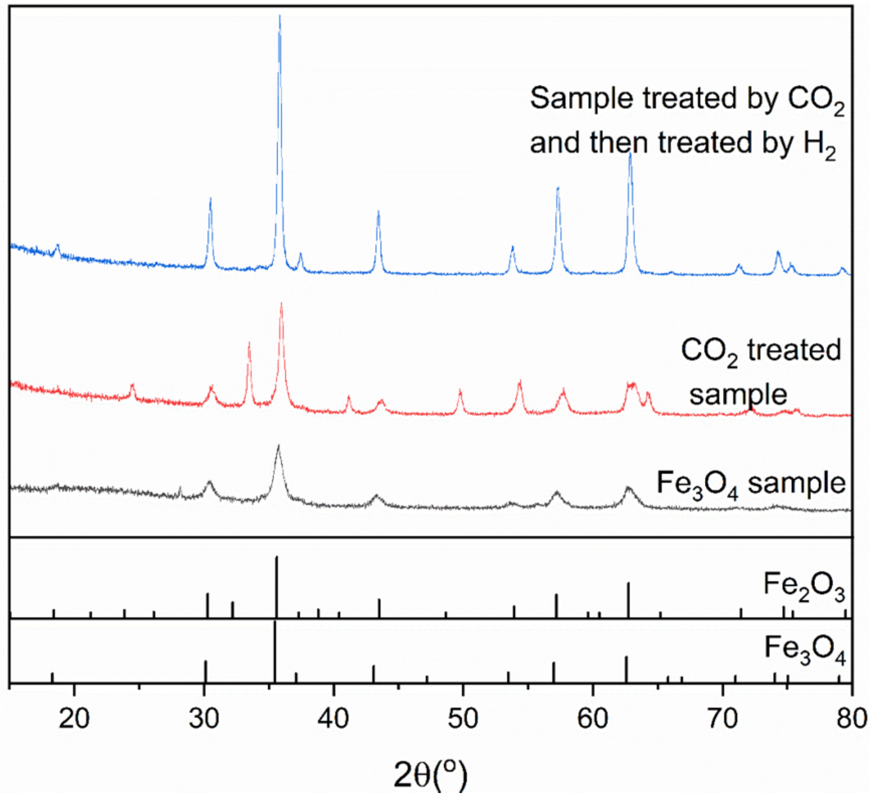

Figure S24 XRD spectrum of Fe_3O_4 (black), sample treated in a CO_2 atmosphere 350 ^oC for 16 hours (red) and sample treated in a CO_2 atmosphere and then treated in a 5%H_2/N_2 atmosphere at 350 ^oC for 16 hours respectively (blue)

Reviewer #2 (Remarks to the Author):

The authors present a preparation procedure for catalysts with relatively high yield to higher hydrocarbons (jet fuel) from CO2. In my opinion, this is solid and interesting work. However, I am not sure that this work provides truly novel insights, especially as the characterization/explanation for the observed trends is meager.

We thank the reviewer for considering our manuscript "solid and interesting". However, we do oppose his/ her views and strongly believe that our work does indeed provide novel insights and significant progresses into the CO_2 hydrogenation reaction. Thus, we outline, several important advances that can be extracted from this work:

1. We show for the first time the clear advantages that a relatively new catalyst preparation method (Organic Combustion Method) bring to the production of renewable jet fuel through the CO_2 hydrogenation using a cheap and abundant Fe-based catalyst. This one-pot catalyst synthesis is markedly simpler and cheaper than that (Na–Fe_3O_4 /HZSM-5) reported for converting CO_2 into a gasoline fuel [J. Wei et al., Directly converting CO_2 into a gasoline fuel, Nat. Comm., 8:15174 | DOI: 10.1038/ncomms15174].[4]
2. We have found strong evidence that reveals the unprecedented dual effect of the complexing character and the control of the combustion reaction of the organic fuel to tune and control the nanoparticle size and hence the catalytic performance of Fe-Mn-K catalyst toward jet fuel.
3. We have been able to elucidate a tandem mechanism operating on two different Fe phases (i.e., Fe_3O_4 and χ-Fe_5C_2). Thus, Magnetite efficiently converts CO_2 to CO via RWGS reaction whilst the Hägg iron carbide (χ-Fe_5C_2) catalyses the F-T reaction.

We have also enlarged the discussion of our data to rationalise the effects of the fuel in OC method over the different crystallite phases and their influences over the products distribution by adding the below text:

"It is interesting that most of redox gel when combusted at a calcining temperature of 350 ^{o}C. produces Fe_3O_4 without any apparent O_2 participation from the atmosphere However, the main products are Fe_2O_3 when the calcination temperature increased to 500 ^{o}C, clearly as a consequence of the participation of atmospheric oxygen and / or the associated oxygens from the complexing ligands

"In general, the Fe-Mn-K catalysts synthesised with carboxylic acids and polycarboxylic acids as fuels showed superior catalytic performances than those prepared using urea and sugar (glucose) and the catalyst prepared without fuel. This trend probably derives from the two crucial roles (i.e., both a chelating agent and fuel) that these organic molecules play in the organic combustion approach. The first role can enhance the homogeneity of the solution through the intimacy between the constituent metal (Fe, Mn, K) precursors, hence hindering their precipitation or aggregation during the gel formation, whilst the second (fuel) function can closely control the severity of the combustion reaction and hence the aggregation of the nanostructured catalysts. Obviously, this leads to changes in the crystallite sizes that show the Fe-Mn-K catalysts with particle sizes between 7 and 28 nm and usually prepared by carboxylic acid-type fuels are significantly more active and selective than the catalysts with larger crystallite sizes (i.e., 56-74 nm)."

"In order to investigate the fundamental, underlying efforts of organic fuel compounds, the catalyst prepared without fuel also been synthesised. It is clear that the catalyst prepared without fuel showed lower catalytic activity (CO_2 conversion of 28.6%) compared with the catalyst prepared with citric acid (CO_2 conversion of 38.2%) "

Furthermore, I miss a rationale in the flow of the story for why this catalyst would work after the introduction (which is an excellent introduction) that most catalysts don't work to this extent to convert CO2 to jet fuels, before the current results. Even though the catalyst

preparation procedure is explained in the methods and materials, a quick introduction and rationale should be included before the results are delved into.

Thank you for this important comment. Yes, we understand and agree on reflection.The following sentences were therefore added in the main text to attempt to confront this important point : .

"The rising concerns over climate change and the stringent environmental regulations to deplete the utilization of fossil-derived fuels have generated great opportunities on the transformation of CO_2 into sustainable, synthetic hydrocarbons fuels, particularly in the synthesis of renewable aviation fuels. The progress of this catalytic process is closely related to the development of advanced catalysts of high performance for the CO_2 hydrogenation reaction. Therefore, the utilization of novel methods of catalyst preparation represents an important strategy to produce advanced catalytic formulations having high performance levels. Among the catalyst synthesis methods, the so-called organic combustion method is an energy-efficient and economically viable approach for the one-pot synthesis of a variety of nanostructured solid catalysts. In this method, the utilization of an organic fuel, having also a complexation character, to yield a homogenous redox solution of the different metal precursors is highly advantageous. In addition, a relatively moderate self-sustained exothermic reaction of the redox gel may be beneficial to produce the necessary nanostructured catalysts with an efficient promoter effect due to the well-controlled aqueous chemistry of the preparation route and combustion conditions."

I am also wondering about the carbide phase formed and its contribution to the products. I would like to see isotopic labelling and an understanding of the contribution of Mars van Krevelen type mechanisms.

Thank you for your request about the carbide phase formation and contribution to the products. We included in the main text,

"For the iron□based catalysts in FTS, it is widely accepted that the active catalytic phase is Hägg carbide (χ□Fe_5C_2). A typical method for preparing Hägg carbide is the reduction and subsequent carburization of hematite in FTS reaction conditions (lean H_2 syngas at ≈200–450 °C). The starting material, hematite, is gradually reduced and then carburized to Hägg carbide in the following sequence: hematite (Fe_2O_3)→magnetite (Fe_3O_4)→wüstite (FeO)→iron metal (Fe)→Hägg carbide (χ□Fe_5C_2).[5] In our experiments, the χ□Fe_5C_2 was reduced and carburized from Fe_3O_4, in the process of magnetite (Fe_3O_4)→wüstite (FeO)→iron metal (Fe)→Hägg carbide (χ□Fe_5C_2)."

"The carbide phase detected by powder-XRD diffraction was χ-Fe_5C_2 which plays a principal role in the formation of hydrocarbons via FT reaction [6-9]. According to the literature and our results, the carburization process of Fe nanoparticles during the catalytic reaction forms the Fe carbide phase, which through a FT pathway favours the C-C condensation reactions to produce large hydrocarbons within the range of aviation fuel"

"In our experiments, the χ-Fe_5C_2 was formed during the catalyst activation/reduction process, in the beginning of the reaction what it is happening is mainly CO_2 methanation reaction on χ-Fe_5C_2, the relatively high pressure of water can then oxidize on χ-Fe_5C_2 to Fe_3O_4, and the Fe_3O_4 was simultaneously carburized by CO."

We do appreciate very much your suggestion of gaining further insight into the contribution - or not - of Mars van Krevelen type mechanisms in the Hydrogenation of CO_2 into Jet Fuel on Fe-Mn-K catalysts. We are seriously considering this type of isotopic study for future work but would find it difficult at present to confront this issue directly due to the practicalities of doing this . Thus, as the Reviewer will be aware, the isotopic study would have to be carried out in a sealed system as a flow – through configuration (that is, our present set-up) is highly financially impracticable. In addition, as we are sure you are aware, gas residence times and kinetics will be entirely different and will have little direct relevance to the reported system. In a flowing gas system these will clearly be experimentally – and financially (!) - challenging. We hope that this satisfies your concerns

Nevertheless, we have included in the manuscript the following text to state that this type of mechanism is of course worthy of future detailed consideration.

"Using iron-based catalysts for FT synthesis a fast and reversible exchange of Fe_3O_4 to Fe_xC_y carbides and vice versa can occur under appropriate reaction conditions. This relatively facile and reversible phase transformation makes possible the incorporation of carbon atoms from the carbide surface into the reaction products via Mars-van Krevelen mechanism as was determined by Gracia *et al.*[10] through a computational study of the CO hydrogenation on an iron carbide surface. Remarkably, this Mars-van Krevelen-like mechanism on supported Fe catalysts rationalised the enhanced reactivity of highly dispersed iron carbide particles in the initiation of chain growth in F-T synthesis[11]**.**

As far as we know, there is not a single report in the scientific literature of the Mars-van Krevelen mechanism operating in the CO_2 hydrogenation reaction on Fe catalysts. Obviously, this reaction is more challenging than conventional FT synthesis since the catalyst must have an excellent balance of active sites (phases) to catalyse - in tandem mode- the reverse-water gas shift reaction (or CO_2 partial hydrogenation) and also the CO hydrogenation via the FT reaction to produce Jet Fuel. Our tandem mechanism through the participation of Fe_3O_4 and χ-Fe_5C_2 can easily rationalise the jet fuel formation and give a wider picture of the evolution of the gas, liquid and solid phases during the catalytic reaction. Further work is needed to gain further insight into the possible occurrence of Mars-van Krevelen-like mechanism in the FT stage through carbon isotopic labelling studies. In a flowing gas system these will clearly be experimentally – and financially (!) - challenging. "

XRD - Claim that Mn and K are not observed and are thus highly disperse simply does not hold true. There can be clusters of up to 2 nm of pure material and still be no reflections due to the periodicity needed in XRD. It is best to verify such claims with methods that are more sensitive, for example simple XAS measurements which might be relatively easy to obtain given the affiliations.

Thank you for such an important point. Effectively, it is not strictly correct as you point out the statement that Mn and K are highly dispersed based on the powder XRD analysis because the reflections are associated to the phase periodicity or crystallinity. Unfortunately, we do not have direct access to X-ray absorption spectroscopy (XAS) analysis at this juncture to provide very valuable information on the formal oxidation state and local coordination environment of the promoters and Fe. This must be carried out in a future study However, we

have carried out the XPS analysis, a very valuable surface technique, and the survey spectrum (Figure 2b) clearly indicates that the sample contains Fe, Mn, K, O and C. We have added the XPS spectra of Mn 2p, O 1s and C 1s, and the following sentence was added to the main text.

"In Figure 2d we show the Mn 2p XPS spectra, which displayed a spin-orbit doublet of Mn 2p3/2 and Mn 2p1/2 peaks with a binding energy gap of 11.6 eV can be assigned to Mn_2O_3. In addition, in Figure 2e we show the O 1s, XPS spectra with a main peak at 529.4 eV, clearly originating from the presence of metal-O bonds."

"The XPS spectra of the C 1s (Figure 2 f) showed that the around 40% C sp2 at binding energy peak of 284.7 eV, 15% C=O at binding energy peak of 288.4 eV, and 45% C sp3 at binding energy peak of 285.3 eV. The C sp2 is due to the carbon residue due to the calcination of citric acid, and peak of C=O and C sp3 can be the citric acid residue which have not fully decomposed."

Generally speaking, some attention should be given to the figures which could be more attractive/self explanatory (not a scientific comment per se but it may help the authors to better structure the story as well).
Thank you for this valid and highly important comment. We have regrouped some figures (Figure 1 and Figure 2) and highlighted the text in the figures to hopefully make the images more attractive.

Blank experiments with a typical catalyst preparation procedure should also be included to compare the used setup/materials to the available literature.

Thank you- understood – and appreciated ! . We have indeed now carried out a blank test, which corresponds to the catalyst formulation without organic fuel. The catalyst prepared without organic fuel showed lower catalytic activity, with only 28.6% CO_2 conversion. This preparation can also be seen as an uncontrolled precipitation method since the aqueous solution-containing metal precursors were dried to produce a slurry and then calcined to produce the catalyst precursor.
We include it in the main text.

"In order to investigate the fundamental, underlying efforts of organic fuel compounds, the catalyst prepared without fuel also been synthesised. It is clear that the catalyst prepared without fuel showed lower catalytic activity (CO_2 conversion of 28.6%) compared with the catalyst prepared with citric acid (CO_2 conversion of 38.2%)."

The proposed reaction scheme is not based on much, something like in-situ spectroscopy is missing, or at the very least the proposed isotopic labelling experiments.

Thank you for your interest in understanding the reaction mechanism; yes, we completely see your point. We note above the challenges of an isotopic substitution set of experiments

Even in the absence of such isotopic experiments, we had thought that we had proposed this reaction scheme based on well-established chemical reactions and supported by the characterization data of the Fe catalysts both before and after the catalytic reaction, the

product distribution of the catalytic tests and a well-documented report in the literature [**[J. Wei et al., Directly converting CO_2 into a gasoline fuel, Nat. Comm., (2017) 8:15174 | DOI: 10.1038/ncomms15174]**.[4] Obviously, there is always room to extend our investigations and we expect to do it in the future to gain further understanding of the mechanism of this very exciting catalytic reaction.

In order to give further support to our mechanism proposal, we have carried out additional experiments for the CO_2 oxidation of Fe_3O_4 to Fe_2O_3 and H_2 reduction of Fe_2O_3 to Fe_3O_4 at 350 ^oC for 16 hours, The experimental results confirmed that the Fe_3O_4 can be converted to Fe_2O_3 under CO_2 atmospheric, and Fe_2O_3 was reduced as Fe_3O_4 under H_2 atmospheric. The XRD spectrum is shown in Figure S24 and the following sentence was included in the main text:

"In model experiments, Fe_2O_3 was produced from the oxidation of Fe_3O_4 by CO_2/H_2O, and Fe_2O_3 was steadily reduced to Fe_3O_4 by H_2 in the reaction system (Figure S23)."

I realize the experiments I suggest may require some significant effort, and also realize that the work can be seen as currently complete. Thus it is, in my opinion, up to the authors to decide to publish the work as-is in a well-regarded but more tutorial journal such as ACS Catalysis, or to include these significant new efforts and resubmit to NatComm.

We appreciate this comment and understand some of the sentiments but we do believe that our work with the current corrections and additions is now in a very strong position to meet (or even exceed) the level of discussion of CO_2 hydrogenation–related papers recently published in Nature Communication. A selection of these is given here for interest and comparison

[R. Ye et al., CO_2 hydrogenation to high-value products via heterogeneous catalysis, Nat. Comm., (2019) 10:5698 |https://doi.org/10.1038/s41467-019-13638-9;[12] L. Wang et al., Silica accelerates the selective hydrogenation of CO_2 to methanol on cobalt catalysts, Nat. Comm., (2020) 11:1033 |https://doi.org/10.1038/s41467-020-14817-9;[13] C. Vogt et al., Understanding carbon dioxide activation and carbon–carbon coupling over nickel, Nat. Comm., (2019) 10:5330 |https://doi.org/10.1038/s41467-019-12858-3].[14]

We have included further TGA, XRD and XPS results that certainly support our advance and boost this work.

Reviewer #3 (Remarks to the Author):

In the present manuscript, authors describe an improved heterogeneous catalyst system for CO2 hydrogenation to a series of hydrocarbons with high selectivity towards the jet fuel range. The low CO and CH4 selectivity make this system promising. The article has been well edited and justifies publication after minor revisions:

We thank the reviewer for his / her comment … "well edited and justifies publication after minor revisions" and useful suggestions for those minor revisions. We have revised our manuscript as the reviewer suggested.

The reviewer has the following comments/suggestions for minor revisions:

1. Authors should discuss more clearly the innovations, benefits as well as limitations of the novel catalyst system in comparison to the existing systems as summarized in Table 1.

Compared with the other systems in Table 1, our catalyst showed both high CO_2 conversion and liquid product selectivity, especially its selectivity for jet fuel range hydrocarbons. Our catalyst preparation method also showed advantages in energy and time saving.

However, in an attempt to respond positively with additional text, we have added the following:

"Compared to the co-precipitation method, widely applied in the preparation of Fe-based catalysts, we show that the OCM is a particularly facile production process where, in addition to high yields and selectivity for jet fuels, additional advantages are savings in both energy and time."

We also added a further sentence to the main text:

"Compared with the experimental results in Table 1, the prepared Fe-Mn-K catalyst showed higher liquid products (C_{5+}) yield, with the catalyst presenting both high CO_2 conversion and high C_{5+} selectivity."

2. Figure 1b should also have a plot for CO selectivity with time.

Many thanks! The curve for CO selectivity has been added in the new version of Figure 1b.

3. Figure 1d has a lot of data, hence should be represented more clearly or split into 2 plots.

Figure 1d has been split into two plots (Figure 1 e and Figure 1 f in the new version) as the reviewer suggested.

3. It is interesting that the CH4 selectivity follows a contrasting trend to that of light olefins with time. Authors should add some more insights into this observation.

We have noticed high CH_4 selectivity in the beginning, which then decreased with reaction time. This is because at the beginning of the reaction what it is happening is mainly CO_2 methanation reaction on χ-Fe_5C_2, the relatively high pressure of water can then oxidize χ-Fe_5C_2 to Fe_3O_4, and the Fe_3O_4 was simultaneously carburized by CO. χ-Fe_5C_2 and Fe_3O_4 are the active sites for FTS and RWGS reactions, respectively.

The following sentences were added in the reaction scheme section.

"Interestingly, the methane selectivity decreased dramatically at the beginning of the reaction due to the main reaction being CO_2 methanation over the catalyst active sites (χ-Fe_5C_2). The produced a high pressure of water and unconverted CO_2 which can then oxidize χ-Fe_5C_2 to Fe_3O_4. The CO produced via the reverse water gas shift reaction on Fe_3O_4 active site reacts with H_2 (Fisher-Tropsch synthesis), and the CO_2 conversion increased rapidly (Figure 1a). The product selectivity was then stable after a reaction time of 10 hours."

4. Figure 1d shows that Fe-Cu-K shows higher selectivity for C5+ products than Fe-Mn-K,

with similar CO2 conversion. This means Fe-Cu-K is superior to Fe-Mn-K, contrary to authors' claim in Page 13 and throughout the text stating Fe-Mn-K as the most efficient among the screened catalysts. Authors should clarify how Fe-Mn-K is better than others in the text.

Yes, the C_{5+} selectivity of Fe-Cu-K (68%) is a little higher than Fe-Mn-K (62%), and, the CO_2 conversion between Fe-Cu-K and Fe-Mn-K are similar. Actually, there is no great difference between the catalysts Fe-Mn-K, Fe-Cu-K, Fe-Zn-K, and even Fe-Mn-Na, Fe-Mn-Cs. However, if we focus on the selectivity of jet fuel range hydrocarbons (C_8-C_{16}), Fe-Mn-K catalyst showed highest selectivity (47.8%). The selectivity of jet fuel range hydrocarbons were added in the modified Figure 1f and the following sentences were modified in the main text:

There was no significant difference between the performances of these three catalysts, although the Fe-Mn-K catalyst showed slightly better selectivity for jet fuel synthesis.

Was revised as:

"There was no significant difference between the performances of these three catalysts, but the Fe-Mn-K catalyst showed slightly better selectivity for jet fuels synthesis (47.8%) than catalysts of Fe-Cu-K (40.8%) and Fe-Zn-K (45.1%)."

"The Fe-Mn-K catalyst showed slightly better performance for carbon dioxide conversion and target product selectivity compared to the Fe-Mn-Na and Fe-Mn-Cs catalysts.

Was revised as:

"There are no large differences in the catalytic performance between the catalysts Fe-Mn-K, Fe-Mn-Na, and Fe-Mn-Cs. However, the Fe-Mn-K catalyst showed higher C_8-C_{16} selectivity (47.8%) than Fe-Mn- Na (44.4%) or Fe-Mn-Cs (44.0%)."

5. Authors have included a comprehensive section on the circular economy, as an outlook. However, various examples of circular economy, where CO2 from air is directly captured and converted into value-added products have been published in the last few years, especially by Olah, Prakash and co-workers in producing methanol through a similar process as mentioned in this section. Hence, authors should mention these examples and add to the list of references for further reading.
Thank you for your suggestions. We have added more papers (ref 15-18) as references. We have always been inspired by the advances of Olah, Prakash and colleagues, and indeed others, and we hope that these additions pay due-credit to their remarkable and inspirational ideas

"Nowadays, researchers have advanced the concept of the so-called CO_2 Circular Economy, which integrates directly capture CO_2 from air (DCA) and converted CO_2 into value-added products[15-18]. This Circular Economy is a valid and highly powerful alternative route to simply burying captured CO_2 underground and one in which future generations will surely expect us to form a major aspect of sustainable CO_2 management."

Goeppert, A., Czaun, M., Jones, J.-P., Prakash, G. S. & Olah, G. A. Recycling of carbon dioxide to methanol and derived products–closing the loop. *Chemical Society Reviews* **43**, 7995-8048 (2014).

Nocito, F. & Dibenedetto, A. Atmospheric CO_2 mitigation technologies: carbon capture utilization and storage. *Current Opinion in Green and Sustainable Chemistry* **21**, 34-43, (2020).

Ampelli, C., Perathoner, S. & Centi, G. CO_2 utilization: an enabling element to move to a resource-and energy-efficient chemical and fuel production. *Philosophical Transactions of the Royal Society A: Mathematical, Physical Engineering Sciences* **373**, 20140177 (2015).
Falcinelli, S. Fuel production from waste CO_2 using renewable energies. *Catalysis Today* **348**, 95-101, (2020).

References:

1 Nakhaei Pour, A., Khodabandeh, H., Izadyar, M. & Housaindokht, M. R. Mechanistic double ASF product distribution study of Fischer–Tropsch synthesis on precipitated iron catalyst. *Journal of Natural Gas Science and Engineering* **15**, 53-58, (2013).
2 Bahome, M. C., Jewell, L. L., Hildebrandt, D., Glasser, D. & Coville, N. J. Fischer–Tropsch synthesis over iron catalysts supported on carbon nanotubes. *Applied Catalysis A: General* **287**, 60-67 (2005).
3 Lu, Y. *et al.* Fischer–Tropsch synthesis of olefin-rich liquid hydrocarbons from biomass-derived syngas over carbon-encapsulated iron carbide/iron nanoparticles catalyst. *Fuel* **193**, 369-384 (2017).
4 Wei, J. *et al.* Directly converting CO_2 into a gasoline fuel. *Nature Communications* **8**, 15174, doi:10.1038/ncomms15174 (2017).
5 Abbaslou, R. M. M., Tavassoli, A., Soltan, J. & Dalai, A. K. Iron catalysts supported on carbon nanotubes for Fischer–Tropsch synthesis: Effect of catalytic site position. *Applied Catalysis A: General* **367**, 47-52, doi:10.1016/j.apcata.2009.07.025 (2009).
6 Yang, C., Zhao, H., Hou, Y. & Ma, D. Fe5C2 nanoparticles: a facile bromide-induced synthesis and as an active phase for Fischer–Tropsch synthesis. *Journal of the American Chemical Society* **134**, 15814-15821 (2012).
7 Liu, B. *et al.* Unravelling the New Roles of Na and Mn Promoter in CO2 Hydrogenation over Fe3O4 ‐ Based Catalysts for Enhanced Selectivity to Light α ‐ Olefins. *ChemCatChem* **10**, 4718-4732 (2018).
8 Liu, J. *et al.* Direct transformation of carbon dioxide to value-added hydrocarbons by physical mixtures of Fe5C2 and K-modified Al2O3. *Industrial Engineering Chemistry Research* **57**, 9120-9126 (2018).
9 Zhai, P. *et al.* Highly tunable selectivity for syngas ‐ derived alkenes over zinc and sodium ‐ modulated Fe5C2 catalyst. *Angewandte Chemie International Edition* **55**, 9902-9907 (2016).
10 Gracia, J. M., Prinsloo, F. F. & Niemantsverdriet, J. W. Mars-van Krevelen-like Mechanism of CO Hydrogenation on an Iron Carbide Surface. *Catalysis Letters* **133**, 257, (2009).
11 Ordomsky, V., Legras, B., Cheng, K., Paul, S. & Khodakov, A. The role of carbon atoms of supported iron carbides in Fischer–Tropsch synthesis. *Catalysis Science & Technology* **5**, 1433-1437 (2015).
12 Ye, R.-P. *et al.* CO_2 hydrogenation to high-value products via heterogeneous catalysis. *Nature Communications* **10**, 5698, doi:10.1038/s41467-019-13638-9 (2019).
13 Wang, L. *et al.* Silica accelerates the selective hydrogenation of CO_2 to methanol on cobalt catalysts. *Nature Communications* **11**, 1033, doi:10.1038/s41467-020-14817-9 (2020).
14 Vogt, C. *et al.* Understanding carbon dioxide activation and carbon–carbon coupling over nickel. *Nature Communications* **10**, 5330, doi:10.1038/s41467-019-12858-3 (2019).
15 Goeppert, A., Czaun, M., Jones, J.-P., Prakash, G. S. & Olah, G. A. Recycling of carbon dioxide to methanol and derived products–closing the loop. *Chemical Society Reviews* **43**, 7995-8048 (2014).

16 Nocito, F. & Dibenedetto, A. Atmospheric CO_2 mitigation technologies: carbon capture utilization and storage. *Current Opinion in Green and Sustainable Chemistry* **21**, 34-43, (2020).
17 Ampelli, C., Perathoner, S. & Centi, G. CO_2 utilization: an enabling element to move to a resource-and energy-efficient chemical and fuel production. *Philosophical Transactions of the Royal Society A: Mathematical, Physical Engineering Sciences* **373**, 20140177 (2015).
18 Falcinelli, S. Fuel production from waste CO_2 using renewable energies. *Catalysis Today* **348**, 95-101, (2020).

Reviewer #1 (Remarks to the Author):

The authors took into account all my comments and corrected the manuscript accordingly. The manuscript has been significantly improved and now the effect of organic combustion procedure is more clear. I would recommend it for publication.

Reviewer #2 (Remarks to the Author):

I can agree with most of the answers from the authors, and the manuscript has in my eyes sufficiently improved for publication with some minor revisions:

Some of the references that are crucial in the point-by-point response to the referees are not in the manuscript. They are evidently important to the work as the authors mentioned them and should therefor be included in the referee list. E.g. C. Vogt et al.,
Understanding carbon dioxide activation and carbon–carbon coupling over nickel, Nat.
Comm., (2019) 10:5330 |https://doi.org/10.1038/s41467-019-12858-3].

The figures have improved. Yet the authors should adjust the legibility of font size in the new figure schemes 1 and 2. The font size is too small, lines are also quite thin. These are greatly important figures to the text.

Point-by-point response to the reviewers' comments

Black colour: the comments from reviewers;

Red colour: response to the comments from reviewers;

REVIEWERS' COMMENTS

Reviewer #1 (Remarks to the Author):

The authors took into account all my comments and corrected the manuscript accordingly. The manuscript has been significantly improved and now the effect of organic combustion procedure is more clear. I would recommend it for publication.

We thank the reviewer for his/her comments and recommend for publication. His/her comments have helped us to improve our manuscript. Many thanks!

Reviewer #2 (Remarks to the Author):

I can agree with most of the answers from the authors, and the manuscript has in my eyes sufficiently improved for publication with some minor revisions:

We thank the reviewer for his/her comment of manuscript has sufficiently improved and suggestion of publication with some minor revisions.

Some of the references that are crucial in the point-by-point response to the referees are not in the manuscript. They are evidently important to the work as the authors mentioned them and should therefor be included in the referee list. E.g. C. Vogt et al.,
Understanding carbon dioxide activation and carbon–carbon coupling over nickel, Nat. Comm., (2019) 10:5330 |https://doi.org/10.1038/s41467-019-12858-3].

Many thanks! Yes, the references has been added:

Vogt, C. *et al.* Understanding carbon dioxide activation and carbon–carbon coupling over nickel. *Nature Communications* **10**, 5330, (2019).

Wang, L. *et al.* Silica accelerates the selective hydrogenation of CO_2 to methanol on cobalt catalysts. *Nature Communications* **11**, 1033, (2020).

What's more, we also added the references in the previous revision as below:

Ye, R.-P. *et al.* CO_2 hydrogenation to high-value products via heterogeneous catalysis. *Nature Communications* **10**, 5698, (2019).

Nakhaei Pour, A., Khodabandeh, H., Izadyar, M. & Housaindokht, M. R. Mechanistic double ASF product distribution study of Fischer–Tropsch synthesis on precipitated iron catalyst. *Journal of Natural Gas Science and Engineering* **15**, 53-58, (2013).

Bahome, M. C., Jewell, L. L., Hildebrandt, D., Glasser, D. & Coville, N. J. Fischer–Tropsch synthesis over iron catalysts supported on carbon nanotubes. *Applied Catalysis A:General* **287**, 60-67 (2005).

Lu, Y. *et al.* Fischer–Tropsch synthesis of olefin-rich liquid hydrocarbons from biomass-derived syngas over carbon-encapsulated iron carbide/iron nanoparticles catalyst. *Fuel* **193**, 369-384 (2017).

Abbaslou, R. M. M., Tavassoli, A., Soltan, J. & Dalai, A. K. Iron catalysts supported on carbon nanotubes for Fischer–Tropsch synthesis: Effect of catalytic site position. *Applied Catalysis A: General* **367**, 47-52, (2009).

Gracia, J. M., Prinsloo, F. F. & Niemantsverdriet, J. W. Mars-van Krevelen-like Mechanism of CO Hydrogenation on an Iron Carbide Surface. *Catalysis Letters* **133**, 257, (2009).

Ordomsky, V., Legras, B., Cheng, K., Paul, S. & Khodakov, A. The role of carbon atoms of supported iron carbides in Fischer–Tropsch synthesis. *Catalysis Science & Technology* **5**, 1433-1437 (2015).

Kirchner, J., Baysal, Z. & Kureti, S. Activity and Structural Changes of Fe-based Catalysts during CO_2 Hydrogenation towards CH_4–A Mini Review. *ChemCatChem* **12**, 981-988 (2020).

Goeppert, A., Czaun, M., Jones, J.-P., Prakash, G. S. & Olah, G. A. Recycling of carbon dioxide to methanol and derived products–closing the loop. *Chemical Society Reviews* **43**, 7995-8048 (2014).

Nocito, F. & Dibenedetto, A. Atmospheric CO_2 mitigation technologies: carbon capture utilization and storage. *Current Opinion in Green and Sustainable Chemistry* **21**, 34-43, (2020).

Ampelli, C., Perathoner, S. & Centi, G. CO_2 utilization: an enabling element to move to a resource-and energy-efficient chemical and fuel production. *Philosophical Transactions of the Royal Society A: Mathematical, Physical Engineering Sciences* **373**, 20140177 (2015).

Falcinelli, S. Fuel production from waste CO_2 using renewable energies. *Catalysis Today* **348**, 95-101, (2020).

The figures have improved. Yet the authors should adjust the legibility of font size in the new figure schemes 1 and 2. The font size is too small, lines are also quite thin. These are greatly important figures to the text.

Many thanks for the suggestions. We have adjust the font size and line thickness in the new Figure 1 and 2. As followed:

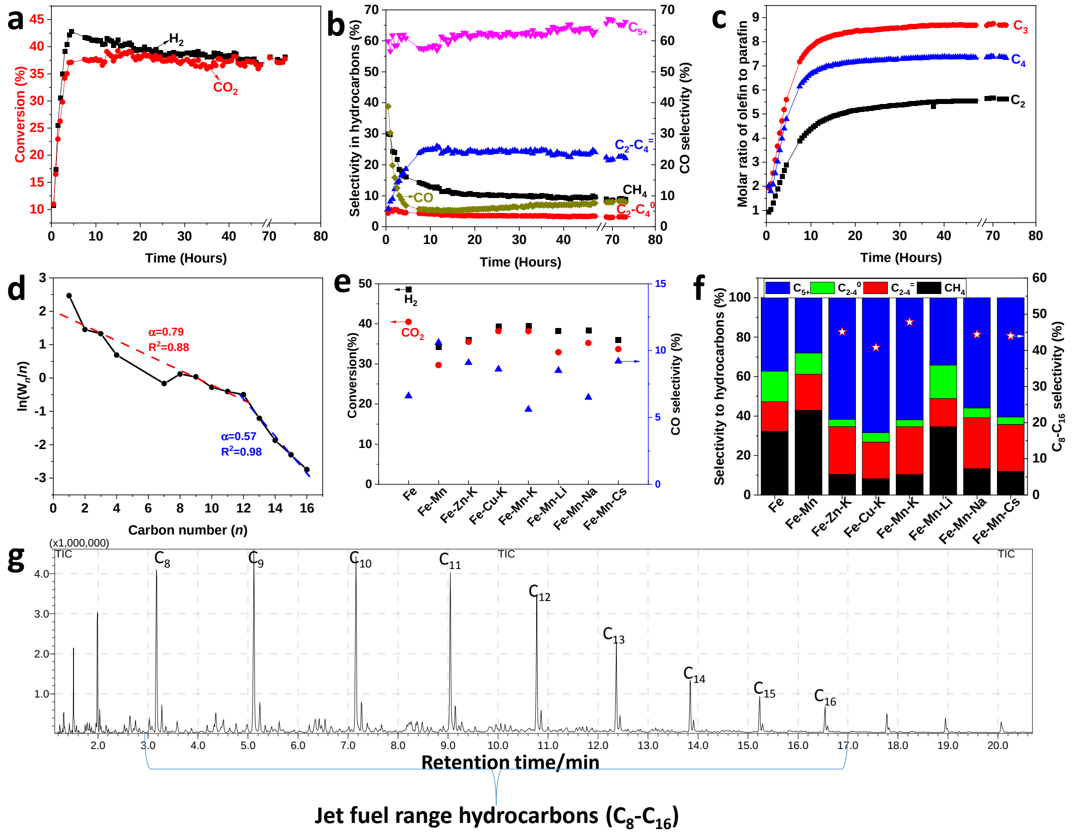

Fig. 1 Catalyst performance for the hydrogenation of carbon dioxide using a Fe-Mn-K catalyst. (**a**): % conversion of CO_2 and H_2 as a function of reaction time for the hydrogenation of CO_2. (**b**): Selectivity of various hydrocarbon products with reaction time for the hydrogenation of CO_2. (**c**): Molar ratio of olefin-to-paraffin for the C_2-C_4 range with reaction time for the hydrogenation of CO_2. (**d**): ASF plot and α values at reaction time of 20 hours. (**e**): Conversion and CO selectivity of carbon dioxide hydrogenation for a reaction time of 20 hours over different catalysts. (**f**): Products selectivities of CO_2 hydrogenation for a reaction time of 20 hours over different catalysts. (**g**): GC-MS

total ion chromatogram (TIC) of the hydrocarbon fuel from the hydrogenation of carbon dioxide on a Fe-Mn-K catalyst. The jet fuel range hydrocarbons (C_8 to C_{16}) are shown.

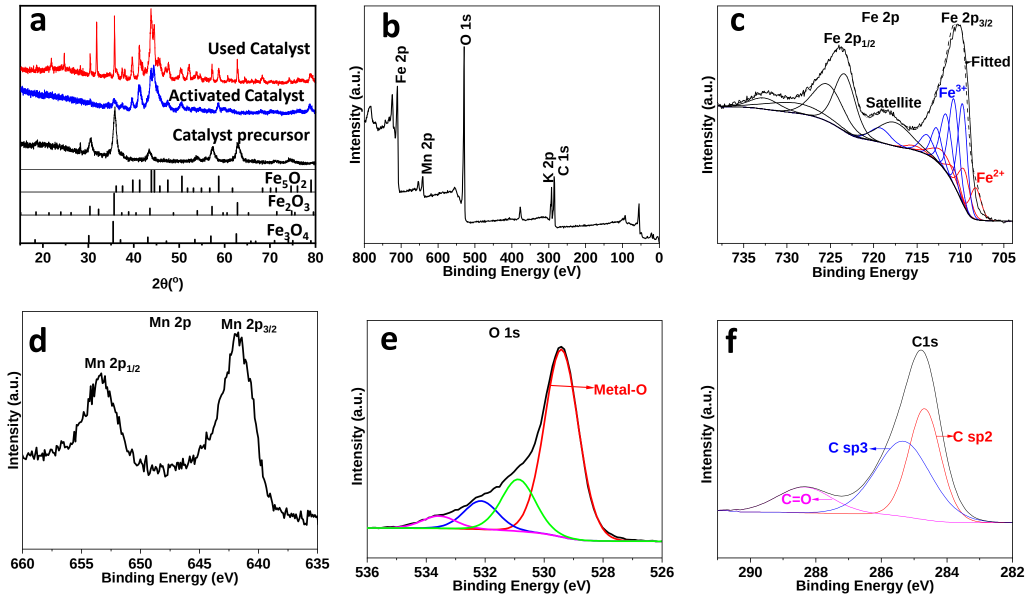

Fig. 2 XRD and XPS spectra of the Fe-Mn-K catalyst:

(**a**): Powder XRD spectra of the catalyst precursor and both the activated and the used catalyst. The corresponding JCPDS numbers are, for Fe_2O_3: 00-020-0508; χ-Fe_5C_2: 00-024-0081; Fe_3O_4: 03-065-3107; (**b**): XPS survey spectrum of the Fe-Mn-K catalyst; (**c**): High resolution XPS spectra in the region of the Fe 2p peak on the Fe-Mn-K catalyst; (**d**): XPS spectra of the Mn 2p on the Fe-Mn-K catalyst; (**e**): XPS spectra of the O 1s on the Fe-Mn-K catalyst; (**f**): XPS spectra of the C 1s on the Fe-Mn-K catalyst.

Format changes:

All changes in the manuscript were highlighted with red colour in the manuscript. The details as followed:

1. Authors:

We updated the affiliation of Xiangyu Jie, and added Prof. Peter Dobson as co-author, as he has contributed to the project during the whole process and all authors agreed.

2. Abstract:

We changed the sentence "The Fe-Mn-K catalyst, prepared by the Organic Combustion Method showed a…" as "We prepare the Fe-Mn-K catalyst by the so-called Organic Combustion Method, and the catalyst shows …"

3. Introduction:

We moved the sentence "Recently, the Organic-Combustion Method (OCM), also known as the Solution Combustion Method, has been developed to prepare highly active metal catalysts for a variety of processes." to the main text, and revised the sentences of "In this investigation, we have prepared iron-based catalysts using the OCM and utilized them for the direct and efficient conversion of carbon dioxide to jet fuel range hydrocarbons. In brief, the catalyst showed a…"
as "In this investigation, we report the preparation of iron-based catalysts using the Organic Combustion Method (OCM) and determined their catalytic performance for the direct and efficient conversion of CO_2 to jet fuel range hydrocarbons. In brief, the Fe-Mn-K catalyst shows a …"

4. Figure 3:

Figure 3 was reproduced, and caption was revised, new version as below:

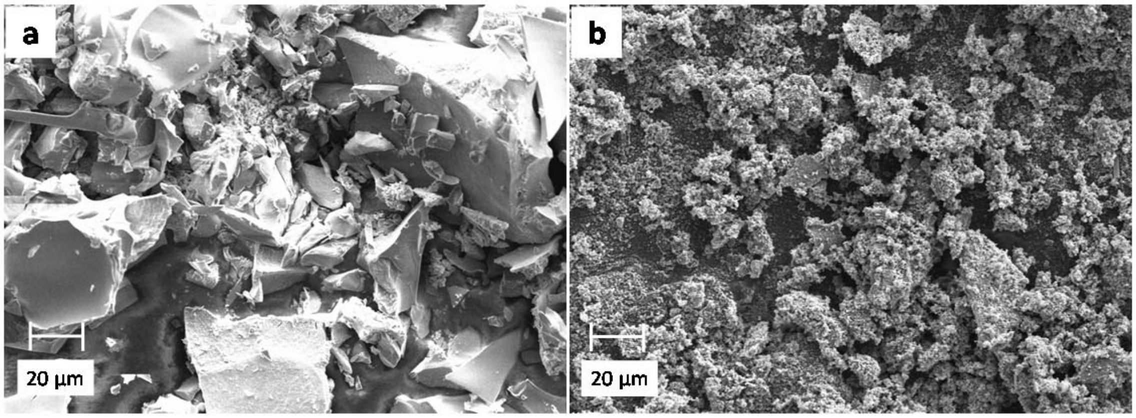

Fig. 3 SEM images of Fe-Mn-K catalysts.

a: the Fe-Mn-K catalyst precursor; b: the used Fe-Mn-K catalyst.

5. Figure 4

The caption of Figure 4 was revised as:

Fig. 4 STEM-BF images of the Fe-Mn-K catalyst at different nanoscales.

a, b, c: the Fe-Mn-K catalyst precursor; d, e, f: the used Fe-Mn-K catalyst.

6. Figure 5

The caption of Figure 5 was revised as:

Fig. 5. Reaction scheme for CO_2 hydrogenation to jet fuel range hydrocarbons.

The CO_2 hydrogenation to jet fuel range hydrocarbons process through a Tandem Mechanism in which the Reverse-Water Gas Shift reaction (RWGS) and Fischer-Tropsch synthesis (FTS) reaction are catalysed by Fe_3O_4 and χ-Fe_5C_2 respectively.

6. Figure 6

The caption of Figure 6 was revised as:

Fig. 6 Illustrating the differences of a Jet Fuel Linear and a Jet Fuel Circular Economy.

a: The Jet Fuel Linear Economy; b: the CO_2 to Jet Fuel Circular Economy.

7. References

The style of references were changed as request.

8. Competing interests

The competing interests section was revised as "The authors (B.Y., T. X., and P.P.E) have a patent application: Iron-manganese based catalyst, catalyst precursor and catalytic process, WO 2020/201749, Benzhen Yao, Peter P. Edwards and Tiancun Xiao, related to this research."

9. Supplementary information

The Supplementary information section was checked, and Figure S… were changed as Supplementary Figure … in both Supplementary information and main text.
